# Inhibition of Wnt/β-catenin increases anti-tumor activity by synergizing with sorafenib in hepatocellular carcinoma

**DOI:** 10.1038/s41419-025-07789-5

**Published:** 2025-07-01

**Authors:** Yuchao He, Dongming Liu, Aomei Ling, Zhiqiang Han, Jinfang Cui, Jinghui Cheng, Yuanying Feng, Wei Liu, Wenchen Gong, Yuren Xia, Liwei Chen, Yi Luo, Yu Wang, Xiangdong Tian, Qiang Wu, Lu Chen, Hua Guo

**Affiliations:** 1 Department of Tumor Cell Biology, Tianjin Medical University Cancer Institute and Hospital, Tianjin, China; 2 Tianjin Medical University Cancer Institute and Hospital, National Clinical Research Center for Cancer, State Key Laboratory of Druggability Evaluation and Systematic Translational Medicine, Tianjin Key Laboratory of Digestive Cancer, Tianjin’s Clinical Research Center for Cancer, Tianjin, China; 3 Department of Hepatobiliary Cancer, Liver cancer research center, Tianjin Medical University Cancer Institute and Hospital, Tianjin, China; 4 Department of Anesthesiology, Tianjin Medical University Cancer Institute and Hospital, Tianjin, China; 5 Translational Cancer Research Center, Peking University First Hospital, Beijing, China; 6 Department of Pathology, Tianjin Medical University Cancer Institute and Hospital, Tianjin, China; 7 Department of Endoscopy, Tianjin Medical University Cancer Institute and Hospital, Tianjin, China; 8 Department of Hepato-Biliary-Pancreatic Surgery, National Center for Global Health and Medicine, Tokyo, Japan

**Keywords:** Cancer therapeutic resistance, Drug development

## Abstract

Hepatocellular carcinoma (HCC) poses a major global health challenge owing to limited treatment efficacy and drug resistance to therapies such as the tyrosine kinase inhibitor (TKI) sorafenib. We utilized a microfluidic three-dimensional (3D) drug testing system to assess drug responses in 37 fresh clinical samples and performed immunohistochemical analysis of 41 tumor tissue samples that received sorafenib therapy. Results revealed that Wnt/β-catenin activation was associated with sorafenib resistance, with higher nuclear β-catenin levels predicting poor response. Targeting Wnt/β-catenin via genetic intervention enhanced TKI sensitivity by promoting apoptosis and reducing clonogenicity. Through a large scale of drug and inhibitor library screening, we identified PRI-724, a potent CREB-binding protein (CBP)/β-catenin transcription antagonist, which synergistically induces apoptosis with sorafenib in vitro and in vivo by inhibiting β-catenin/CBP/c-myc, β-catenin nuclear localization and ERK/AKT signaling. The microfluidic 3D drug testing system confirmed the synergistic anti-tumor effects of this combination, underscoring its clinical application potential. Conclusively, our study provides a new combination therapy with sorafenib and PRI-724 to overcome TKI resistance and improve clinical outcomes in patients with HCC.

**Figure:**
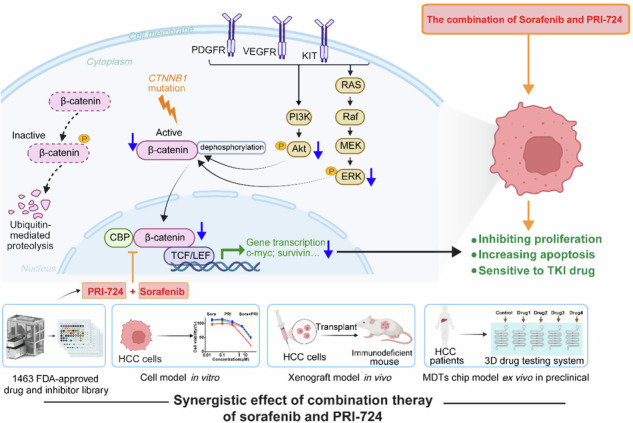
Schematic representation of the speculative molecular mechanism model. Our study revealed that β-catenin activation drives sorafenib resistance in HCC, and disrupting β-catenin enhances sorafenib efficacy by promoting apoptosis and inhibiting proliferation. The combination of sorafenib and PRI-724, a Wnt/β-catenin inhibitor, showed synergistic anti-tumor effects in vitro across various HCC cell lines, in vivo using xenograft models, ex vivo utilizing MDT chip system to explore clinical applications, offering a novel therapeutic strategy for HCC patients.

Schematic representation of the speculative molecular mechanism model. Our study revealed that β-catenin activation drives sorafenib resistance in HCC, and disrupting β-catenin enhances sorafenib efficacy by promoting apoptosis and inhibiting proliferation. The combination of sorafenib and PRI-724, a Wnt/β-catenin inhibitor, showed synergistic anti-tumor effects in vitro across various HCC cell lines, in vivo using xenograft models, ex vivo utilizing MDT chip system to explore clinical applications, offering a novel therapeutic strategy for HCC patients.

## Introduction

Primary liver cancer (PLC) is the third leading cause of cancer-related deaths. Hepatocellular carcinoma (HCC) accounts for approximately 90% of all PLC cases and is the most common histological type of liver cancer. The incidence and mortality rates of liver cancer are predicted to increase by over 50% from 2020 to 2040 [1]. Most patients with HCC are diagnosed at an advanced stage, where systemic drug therapy remains the primary therapeutic option [2]. Sorafenib, a multi-tyrosine kinase inhibitor (TKI), has been a cornerstone of advanced HCC management for over a decade [3, 4]. However, its effectiveness is hindered by high resistance and recurrence rates, resulting in an overall survival of less than 3 months [3]. These challenges underscore the urgent need to investigate resistance mechanisms and develop novel therapies or drug combinations to overcome sorafenib resistance and achieve therapeutic breakthroughs for patients with HCC.

Current research on sorafenib resistance primarily relies on cellular and mouse models due to the limitations of preclinical studies [5–7]. Few studies have investigated sorafenib resistance profiles in clinical patient tissues, leaving the underlying mechanisms of clinical resistance unclear. Additionally, no validated biomarkers have been identified to predict response to sorafenib [8, 9]. Limitations of existing preclinical models also restrict the evaluation and clinical translation of new drugs or combination therapies. From 2000 to 2015, less than 4% of anticancer drugs entering clinical trials received Food and Drug Administration (FDA) approval, reflecting the potential weaknesses of existing preclinical drug screening models [10]. Treatment responses are highly context-dependent, and existing models lack proper patient stratification to identify subgroups most likely to benefit from new treatments [11–18]. The three-dimensional (3D) drug-testing microfluidic system integrates the high biological relevance of patient-derived tumor tissues with the ease of manipulation offered by microsystems utilizing small spheroid-sized tissue samples [19]. This approach can potentially be employed throughout the drug development process, from preclinical testing to predicting clinical patient responses, thereby facilitating the selection of optimal treatment regimens.

The Wnt/β-catenin signaling pathway is crucial in embryonic development, tissue regeneration, and carcinogenesis [20]. Approximately half of the patients with HCC exhibit aberrant activation of the Wnt/β-catenin pathway, and these cases are further divided into two molecular subtypes: the CTNNB1 class (21.5%) and the Wnt-TGFβ class (27.6%). The CTNNB1 class is primarily driven by *CTNNB1* (the gene encoding β-catenin) mutations, which promote β-catenin nuclear translocation and activation of liver-specific Wnt target genes [21]. Additionally, *CTNNB1* mutations activating β-catenin are common somatic events in HCC and are associated with liver tumor progression, malignant transformation, and sorafenib resistance [22, 23]. The spectrum of CTNNB1 mutations varies between HCC and hepatocellular adenoma, with three distinct levels of β-catenin activation: (1) weak activation (S45, K335, N387 mutations), (2) moderate activation (T41 mutations), and (3) strong activation (exon 3 deletions and β-TRCP binding site mutations, D32-S37). Notably, in many HCC cases, weak S45 mutant alleles of *CTNNB1* undergo duplication, leading to markedly high β-catenin activity, which is closely associated with malignant transformation [22]. Furthermore, activation of the Wnt/β-catenin pathway has been linked to drug resistance in various cancers, including pancreatic, colon, and breast cancers [24–26]. Factors such as PROX1 and EPHB2 contribute to sorafenib resistance in HCC by activating the Wnt/β-catenin pathway [6, 27]. Targeting the Wnt/β-catenin pathway has been proposed as a potential therapeutic strategy for liver cancer. Although Wnt/β-catenin inhibitors have shown promise in preclinical studies, most remain in early clinical development. PRI-724, a small molecule specific inhibitor of the β-catenin/CREB-binding protein (CBP) interaction, selectively disrupts the interaction between β-catenin and represses a subset of TCF/b-catenin-mediated transcription and has demonstrated safety and efficacy in multiple clinical trials targeting various cancers [NCT01606579, NCT01764477]. Notably, PRI-724 has shown antifibrotic effects in patients with hepatitis C and B virus-induced liver cirrhosis [28] as well as potent anti-tumor activity in β-catenin-activated HCC [29]. However, its potential to synergize with TKIs like sorafenib remains unexplored.

This study aimed to investigate the role of the Wnt/β-catenin pathway in sorafenib resistance in HCC. We used a novel 3D drug testing system (microdissected tumor tissues [MDTs] on chip) and RNA sequencing (RNA-seq) to analyze to analyze sorafenib sensitivity and expression profiles in prospective fresh surgical samples from 37 liver cancer patients. Additionally, we retrospectively conducted immunohistochemistry (IHC) analysis on paraffin-embedded tissues from patients with advanced HCC patients treated with sorafenib to assess the association with Wnt/β-catenin activation and sorafenib resistance. Biological experiments evaluated whether genetically inhibiting Wnt/β-catenin signaling enhances sorafenib efficacy. A fully automated PerkinElmer G3 integrated system was employed for high-throughput drug screening to identify a promising candidate that inhibits Wnt/β-catenin signaling and increases sorafenib sensitivity. These approaches, we seek to provide insights into overcoming resistance mechanisms and identify a promising therapeutic strategy combining PRI-724 and TKI therapy for HCC treatment.

## Results

### 3D drug testing work system: MDTs on chip

**Fig. 1:**
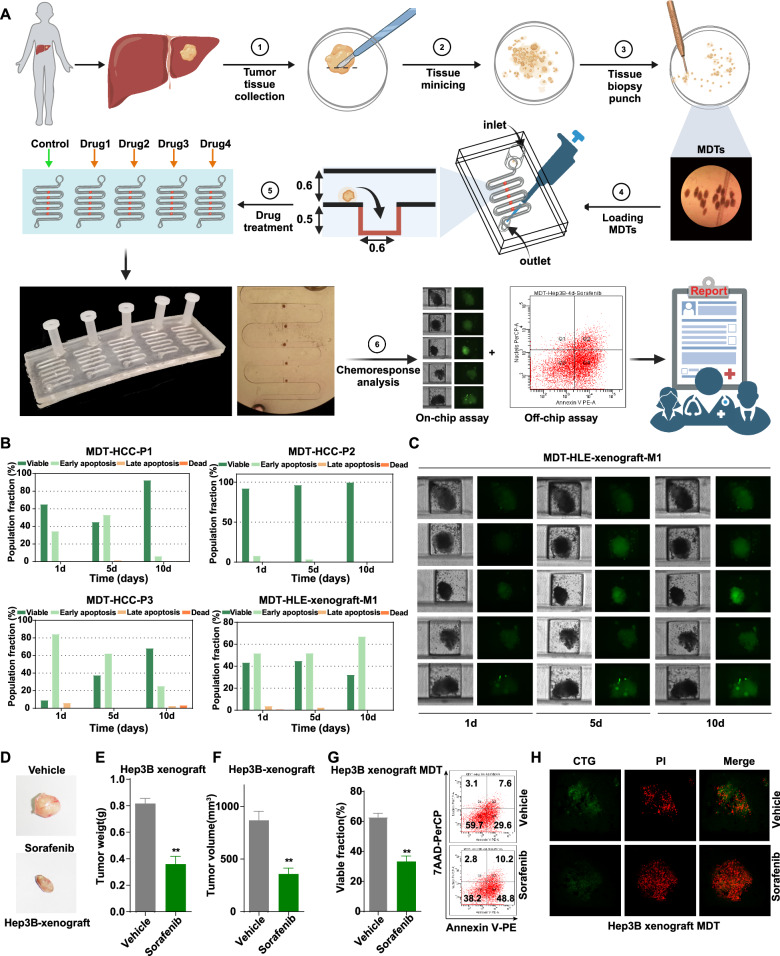
3D drug testing work system: micro-dissected tumor tissues (MDT) on a chip. **A** The workflow of the microfluidic device for 3D MDT culture and drug testing. **B** Annexin V-PE/7-AAD staining by flow cytometry was performed to detect the mean apoptosis rates of MDTs from patients (P1, P2, and P3) and HLE xenograft tumor tissues (M1). Early apoptosis (Annexin V-PE^+^/7-AAD^-^), late apoptosis (Annexin V-PE^+^/7-AAD^+^), and necrosis (Annexin V-PE^-^/7-AAD^+^). **C** The viability of MDTs on chip from HLE xenograft tumor tissues by CTG staining. **D** The tumor images, **E** tumor weight, and **F** tumor volume of Hep3B subcutaneous xenografts with or without treatment with 30 mg/kg sorafenib (*n* = 4/group). **G** The apoptosis analysis of Hep3B xenograft MDTs treated with or without 5 μM sorafenib. **H** The viability analysis on chip detected by CTG and PI staining. The error bars indicate the means ± SEM; ** *p* < 0.01, *t* test (**E**–**G**).

To overcome the limitations of current tumor drug-screening platforms and meet the urgent need for effective anticancer drugs, we employed an ex vivo drug testing and personalized therapy system utilizing MDTs on a microfluidic chip. Fresh surgical tumor samples from liver cancer patients were minced and processed with a 500-μm tissue punch to generate MDTs (Fig. 1A). These MDTs were loaded into a microfluidic device designed to accommodate 25 MDTs concurrently, thereby enabling exposure to five distinct chemotherapeutic drugs or dosages simultaneously. The device further incorporated five inlet reservoirs to continuously supply culture media, ensuring constant perfusion and nutrient delivery to the MDTs.

After drug treatment, the biological activity of the tumor samples was evaluated using both on-chip analysis confocal microscopy, and off-chip analysis, flow cytometry to determine the response rates to different drugs. Tissue viability was maintained within the microsystem for 8–10 days with regular medium replacement, using MDTs derived from tissues from three patients with liver cancer (P1–P3) and xenografts from the HCC cell line, HLE (M1), as confirmed by flow cytometric assay (Fig. 1B). On-chip viability staining results further demonstrated that MDTs remained viable after a 10-day culture period on the platform (Fig. 1C). These findings were consistent with Professor Anne-Marie Mes-Masson’s research on ovarian and prostate cancer tissues [19], confirming the model’s ability to maintain tissue viability and successfully culture tumor tissues. Additionally, this model supports the culture of submillimeter-sized 3D tumor samples ex vivo, making it particularly suitable for clinical biopsy samples with small volumes.

To assess the ex vivo model’s predictive accuracy, we compared in vivo and ex vivo drug responses. In vivo, subcutaneous xenografts derived from Hep3B cells showed significant reductions in tumor volume and weight after sorafenib treatment (Fig. 1D–F). Ex vivo, MDT viability decreased significantly, as demonstrated by off-chip and on-chip analyses, with a concurrent rise in apoptosis following sorafenib treatment (Fig. 1G–H). This likely reflects the model’s capacity to preserve tissue integrity and simulate the tumor microenvironment, resulting in outcomes that mirror in vivo drug efficacy. Overall, these findings indicate that the MDT chip system is an effective platform for 3D drug testing, enabling multidimensional tumor drug screening and efficacy prediction with small tissue samples, short experimental cycles, and high throughput.

### Wnt/β-catenin activation is associated with sorafenib resistance in liver cancer

**Fig. 2:**
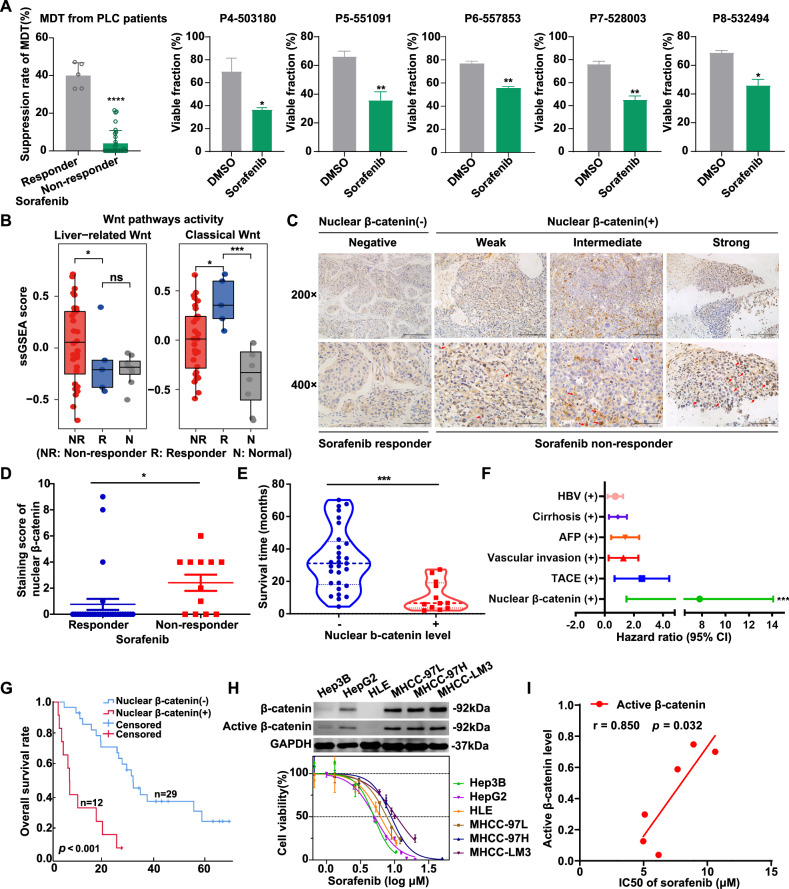
Wnt/β-catenin activation is associated with sorafenib resistance in HCC. **A** Statistical analysis of sorafenib’s suppression effect on MDTs from 37 HCC patients divided into responder and non-responder groups. Five out of 37 MDTs responded to sorafenib based on the viable fraction after 5 μM sorafenib treatment for 7 days. **B** The ssGSEA score of Wnt pathway activity including liver-related Wnt target genes signal and classical Wnt target genes signal, in the tissues of the 37 MDTs from liver cancer patients. **C** The representative IHC images of β-catenin in sorafenib responder and non-responder HCC patients. Scale bars, 200 µm for 200×; 100 µm for 400×. **D** The comparison of the IHC score of nuclear β-catenin between sorafenib responder and non-responder HCC patients. **E** The comparison of survival time between 41 HCC patients with negative and positive nuclear β-catenin expression. **F** Identification of independent risk factors for OS in the cohort of 41 HCC cases according to multivariate analysis. **G** Kaplan–Meier plots comparing 5-year OS (*p* < 0.001) rates between patients with negative vs. positive nuclear β-catenin expression. **H** The β-catenin and active β-catenin levels and IC50 of sorafenib in various HCC cell lines detected by western blotting assay and CCK-8 assay, respectively. **I** The association analysis between active β-catenin level and IC50 of sorafenib in various HCC cell lines. The error bars indicate the means ± SEM; ns: *p* > 0.05, * *p* < 0.05, ** *p* < 0.01, *** *p* < 0.001, **** *p* < 0.0001, *t* test (**A**, **B**, and **D**–**F**).

To investigate the underlying causes of sorafenib’s limited clinical efficacy and the mechanisms of sorafenib resistance in patients with liver cancer, we utilized the MDT chip system to evaluate the response of 37 surgically resected liver cancer tissue samples to sorafenib. After 7 days of treatment with the standard clinical dose of sorafenib (10 μM), patients were classified as responders or non-responders based on flow cytometric analysis of MDTs. Sorafenib responders were defined as those whose MDTs exhibited a suppression rate exceeding 20% and differed significantly from the dimethyl sulfoxide (DMSO) group. This yielded a clinical effectiveness rate of 13.5% (5/37) (Fig. 2A). This observation is consistent with the reported clinical overall response rate (ORR) of sorafenib (9.2%) [30], validating the accuracy of the MDT chip system for drug evaluation. Additionally, Hepar1^+^ hepatocytes, α-SMA^+^ fibroblasts, and CD45^+^ immune cells were observed in MDTs at both time points (Supplemental Fig. 1A), reflecting microenvironmental adaptation in vitro. These findings validate that the MDT model preserves the heterogeneity of HCC, including tumor, stromal, and immune components, making it a biologically relevant system for studying drug responses.

To further explore the gene expression profiles associated with sorafenib resistance, we conducted RNA-seq of tumor tissues from these patients, comparing the responsive and non-responsive groups, with eight para-cancer tissues used as controls. RNA-seq analysis revealed differentially expressed genes (DEGs) between the two groups with distinct enrichment in the Wnt pathway signals (Supplemental Fig. 2A). Liver-related Wnt” signaling was primarily increased in non-responder samples, while “Classical Wnt” signaling was enriched in the responder groups (Fig. 2B). “Liver-related Wnt” signaling is a characteristic feature of the “CTNNB1 class,” often involving enrichment in nuclear β-catenin staining and *CTNNB1* mutations [21], which are linked to sorafenib resistance and immune evasion [9, 22, 23].

To confirm the association between sorafenib resistance and β-catenin activation, we analyzed 41 HCC patients treated with sorafenib (2009–2018) and classified them as responders and non-responders based on clinical response. IHC staining revealed that elevated nuclear β-catenin levels were enriched in non-responders (Fig. 2C, D). Furthermore, patients with positive nuclear β-catenin expression exhibited shorter survival times despite treatment (Fig. 2E). Multivariate analysis identified nuclear β-catenin activation as an independent prognostic factor for HCC patients treated with sorafenib (Fig. 2F and Supplementary Table 3), with higher levels correlating with shorter overall survival and poorer prognosis (Fig. 2G).

Furthermore, the positive correlation between active β-catenin level and sorafenib IC50 values was validated in various HCC cell lines (Fig. 2H, I). The GEPIA database analysis confirmed a positive correlation between CTNNB1 and the multidrug resistance markers *ABCB1* and *ABCC1* [31] (Supplementary Fig. 2B, C). Additionally, drug sensitivity testing and expression profiling of 255 HCC organoids [32] demonstrated that β-catenin expression levels were positively correlated with sorafenib resistance (Supplementary Fig. 2D). These results indicate that Wnt/β-catenin activation is associated with sorafenib resistance in HCC.

### Disruption of β-catenin increases tumor cells’ susceptibility to sorafenib by promoting apoptosis and inhibiting cell growth

**Fig. 3:**
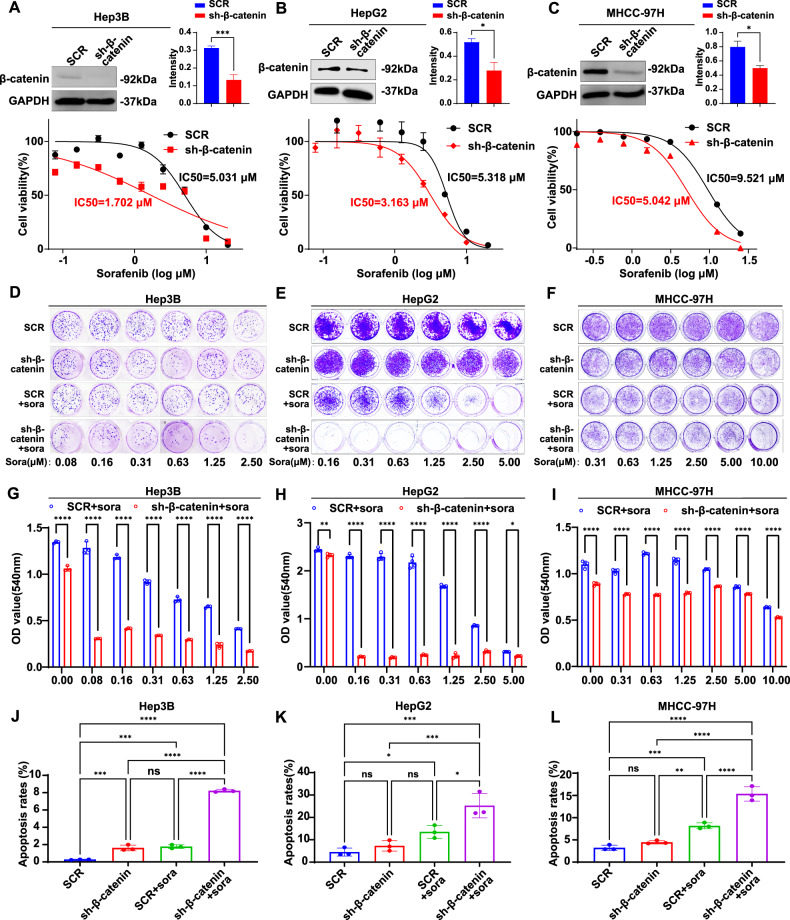
β-catenin regulates resistance to sorafenib in HCC. Western blot and the quantified intensities of bands showing β-catenin expression in β-catenin-knockdown Hep3B cells (**A**), HepG2 cells (**B**), and MHCC-97H cells (**C**). β-catenin-knockdown cells and SCR control cells were treated with the indicated concentrations of sorafenib for 48 h and subjected to a cell viability assay. β-catenin-knockdown cells and SCR control cells were treated with or without the indicated concentrations of sorafenib for 8 days in Hep3B cells (**D**), 14 days in HepG2 (**E**), and MHCC-97H cells (**F**), and then subjected to a clonogenic cell survival assay. Quantitation of clonogenic cells from three independent experiments is shown for Hep3B (**G**), HepG2 (**H**), and MHCC-97H (**I**). After treatment with or without sorafenib for 48 h, β-catenin-knockdown cells and SCR control cells were double-stained with Annexin V-PE/7-AAD apoptosis kit and analyzed by flow cytometry: 1.25 μM sorafenib for Hep3B cells (**J**), 2.5 μM for HepG2 cells (**K**), and 5 μM for MHCC-97H cells (**L**). The error bars indicate the means ± SEM; ns: *p* > 0.05, *p < 0.05, **p < 0.01, ***p < 0.001, ****p < 0.0001, *t* test (**A**–**C**) two-way ANOVA (**G**–**I**), one-way ANOVA (**J**–**L**).

To confirm the role of β-catenin in sorafenib sensitivity, we generated stable β-catenin knockdown cell lines (sh-β-catenin) and control cell lines (SCR) in sorafenib-sensitive Hep3B, moderately sensitive HepG2, and resistant MHCC-97H cells. Western blotting confirmed the efficiency of β-catenin knockdown in all three cell lines. Compared to the SCR groups, β-catenin knockdown significantly reduced the IC50 values of sorafenib, from 5.031 to 1.703 μM in Hep3B cells (Fig. 3A), from 5.318 to 3.163 μM in HepG2 cells (Fig. 3B), and from 9.521 to 5.042 μM in MHCC-97H cells (Fig. 3C).

Clonogenic assays revealed that β-catenin knockdown slightly reduced colony formation compared to the SCR group. Notably, following sorafenib treatment, a dose-dependent increase in sorafenib concentration resulted in a significantly greater reduction in colony forming in a sh-β-catenin group compared to the SCR group (Fig. 3D–F). Additionally, β-catenin knockdown increased sorafenib’s inhibitory effect on colony formation even at low concentrations: Hep3B (0.08 μM) (Fig. 3D, G), HepG2 (0.16 μM) (Fig. 3E, H), and MHCC-97H (0.63 μM) (Fig. 3F, I). This enhanced the cell’s susceptibility to sorafenib. Additionally, β-catenin knockdown significantly increased the rate of sorafenib-induced apoptosis in Hep3B (Fig. 3J), HepG2 (Fig. 3K), and MHCC-97H cells (Fig. 3L) compared to the SCR group. These results suggest that β-catenin activity determines sorafenib’s response in HCC cells, and its knockdown enhances sorafenib susceptibility by promoting apoptosis and inhibiting cell growth.

### Identification of Wnt/β-catenin inhibitor PRI-724 as a sensitizer for sorafenib from FDA-approved drug and small-molecule inhibitor library

**Fig. 4:**
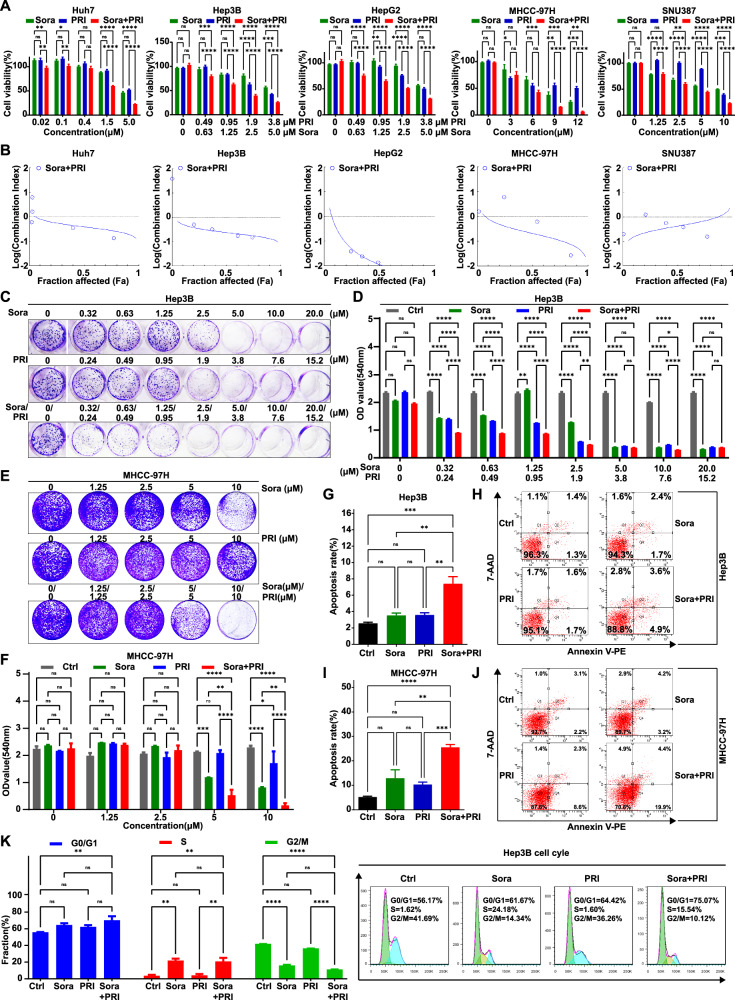
Synergistic effect of combination treatment of sorafenib and β-catenin inhibitors, PRI-724. **A** The cell viability assay was performed for Huh7 cells, Hep3B cells, HepG2 cells, MHCC-97H cells, and SNU387 cells after treatment with the indicated concentrations of sorafenib, PRI-724, and the combination for 48 h (sample size, *n* = 6). **B** The synergistic effect of sorafenib and PRI-724 was shown by combination index based on the above cell viability assay results in various HCC cells. **C**–**F** The synergistic effect of β-catenin inhibitor PRI-724 and sorafenib was measured by a clonogenic cell survival assay following an 8-day treatment in Hep3B cells (C&D) and a 14-day treatment in MHCC-97H cells (E&F). Representative images and quantitative clonogenic cells of three independent experiments are shown. Hep3B cells were treated with 1.25 μM sorafenib, 0.95 μM PRI-724, or their combination (**G**, **H**); MHCC-97H cells were treated with 5 μM sorafenib, 5 μM PRI-724, or their combination (**I**, **J**). The apoptosis rate and representative images by flow cytometric analysis are shown. **K** The cell cycle distribution of Hep3B cells treated with 1.25 μM sorafenib, 0.95 μM PRI-724, or their combination. *n* = 3 for **B**–**K**. The error bars indicate the means ± SEM; ns: *p* > 0.05, *p < 0.05, **p < 0.01, ***p < 0.001, ****p < 0.0001, two-way ANOVA (F–J, Q, and S), one-way ANOVA (T and V).

To identify potential drugs capable of overcoming sorafenib resistance in HCC, we employed a combination treatment strategy using a drug library of 1463 FDA-approved compounds and a small-molecule inhibitor library (Supplementary Table 4) to identify agents with sorafenib-sensitizing properties in MHCC-97H cells. We assessed relative cell viability after treatment with candidate drugs with or without sorafenib to identify compounds that acted synergistically with sorafenib (Supplementary Table 4). Notably, the Wnt/β-catenin inhibitor PRI-724 emerged as particularly effective in enhancing the antitumor effects of sorafenib in MHCC-97H cells (Supplementary Fig. 3). PRI-724 significantly inhibited cell growth in a dose-dependent manner across various HCC cell lines, including the relatively sorafenib-sensitive Huh7 and Hep3B cells, moderately sensitive HepG2 cells, and sorafenib-resistant MHCC-97H and SNU387 cells (Fig. 4A and Supplementary Table 5). This indicates a substantial synergistic effect on cellular proliferation when PRI-724 was combined with sorafenib in different HCC cell models in vitro (Fig. 4B). Clonogenic assays further confirmed that combining PRI-724 and sorafenib significantly inhibited colony formation in Hep3B (Fig. 4C, D) and MHCC-97H cells (Fig. 4E, F). Flow cytometry apoptosis assays showed that the combination treatment markedly increased apoptosis rates in Hep3B (Fig. 4G, H) and MHCC-97H cells (Fig. 4I, J) compared to the control and single-agent therapies. Additionally, the combination therapy of sorafenib and PRI-724 significantly increased the proportion of cells in the G0/G1 phase while reducing the G2/M-phase fraction, suggesting a cell cycle arrest effect (Fig. 4K).

Overall, these results suggest that PRI-724, a β-catenin/CBP antagonist, represents a promising candidate for combination therapy with sorafenib. This approach offers a potentially universal strategy for augmenting antitumor efficacy and inducing apoptosis in HCC cells in vitro.

### Combinations of PRI-724 and sorafenib inhibit both β-catenin/CBP and ERK/AKT signaling and decrease β-catenin nuclear localization in HCC cells

**Fig. 5:**
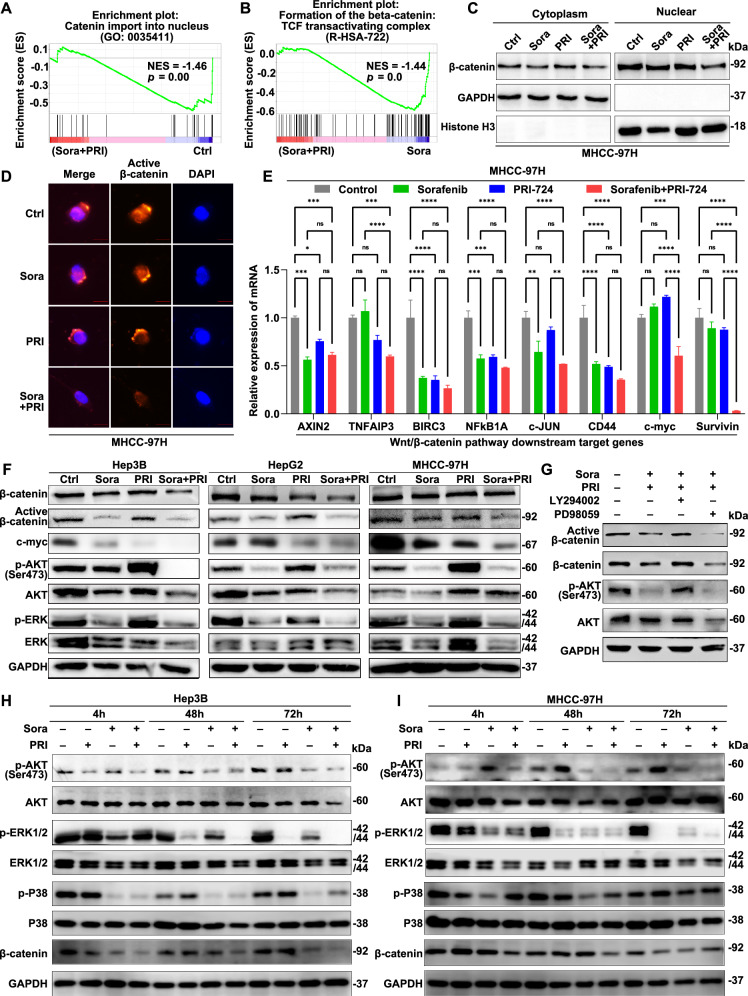
Combinations of PRI-724 and sorafenib inhibit both β-catenin/CBP and ERK/AKT signaling and decrease β-catenin nuclear localization in HCC cells. **A** GSEA of catenin import into nucleus signal in Hep3B cells with combination treatment with 2.5 μM sorafenib and 1.9 μM PRI-724 compared to the control group. **B** GSEA of formation of the β-catenin: TCF transactivating complex signal in Hep3B cells with combination treatment with 2.5 μM sorafenib and 1.9 μM PRI-724 compared to the 2.5 μM sorafenib group. **C** Western blot for β-catenin in the cytoplasmic and nuclear fractions according to the indicated groups in MHCC-97H cells. Sorafenib, 5 μM for 24 h; PRI-724, 5 μM for 24 h. Histone H3 was used as the nuclear marker. **D** Active β-catenin protein expression patterns and localization were assessed by IF staining in MHCC-97H cells treated with or without 5 μM sorafenib, 5 μM PRI-724, and their combination for 24 h. Scale bars, 10 µm. **E** Quantitative RT-PCR of the Wnt/β-catenin downregulation target genes mRNA after 24 h treatment of sorafenib (5 μM), PRI-724 (5 μM), and their combination. **F** Expression of Wnt/β-catenin/CBP, ERK/p-ERK, and AKT/p-AKT signaling and downstream target c-myc were analyzed by western blotting in Hep3B, HepG2, and MHCC-97H cells treated with PRI-724 and sorafenib for 24 h. PRI-724 and sorafenib were 1.9 μM and 2.5 μM for Hep3B and HepG2, and 5 μM and 5 μM for MHCC-97H cells, respectively. **G** The level of active β-catenin, β-catenin, and AKT/p-AKT with or without the AKT inhibitor LY294002 or the ERK inhibitor PD98059 under the combination treatment condition in Hep3B cells. **H** Hep3B cells were treated with PRI-724 (1.9 μM) and sorafenib (2.5 μM), and (**I**) MHCC-97H cells were treated with PRI-724 (5 μM) and sorafenib (5 μM) for 4, 48, and 72 h. The protein expression was assessed by western blotting analysis, including β-catenin, ERK/p-ERK, AKT/p-AKT, and P38/p-P38 signaling. The error bars indicate the means ± SEM; ns: *p* > 0.05, *p < 0.05, **p < 0.01, ***p < 0.001, ****p < 0.0001, two-way ANOVA (**E**).

RNA-seq analysis on Hep3B cells treated with a low dose of sorafenib (2.5 μM), PRI-724 (1.9 μM), the combination treatment, and a control group revealed that only the combination treatment significantly reduced Wnt/β-catenin nuclear import. This was indicated by the enrichment of the “catenin import to nucleus” gene set in the control group compared to the combination treatment, but not when compared to the single-drug groups (Fig. 5A). Furthermore, the combination treatment group exhibited a negative correlation with the “Formation of β-catenin/TCF transactivation complex” gene set compared to the sorafenib group. This suggests that the combination treatment inhibited the formation of the β-catenin/TCF complex in the nucleus, thereby attenuating Wnt/β-catenin signal activation (Fig. 5B). Western blotting analysis of nuclear and cytoplasmic fractions showed that sorafenib and PRI-724 combination treatment significantly decreased β-catenin levels in the nucleus (Fig. 5C and Supplementary Fig. 4A and Supplementary Fig. 5A). Immunofluorescence staining further demonstrated that the combination treatment significantly inhibited the level of active β-catenin and reduced its nuclear localization (dephosphorylation at Ser37/Thr41) (Fig. 5D and Supplementary Fig. 5B, C), which decreases cytoplasmic stability, induces β-catenin translocation into the nucleus and increases its transcriptional activity [33]. This is consistent with prior study that the decrease in cytoplasmic active β-catenin is consistent with increased nuclear export of β-catenin and subsequent degradation in the cytoplasm [34]. Quantitative real-time PCR (qRT-PCR) results indicated that combination treatment significantly reduced the expression of downstream target genes of the Wnt/β-catenin pathway, including *AXIN2*, *TNFAIP3, BIRC3, NFκBIA, c-JUN, CD44, c-myc, and survivin*, compared to the control group. Specifically, optimal inhibition of *c-myc* and *survivin* expression, which are involved in the proliferation and transformation [35], occurred only with the combined use of sorafenib and PRI-724 and not with either drug alone (Fig. 5E and Supplementary Fig. 5D). Western blotting also confirmed that the level of active β-catenin and c-myc was significantly inhibited by the combination treatment in Hep3B, HepG2, and MHCC-97H cells (Fig. 5F and Supplementary Fig. 4B–D). This was particularly evident alongside potent inhibition of ERK/p-ERK and AKT/p-AKT signaling (Fig. 5F).

The findings suggest that, in addition to modulating *c-myc* through the β-catenin nuclear translocation, combination therapy may also regulate β-catenin activation and c-myc levels through p-ERK or p-AKT signaling. To test this, we treated Hep3B cells with the AKT inhibitor LY294002 or the ERK inhibitor PD98059 under the combination treatment condition. We found that PD98059, but not LY294002, suppressed β-catenin, especially active β-catenin levels (Fig. 5G and Supplementary Fig. 4E), suggesting that the combination treatment-induced inhibition of c-myc or β-catenin is partially mediated through ERK/p-ERK signaling. This finding aligns with studies showing that the PI3K-AKT-mTOR and RAF/MEK/ERK pathways contribute to β-catenin activation in other tumor types [36–38]. The Liver Cancer Cell Lines Database records indicate that MHCC-97H cells have a *CTNNB1* mutation, whereas Hep3B cells do not. We found that sorafenib alone does not effectively inhibit active β-catenin in MHCC-97H cells with *CTNNB1* mutation; however, the combination with PRI-724 achieves significant synergistic suppression of active β-catenin and c-myc (Fig. 5F), highlighting the crucial role of PRI-724. PRI-724 directly inhibits CBP/β-catenin, while sorafenib indirectly inhibits β-catenin through the PI3K/Akt or RAS-RAF/MEK/ERK pathways, explaining the observed synergistic effect.

Time-dependent drug treatment experiments confirmed that combination therapy significantly inhibited total β-catenin, AKT/p-AKT, and ERK/p-ERK signaling in both CTNNB1 wild-type Hep3B cells and CTNNB1 mutant MHCC-97H cells, independent of *CTNNB1* mutation status (Fig. 5H, I and Supplementary Fig. 4F–K). However, inhibition of the P38/p-P38 signaling pathway was observed only in CTNNB1 wild-type Hep3B cells and not in MHCC-97H cells (Fig. 5H, I), suggesting that P38 signaling regulation by combination therapy may be influenced by *CTNNB1* mutation or other feedback regulation mechanisms, warranting further investigation.

Sorafenib, a multikinase inhibitor, suppresses tumor growth and angiogenesis by targeting RAF kinases (inhibiting RAF/MEK/ERK signaling activation) and receptor tyrosine kinases (e.g., VEGFR, PDGFR). By inhibiting RAF/MEK/ERK signaling, sorafenib reduces the activation of downstream signaling molecules, such as ERK and AKT, which are crucial for tumor progression and resistance mechanisms in HCC [39, 40]. Interestingly, compared to the control group, p-AKT levels were suppressed in both the combination treatment and sorafenib monotherapy groups but significantly increased after 24 h of PRI-724 treatment (Fig. 5F). This increase was gradual from 4 h to 72 h of PRI-724 treatment (Fig. 5H, I). Notably, sorafenib reversed the pro-phosphorylation effect of PRI-724 on AKT, with their combination synergistically inhibiting AKT phosphorylation at Ser473 (Fig. 5H, I), which is associated with the promotion of tumor cell viability [41–43]. The addition of the AKT inhibitor LY294002 to the combination treatment group rescued AKT phosphorylation to pretreatment levels (Fig. 5G). Moreover, LY294002 enhances AKT phosphorylation in gemcitabine-resistant pancreatic cancer cells by partially occupying the PI3K active site, which may inadvertently activate other signaling pathways [44].

In summary, combination therapy primarily enhances anti-tumor activity by inhibiting the Wnt/β-catenin/CBP/c-myc signal, β-catenin nuclear translocation, and the AKT/p-AKT and ERK/p-ERK signaling pathways, independent of CTNNB1 mutation. The inhibition of the Wnt/β-catenin/CBP/c-myc pathway is mediated in part through ERK signaling rather than AKT signaling.

### Inhibition of β-catenin activity enhances the antitumor activity of sorafenib against HCC xenografts in vivo and MDTs derived from patients with liver cancer in the clinic

**Fig. 6:**
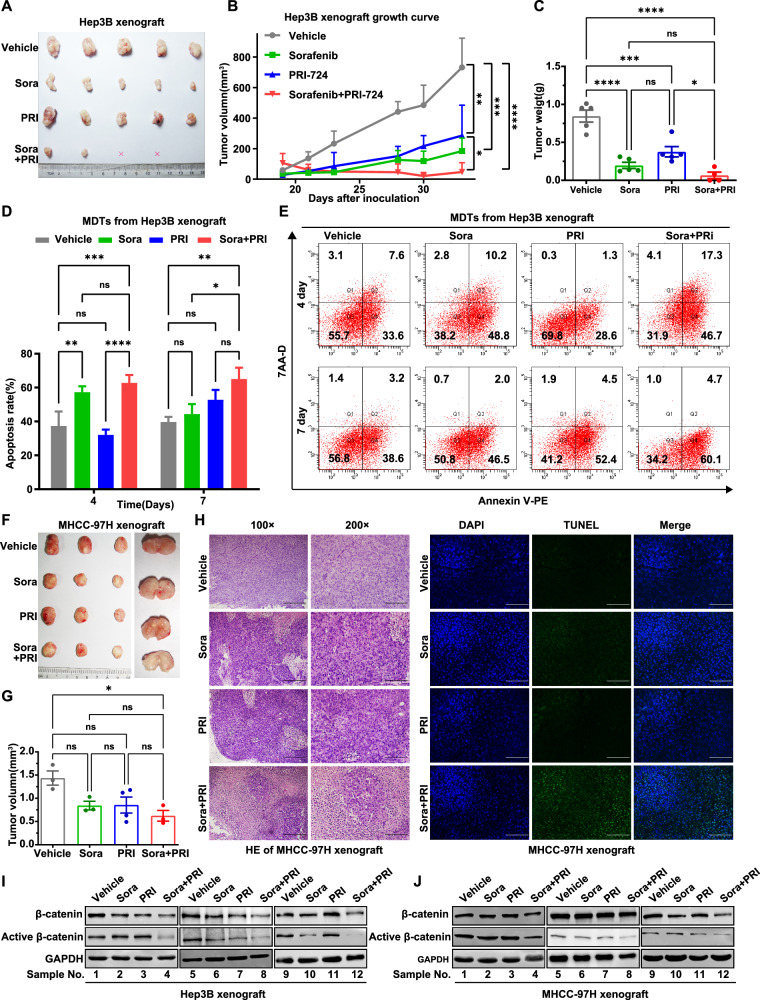
Inhibition of β-catenin activity enhances the antitumor activity of sorafenib against HCC xenografts. **A** Image of the xenograft tumors formed by Hep3B cells treated with 30 mg/kg sorafenib and 50 mg/kg PRI-724 or their combination (*n* = 5). “×” indicates that the mouse tumor disappeared by the end of the drug treatment. Additionally, in the combination group, one mouse died due to the infusion technique on the second day of administration. **B** The growth curve and (**C**) tumor weight of the Hep3B xenograft tumors in the indicated 4 groups. **D**, **E** Annexin V assays followed by flow cytometry were used to evaluate the apoptosis rate of MDTs from Hep3B xenograft tissues treated with PRI-724 (3.8 μM) and sorafenib (5 μM) for 4 and 7 days. **F** Image of the xenograft tumors formed by MHCC-97H cells treated with 30 mg/kg sorafenib and 50 mg/kg PRI-724 or their combination (*n* = 3). **G** Tumor weight of MHCC-97H xenograft tumors at endpoint in the indicated 4 groups. **H** H&E and TUNEL staining image in MHCC-97H xenograft tumor samples after treatment with sorafenib, PRI-724, or the combination. Scale bars, 400 µm for 100×; 200 µm for 200×. The total and active β-catenin levels were assessed by western blotting assay in Hep3B xenograft tumor tissues (**I**) and MHCC-97H tumor tissues (**J**) after the indicated treatment (sample numbers = 12, *n* = 3/group). The error bars indicate the means ± SEM; ns: *p* > 0.05, *p < 0.05, **p < 0.01, ***p < 0.001, ****p < 0.0001, two-way ANOVA (**B**–**D**, **G**).

We established two HCC xenograft murine models to evaluate the efficacy of sorafenib and PRI-724 combination in vivo. In the Hep3B xenograft tumor model, the combination treatment significantly inhibited tumor growth compared to either drug alone or the control vehicle group, with complete tumor regression observed in two mice (Fig. 6A). The combination treatment group showed significantly reduced tumor volume and weight compared to the control and monotherapy groups (Fig. 6B, C), with no notable changes in mouse body weight before and after treatment, suggesting good safety (Supplementary Fig. 6A). Additionally, no significant differences were observed in the weight of liver or spleen among the groups, suggesting no overt systemic toxicity associated with either sorafenib, PRI-724, or their combination in Hep3B and MHCC-97H xenograft tumor models (Supplementary Fig. 6B). Consistent synergistic effects were confirmed using a 3D drug testing system with MDTs from Hep3B xenograft tumors, showing increased apoptosis rates after a 7-day combination treatment with sorafenib and PRI-724 (Fig. 6D, E and Supplementary Fig. 6C). In the MHCC-97H subcutaneous xenograft model, the combination treatment reduced tumor volume compared to the control group (Fig. 6F, G). Although tumor reduction was less pronounced than that in the Hep3B model, macroscopically visible white necrotic regions and a paler color were observed in tumor tissues from the combination treatment group (Fig. 6F).

HE staining confirmed more extensive necrotic regions and higher tumor cell apoptosis in the combination treatment group compared to the control and monotherapy groups (Fig. 6H and Supplementary Fig. 6D, E). Following combination treatment, western blotting demonstrated a more significant synergistic reduction in active β-catenin levels in tumor tissues compared to total β-catenin levels from both xenograft models (Fig. 6I, J and Supplementary Fig. 7) and HCC cells (Fig. 5F).

**Fig. 7:**
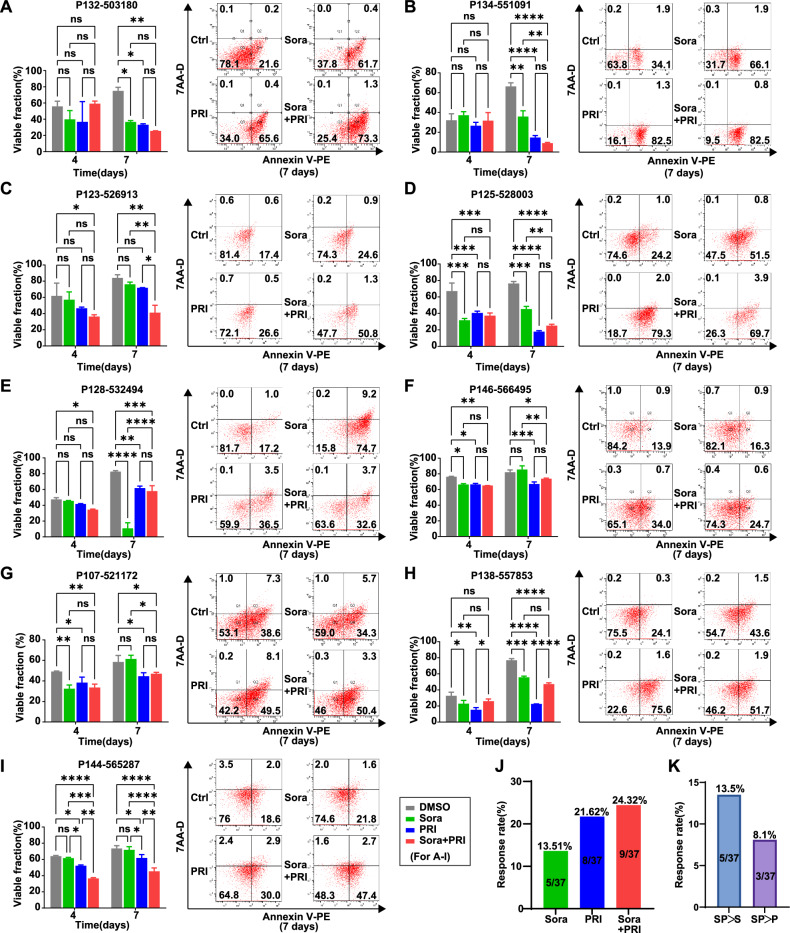
Exploration in clinical application of combination of sorafenib and PRI-724. **A**–**I** The MDT samples from 37 HCC patients were subjected to 3D culture and drug sensitivity testing using the MDT chip system treated with PRI-724 (5 μM) and sorafenib (5 μM) for 4 and 7 days. The results for the 9 cases that responded to single or combination drug treatments are shown by the apoptosis rates analysis. **J** The response rate of sorafenib, PRI-724, and combination therapy in 37 MDTs from HCC patients. **K** Statistical analysis of response rates shows that combination therapy is superior to single-agent treatments with sorafenib or PRI-724. The error bars indicate the means ± SEM; ns: *p* > 0.05, *p < 0.05, **p < 0.01, ***p < 0.001, ****p < 0.0001, two-way ANOVA (**A**–**I**).

To further explore the clinical application of the combination treatment, we employed a 3D drug testing system to evaluate drug responsiveness utilizing MDTs derived from 37 freshly resected liver cancer tissue samples. The results demonstrated that the combination treatment exhibited the strongest anti-tumor activity, as measured by flow cytometry (Fig. 7A–I). Of the 37 patient samples, nine responded to the combination therapy (24.32%), eight to PRI-724 alone (21.62%), and five to sorafenib alone (13.51%) (Fig. 7J, Supplementary Fig. 8, and Table 6). Notably, five patients (13.5%) responded better to the combination treatment than to sorafenib alone, and three patients (8.1%) responded better to the combination than to PRI-724 alone (Fig. 7K). Additionally, the phosphorylation of both ERK and AKT was reduced upon co-treatment with sorafenib and PRI-724 compared to control groups (Supplemental Fig. 9), suggesting that PRI-724, when combined with sorafenib, may downregulate the activation of these key survival and proliferation pathways, contributing to the enhanced therapeutic efficacy. These findings suggest that combination therapy may be a potential strategy to enhance sorafenib response rates and clinical benefit in patients with liver cancer.

## Discussion

Liver cancer, particularly HCC, remains a major global health challenge with limited therapeutic benefits [45]. Notably, sorafenib’s efficacy is restricted by inherent and acquired resistance, highlighting the need for alternative or complementary therapies. Our study demonstrates that β-catenin activation is a key mediator of sorafenib resistance in HCC. Both the MDTs and retrospective immunohistochemistry analysis support that higher nuclear β-catenin levels predict a poor response to sorafenib. This is especially relevant for patients with CTNNB1 mutations, where aberrant β-catenin activation drives resistance [23]. Our findings underscore the potential of targeting the Wnt/β-catenin pathway alongside sorafenib to improve therapeutic outcomes. Specifically, we show that PRI-724, a potent inhibitor of β-catenin/CBP interactions, sensitizes HCC cells to sorafenib, overcoming resistance and significantly reducing tumor growth in vitro and in preclinical models.

We also address the limitations of current preclinical models, such as two-dimensional preclinical models and patient-derived xenografts [11–14], which do not fully replicate the complexity of human tumors. Our proposed MDT chip offers promising advancement in preclinical modeling. By incorporating patient-derived tumor tissues within a microfluidic system, this model preserves critical aspects of the tumor microenvironment, including cellular interactions and tissue architecture. Moreover, the MDT chip simulates in vivo conditions more accurately than traditional models do, providing a reliable platform for drug screening and efficacy testing, thereby enhancing the predictive accuracy of preclinical models and facilitating the identification of effective therapies and biomarkers for personalized treatment. Although the microfluidic 3D drug testing system demonstrated high consistency with in vivo outcomes, its capacity to fully replicate the complex tumor microenvironment and interactions may be limited. Further validation with additional preclinical models is necessary to confirm its predictive accuracy.

Currently, no validated biomarkers can reliably predict response to sorafenib [9]. Several studies have proposed p-ERK as a potential biomarker of sorafenib response, but results have been contradictory due to variations in detection methods, cohorts, and endpoints [40, 46]. In the BIOSTORM study, neither p-ERK, p-VEGFR2, nor VEGFA copy number status was significantly correlated with recurrence prevention by sorafenib [9], suggesting that the mechanisms of action of the drug and tumor biology in response to therapy are more complex than previously understood. Our findings indicated a strong correlation between β-catenin activity and sorafenib resistance based on the MDTs chip system and retrospective IHC analysis. β-catenin activation levels were an independent prognostic factor for patients with HCC treated with sorafenib, indicating that higher nuclear β-catenin levels may predict poor response to sorafenib treatment. However, further prospective studies with larger sample sizes are required to validate these results with independent cohorts and researchers.

Abnormal activation of the Wnt/β-catenin signaling pathway is a critical factor in regulating liver cancer malignant transformation [22]. Approximately half of patients with HCC exhibit abnormal activation of the Wnt pathway [21]. In most HCC cases, high β-catenin activity is associated with malignant transformation and sorafenib resistance [22, 23]. Our results support the concept that targeting the Wnt/β-catenin pathway may further enhance the susceptibility to TKI drugs, potentially reducing the required doses of traditional treatments.

Some researchers have proposed dividing HCC into two molecular subtypes, each accounting for approximately 50% of the disease: the proliferation class, driven by PI3K-AKT-mTOR, RAS-MAPK, and MET signaling, and the non-proliferation class, often associated with Wnt/β-catenin signaling pathway activation and immune exclusion, often due to *CTNNB1* mutations (encoding β-catenin) [8, 36, 37, 47, 48]. These two HCC classes indicate that current treatments, such as TKIs and immunotherapy, do not adequately cover diverse HCC populations or effectively stratify patients for benefits. TKIs such as sorafenib and regorafenib primarily target activated pathways in the proliferation class, likely benefiting from blocking cell proliferation by inhibiting the RAF/MEK/ERK signaling pathway and tumor angiogenesis by inhibiting the VEGF and platelet-derived growth factor receptors [8]. In contrast, non-proliferation class patients, marked by aberrant Wnt/β-catenin activation, may benefit less from these treatments. Furthermore, the crosstalk between these pathways facilitates the integration of various internal and external stimuli, resulting in unabated cancer cell proliferation as a net outcome [49]. This may support the association between aberrant Wnt/β-catenin activation and inherent sorafenib resistance in non-proliferation subtype or CTNNB1 class HCC. These findings highlight the clinical relevance and therapeutic potential of our combination strategy, which effectively targets both the proliferation and non-proliferation subtypes to improve response rates.

Studies on the crosstalk between the PI3K-AKT-mTOR, RAF/MEK/ERK, and Wnt/β-catenin pathways indicate that AKT and ERK can activate β-catenin through different regulatory mechanisms. AKT inhibits GSK3β by Ser9 phosphorylation, which in turn affects β-catenin phosphorylation at specific residues (Ser33, Ser37, and Thr41), leading to its ubiquitination and proteasomal degradation [36, 37]. Activated phosphorylated ERK translocates into the nucleus, activating transcription factors such as ATF, which induce target genes such as c-myc, c-fos, and c-Jun and regulate various cellular processes, including differentiation and transformation [35, 38]. This explains why, in our study, sorafenib alone, which targets p-AKT and p-ERK signaling, could partially suppress β-catenin activation. While PRI-724 directly inhibits β-catenin, sorafenib indirectly inhibits it through the RAS/RAF/MEK/ERK pathways. Moreover, the sorafenib and PRI-724 combination significantly inhibited AKT signaling; however, excessive PI3K inhibition with LY294002 paradoxically restored AKT and β-catenin activation. LY294002 binds to PI3K differently from quercetin by occupying part of the active site without extending into the phosphate-binding region [44], emphasizing the need for careful selection of AKT pathway inhibitors in clinical applications. Notably, *CTNNB1*-mutant MHCC-97H cells responded poorly to sorafenib alone, but when combined with PRI-724, β-catenin*, c-myc*, and *survivin* were significantly suppressed. These findings indicate that cells or patients with *CTNNB1* mutations may not respond to sorafenib because β-catenin activation is driven by the *CTNNB1* mutation rather than solely by p-AKT or p-ERK, making PRI-724 crucial for synergistically inhibiting β-catenin nuclear translocation and activation in HCC with *CTNNB1* mutation.

Through FDA-approved drug and inhibitor library screening, we identified PRI-724, a potent CBP/β-catenin transcription antagonist, which shows cytotoxicity in various cancer cell types [50, 51]. Our results showed that combining PRI-724 and sorafenib more effectively inhibited HCC in vitro, in vivo, and in MDT chip preclinical models, suggesting clinical potential. We used subcutaneous xenografts in immunodeficient mice to assess the antitumor efficacy of sorafenib and PRI-724; however, this model does not fully capture the complexity of tumor microenvironments, including organ-specific stroma, immune cell engagement, or angiogenic heterogeneity. Future studies should employ orthotopic or syngeneic models with intact immune systems to better evaluate tumor-stromal-immune crosstalk and validate therapeutic outcomes in a clinically relevant context. Importantly, PRI-724 has entered clinical trials for cancer (NCT01606579 and NCT01764477) and has demonstrated good safety, tolerability, and antifibrotic efficacy in patients with HCV-related cirrhosis [28, 52]. Although similar evidence in patients with cancer is lacking, we believe CBP/β-catenin antagonists offer new therapeutic avenues for liver cancer, especially when combined with other targeted and conventional therapies. However, further clinical studies are needed to validate these hypotheses.

In conclusion, our study employed the 3D drug testing system (MDT on chip) and RNA-seq to link sorafenib resistance in HCC to β-catenin activation, suggesting these patients may belong to the CTNNB1 subtype. Disruption of β-catenin increased the susceptibility of HCC cells to sorafenib by promoting apoptosis and suppressing cell growth, thereby enhancing our understanding of sorafenib resistance and introducing a novel preclinical model for drug testing accuracy. Through high-throughput screening of a large drug and inhibitor library, we identified PRI-724—a Wnt/β-catenin pathway inhibitor—as a promising candidate to overcome resistance. The combination therapy primarily enhances anti-tumor activity by inhibiting the Wnt/β-catenin/CBP/c-myc signal, β-catenin nuclear translocation, and the AKT/p-AKT and ERK/p-ERK signaling pathways, independent of *CTNNB1* mutation. The combination of sorafenib and PRI-724 significantly enhanced anti-tumor effects in vitro across various HCC cell lines, in vivo using xenograft models, and ex vivo with the MDT chip system to explore clinical applications (as shown in the figure of Graphical Abstract). Overall, this combination offers a promising therapeutic strategy to improve treatment responses in HCC patients.

## Materials and methods

### Patients and specimens

All specimens were obtained from the Tianjin Medical University Cancer Institute and Hospital (Tianjin, China). This study was approved by the Ethics Committee of Tianjin Medical University Cancer Institute and Hospital (Approval No. bc2021260). Informed consent was obtained from all participants. The study included two cohorts. Cohort 1 included 37 fresh liver cancer tissue samples obtained from patients who underwent surgical resection. These samples were used for 3D culture, drug sensitivity testing using the MDTs microfluidic chip system, and RNA-seq analysis. Cohort 2 included clinical data that were retrospectively collected regarding 119 patients with advanced HCC treated with sorafenib from 2009 to 2018. After screening for complete clinicopathological and drug response data, 41 patients with comprehensive clinical prognosis and drug response data were included. Patients were categorized into sorafenib-responsive and non-responsive groups based on a median overall survival (OS) of greater than or less than 10.7 months, respectively. The cutoff value of 10.7 months was determined from Phase III trial results for sorafenib (NCT00105443) [3]. Tissue samples from these 41 patients were analyzed for nuclear β-catenin levels using immunohistochemical staining.

### 3D drug testing microfluidic chip system for MDTs

The microfluidic chip assay was performed as described by Gervais et al. [19]. Each microfluidic platform consisted of two polydimethylsiloxane (PDMS) replicas created from micromachined master molds provided by Gervais et al. Biopsied tissue samples were cut into thin fragments (~1 mm) using a scalpel and immersed in Hank’s balanced saline solution (HBSS) supplemented with 10% fetal bovine serum (FBS, PAN–Seratech, Aidenbach, Germany) and 1% penicillin/streptomycin (Hyclone, Logan, UT, USA). The tissue fragments were then shaped into spherical MDTs using a 500 μm diameter tissue punch (Zivic Instruments, Pittsburgh, PA, USA) and maintained in HBSS with antibiotics but without serum until loaded into the microfluidic channels.

Five MDTs were loaded into each channel using a P20 micropipette, where they settled at the bottom of the channel. Fluid flow was induced by aspirating from the device outlet with the same micropipette. As the MDTs traveled through the channels, the flow was suspended for 1–2 s, allowing each MDT to settle into its desired well. This process was repeated until all five fluidic channels were fully loaded, resulting in 25 MDTs per device. After loading, the HBSS was replaced with Dulbecco’s modified Eagle medium (DMEM) (Corning, NY, USA) supplemented with 10% FBS and 1% penicillin/streptomycin. The MDTs were treated with or without 10 μM sorafenib (Selleck, Houston, TX, USA), 10 μM PRI-724 (Selleck), or a combination of both for 4 or 7 days. Each treatment group, including the control group, consisted of at least 30 MDTs, with every 5 MDTs forming an independent channel, resulting in six replicates per group. Following drug treatment, MDTs were isolated and stained for off-chip and on-chip analyses to assess their response to the various drugs.

### MDTs On-chip analysis

For on-chip analysis, dual-fluorescent staining was performed at the endpoint after drug treatment using CellTracker™ Green CMFDA (CTG, Thermo Fisher Scientific, Waltham, MA, USA) and PI (BD Biosciences, San Jose, CA, USA). CTG was used to label viable cells, while PI marked the nucleic acids of dead cells. HBSS solutions containing CTG (5 μM) alone or a combination of CTG (5 μM) and PI (1.5 μM) were sequentially added to the samples. The MDTs were incubated with CTG for 1 h, followed by incubation with both dyes for 30 min. After staining, the dye solutions were replaced with HBSS solution, and the samples were imaged under a microscope (Zeiss Imager Z2, China).

### Off-chip analysis

For off-chip analysis, the two PDMS replicas of the microfluidic device were carefully separated, and the MDTs were collected into 1.5 mL EP tubes, each tube containing 15 MDTs, at the treatment endpoint. The collected MDTs were then digested into single-cell suspensions using mouse or human Tumor Dissociation Kit (MACS, Miltenyi Biotec, Bergisch Gladbach, Germany) for 30 min at 37 °C. Following the instructions of the PE Annexin V Apoptosis Detection Kit I (BD Biosciences), the cells were stained with PE-Annexin V and 7-AAD for 15 min at room temperature. The apoptosis rates and tissue responses to the drug were analyzed by flow cytometry.

### Cell culture

Human HCC cell lines HepG2 and SNU387 were acquired from the American Type Culture Collection (ATCC, Manassas, VA, USA), while human HCC cell lines PLC/PRF/5, Huh7, Hep3B, MHCC-97L, MHCC-97H, and MHCC-LM3 were purchased from Wuhan Procell Life Science and Technology Co., Ltd (Wuhan, China). HLE cells were acquired from the Health Science Research Resources Bank (Osaka, Japan) and the National Collection of Authenticated Cell Cultures (Shanghai, China). PLC/PRF/5, Huh7, MHCC-97L, MHCC-97H, MHCC-LM3, and HLE cells were routinely cultured in DMEM supplemented with 1% penicillin/streptomycin and 10% FBS in a humidified incubator at 37 °C with 5% CO_2_. Hep3B and HepG2 were cultured in Minimum Essential Medium (Corning) with 1% penicillin/streptomycin and 10% FBS in a humidified 5% CO_2_ incubator at 37 °C. SNU387 cells were cultured in RPMI-1640 medium (Corning) plus 1% penicillin/streptomycin and 10% FBS in a humidified incubator at 37 °C with 5% CO_2_.

### Cell transfection assay

The transfection assay was performed as described previously [3]. In brief, lentiviral particles were generated by co-transfecting HEK293T cells with packaging plasmids (pVSVG and psPAX2) along with the expression plasmids encoding shRNA sequences targeting either shRNA against β-catenin or scramble control (SCR) using PEI (Polysciences, Warrington, USA). After 48 h of incubation, lentiviral particles were harvested and filtered (0.45 µm). Subsequently, the Hep3B, HepG2, and MHCC-97H cells were used for transfection. Post-transduction, cells were allowed to recover for 24 h in a fresh medium before initiating selection. Stable cells were selected by adding puromycin (Gibco) to the culture medium for 5–7 days, after which single-cell clones or stable pools were expanded. The cells were passaged at least three times to ensure stable integration of the transgenes. For experimental use, cells were generally utilized between passages 5 and 8 after transduction to ensure stability of the expression system. The plasmid map and vector components include the U6 promoter-driven shRNA cassette, reporter elements, and puromycin-resistance marker. The shRNA target sequences for β-catenin knockdown were shown as follows:

shRNA-oligo1: 5′-ccggTTGTTATCAGAGGACTAAATActcgagTATTTAGTCCTCTGATAACAAttttt-3′,

shRNA-oligo2: 5′-aattaaaaaTTGTTATCAGAGGACTAAATActcgagTATTTAGTCCTCTGATAACAA-3′.

### Drugs and treatment

Sorafenib and PRI-724 (Selleck, Houston, TX, USA) were prepared for treatment in vitro and in vivo. To avoid excessive drug toxicity, we first determined the IC50 values of sorafenib and PRI-724 in different HCC cell lines. For combination treatment experiments in HCC cells, we applied a fixed IC50-based ratio of the two drugs (usually using a two-fold serial dilution with several concentration points above and below its IC50 value); it will always remained at a constant ratio [at the (IC50)_1_/(IC50)_2_ ratio, according the Chou-Talalay method for drug synergy analysis] [53]. For MDTs derived from human tumor samples, we adopted drug concentrations as those used for the relatively sorafenib-resistant MHCC-97H cells (sorafenib = 5 μM, PRI-724 = 5 μM), ensuring consistency. Moreover, the clinical peak plasma concentration (Cmax) of sorafenib was between 5 and 7 mg/L, which is 7.8–10.9 μM [54], while the maximum plasma concentration of PRI-724 in humans is 11.4 ± 1.8 ng/mL at doses of 10 mg/m²/day [52], corresponds to approximately 17.3 µM. Therefore, the selected concentrations in human samples and HCC cells remain well within the clinically relevant range, ensuring the translational potential and applicability of this combination therapy.

### Tumor xenograft model

NOD scid gamma (NSG) mice (5–6 weeks old, male) were obtained from Gempharmatech (Jiangsu, China) and housed in the SPF facility at Tianjin Medical University Cancer Institute and Hospital. The HCC cell lines MHCC-97H, HLE, and Hep3B (5 × 10^6^ cells) were subcutaneously inoculated into the armpits of the NSG mice. Once tumor volume reached ~100 mm^3^, the mice were divided into control and treatment groups (*n* = 5). The control group received normal saline; the sorafenib group was administered 30 mg/kg sorafenib by daily gavage; the PRI-724 group received 50 mg/kg PRI-724 via daily intraperitoneal injection; and the combined sorafenib and PRI-724 group received both sorafenib (30 mg/kg) and PRI-724 (50 mg/kg) daily. Tumor size and body weight were monitored every 2 days using Vernier calipers. Tumor volume (mm^3^) was calculated as length × width^2^ × 0.5. After 14 days of treatment, the mice were sacrificed, and the tumors were dissected, weighed, and frozen for subsequent MDT preparation, immunofluorescence, and immunohistochemical evaluation. All animal experiments were approved by the Ethics Committee of Tianjin Medical University Cancer Institute and Hospital (No. PMIF-2021077).

### RNA-seq and single-sample gene set enrichment analysis (ssGSEA) analysis

Total RNA from 37 HCC tumor tissues and eight para-cancer tissues was used as the starting material for RNA sample preparations. Sequencing libraries were created using the NEBNext Ultra Directional RNA Library Prep Kit for Illumina (NEB E7420, NEB, Ipswich, MA, USA), according to the manufacturer’s instructions. Briefly, regulatory ncRNAs and mRNA were extracted from the total RNA using probes to eliminate rRNA. Fragmentation was induced with divalent cations at elevated temperatures in the first-strand synthesis reaction buffer (5×). The first-strand cDNA synthesis was performed using random hexamer primers and M-MuLV Reverse Transcriptase (RNaseH). Second-strand cDNA was synthesized using DNA Polymerase I and RNase H, followed by conversion of the remaining overhangs into blunt ends via exonuclease/polymerase activities. After adenylation of the 3′ ends of DNA fragments, the NEBNext Adaptor with a hairpin loop structure was ligated for hybridization. To select cDNA fragments of ~370–420 bp, the library fragments were purified using the AMPure XP system (Beckman Coulter, Brea, CA, USA. Next, 3 µL USER Enzyme was added to the size-selected, adaptor-ligated cDNA at 37 °C for 15 min, followed by 5 min at 95 °C before polymerase chain reaction (PCR). PCR was then conducted using Phusion High-Fidelity DNA polymerase, Universal PCR primers, and an Index Primer. Finally, the PCR products were purified, and library quality was assessed using the Agilent 5400 (Agilent, Santa Clara, CA, USA) system and quantified by qPCR. The qualified libraries were pooled and sequenced on Illumina platforms using the PE150 strategy at Novogene Bioinformatics Technology Co., Ltd (Beijing, China) based on the required effective library concentration and data amount. The RNA-Seq data used in this study can be obtained from the corresponding author upon request. Genes with|log2 (Fold Change)|≥ 1 and an adjusted *p* value ≤ 0.05 were filtered as DEGs. To evaluate the function of DEGs associated with Wnt pathway activity, we used ssGSEA (run by the R package “GSVA”) to assess the activity levels of gene sets related to “liver-related Wnt” and “classical Wnt” genes signaling each sample.

### Hematoxylin and eosin (H&E) and IHC staining

H&E staining was performed to assess the necrotic area of mouse xenograft tissues. Paraffin-embedded tumor sections were processed with xylene and ethanol, stained with H&E, dehydrated, sealed, mounted, dried, and examined. IHC staining was used to evaluate the protein levels of nuclear β-catenin in paraffin-embedded samples, following previously described methods [55]. The primary antibody used for IHC staining was anti-β-catenin (1:500) (Abcam, Cambridge, England). Histological sections were scanned using a light microscope (Olympus, Tokyo, Japan), and the IHC scoring criteria were as follows: staining intensity was graded into four categories (0 = no staining, 1 = weak staining, 2 = moderate to strong staining, and 3 = strong staining). The percentage of positively stained cells was categorized into four levels (0 = 0% positive cells, 1 = 1–10% positive cells, 2 = 10–20% positive cells, 3 = 20–60% positive cells, and 4 = 60–100% positive cells). The final IHC score for nuclear β-catenin was calculated by multiplying the staining intensity score by the percentage score. Final IHC scores greater than 0 were defined as positive expression, while scores of 0 were defined as negative expression.

### Western blotting

Western blotting was applied to assess protein levels in cell lysates following previously described methods [56]. Briefly, equal amounts of cell lysates were subjected to 8–12% sodium dodecyl sulfate-polyacrylamide gel electrophoresis and transferred to polyvinylidene fluoride membranes (Immobilon-P, Millipore, Billerica, MA, USA). Nuclear and cytosolic fractions were extracted according to the instructions for the NE-PER nuclear and cytoplasmic extraction reagents (Thermo Scientific). Membranes were blocked with 5% skim milk or bovine serum albumin for 1 h at room temperature and immunoblotted with diluted primary antibodies overnight at 4 °C. The primary antibodies were used against the following targets: β-catenin (1:1000, Abcam), active β-catenin (1:2000, Millipore), c-myc (1:200), and glyceraldehyde 3-phosphate dehydrogenase (GAPDH; 1:1000) from Santa Cruz Biotechnology (Santa Cruz, CA, USA); ERK (1:1000), p-ERK (1:4000), AKT (1:1000), p-AKT (1:1000), P38 (1:1000), and p-P38 (1:1000) from Cell Signaling Technology (Beverly, MA, USA). After incubation with peroxidase-conjugated secondary antibodies (Santa Cruz Biotechnology) for 1 h at room temperature, the immunoblots were detected using electrochemiluminescence.

### Cell viability assay

Lentiviral particles were produced by transfecting HEK293T cells with packaging plasmids (VSVG and ΔR) and expression plasmids containing either shRNA targeting β-catenin (KD) or a matched scramble control (SCR) using Lipofectamine 3000 (Invitrogen, Waltham, MA, USA). The cells were then infected with these lentiviruses to create stable sh-β-catenin or SCR cell lines. Puromycin (Gibco, Billings, MT, USA) was used to select the stably transfected cells.

### Cell viability assay

HCC cell lines were cultured in the presence of a vehicle (0.1% dimethyl sulfoxide) or increasing drug concentrations. Cell viability was assessed using the Cell Counting Kit-8 (CCK-8) (Biosharp Life Sciences, Hefei, China) following the manufacturer’s instructions. In brief, 2 × 10^3^ cells were seeded in 100 μL of cell medium per well in a 96-well plate. After treatment with the specified reagents, 10 μL of CCK-8 solution was added to each well and incubated at 37 °C for 2 h. Optical density (OD) was measured at 450 nm using a microplate reader. The combination effect and potential synergism were evaluated through quantitative analysis of the dose-effect relationship, employing the Chou and Talalay method [53]. Each experiment calculated a combination index (CI) using CalcuSyn software from Biosoft (Cambridge, UK). According to this method, Log CI values between −0.046 and 0.041 indicate an additive effect, values from −0.52 to −0.046 suggest synergy, values less than −0.52 denotes strong synergy, while values of 0.041 are considered antagonistic.

### Clonogenic cell survival assay

Cells were seeded at a density of 1 × 10^3^ cells/well in 12-well plates or 500 cells/well in 24-well plates. After overnight incubation, the cells were treated with varying concentrations of sorafenib and/or PRI-724, followed by 7–14 days of incubation. The colonies were washed with PBS, fixed in 4% paraformaldehyde (Servicebio, Wuhan, China) for 15 min at room temperature, and stained with crystal violet (Beyotime, Beijing, China). The crystal violet-stained colonies were then resolved using methanol, and absorbance was measured at 540 nm.

### Fully automated screening of the FDA-approved compound library

A library of 1463 FDA-approved drugs and small-molecule inhibitors was purchased from Selleck, provided in 100-μL aliquots as 5-mM DMSO stock solutions. Detailed information about the drug and inhibitor library is available in Supplementary Table 1. High-throughput screening of the compound library was performed using a fully automated Perkin Elmer G3 integrated system (Perkin Elmer, Shelton, CT, USA). MHCC-97H cells were seeded into 384-well plates (CellCarrier-384 plates, Perkin Elmer) at 50 μL per well with an automatic liquid handler. After seeding, the plates were incubated at 37 °C for 24 h. The following day, compounds were added to the plates at 10 μM, with or without 10 μM sorafenib. Four treatment groups were established: DMSO control, sorafenib alone, drug candidates alone, and combination treatment. DMSO served as the negative control. After 72 h of incubation, cell viability was assessed using the CCK-8 assay. 5 μL CCK-8 reagent (Biosharp Life Sciences) was added to each well and co-incubated with the cells at 37 °C for 3 h. The OD value was measured at 450 nm using a BioTek plate reader (Agilent).

### Immunofluorescence (IF) staining

Immunofluorescence analysis was performed as described previously [55]. Sterile coverslips were placed in 12-well plates received 4 × 10^4^ cells/well. After 24 h of incubation at 37 °C with 5% CO_2_, cells were fixed, permeabilized, and blocked. Primary antibodies against active β-catenin (1:200, Millipore) were applied overnight at 4 °C, followed by staining with Alexa Fluor 594 and counterstaining with ProLong Gold (Invitrogen). Imaging was conducted with a confocal microscope (Zeiss LSM880; Carl Zeiss AG, Oberkochen, Germany).

### qRT-PCR assay

Total RNA was extracted from cells or tumor tissues using the TRlzol reagent (Invitrogen), following the manufacturer’s instructions. RNA concentration was quantified by measuring absorbance at 260 and 280 nm using a NanoDrop spectrophotometer (NanoDrop Technologies, Wilmington, DE, USA). Subsequently, 1 µg of total RNA was reverse-transcribed into cDNA using PrimeScript^TM^ RT Master Mix (TaKaRa, Tokyo, Japan). For qRT-PCR, cDNA samples were amplified in biological triplicate using the AceQ SYBR qPCR Master Mix (Vazyme, Nanjing, China) on a LightCycler 480 II system (Roche, Basel, Switzerland) according to the provided instructions. Gene expression levels were normalized to GAPDH expression. Specific PCR primer sequences are listed in Supplementary Table 2.

### TUNEL staining

Tumor sections were fixed in formalin and made into paraffin sections. DeadEnd™ Fluorometric TUNEL System (Promega, Madison, WI, USA) was used according to the provided instructions.

### Statistical analysis

All data are presented as means ± standard error of the mean from at least three independent biological replicate experiments. Statistical analyses were conducted using GraphPad Prism 9.0 (GraphPad Software, Boston, MA, USA) with analysis of variance or two-tailed Student’s t-test, as appropriate. Survival analyses were performed using the Kaplan–Meier method, and survival curves were compared using the log-rank test. Variables statistically significant in the univariate analyses were included in a multivariable Cox proportional hazards model to calculate hazard ratios (HR) and 95% confidence intervals (CI). The significance levels are as follows: *p < 0.05, **p < 0.01, ***p < 0.001, ****p < 0.0001, and n.s. not significant.

## Supplementary information

supplementary material

Full and uncropped western blots - source data

## Data Availability

All the original data presented in this study are included in the article and supplementary material. Any further requests can be directed to the corresponding authors.
