# Supplementary material for: Inhibition of Wnt/β-catenin increases anti-tumor activity by synergizing with sorafenib in hepatocellular carcinoma
Source: Cell Death Dis. 2025 Jul 1;16(1):466. doi: 10.1038/s41419-025-07789-5 (PMC12216529; doi:10.1038/s41419-025-07789-5)
Supplement: Supplementary file 1 — supplementary material [file 41419_2025_7789_MOESM1_ESM.pdf]

Supporting information

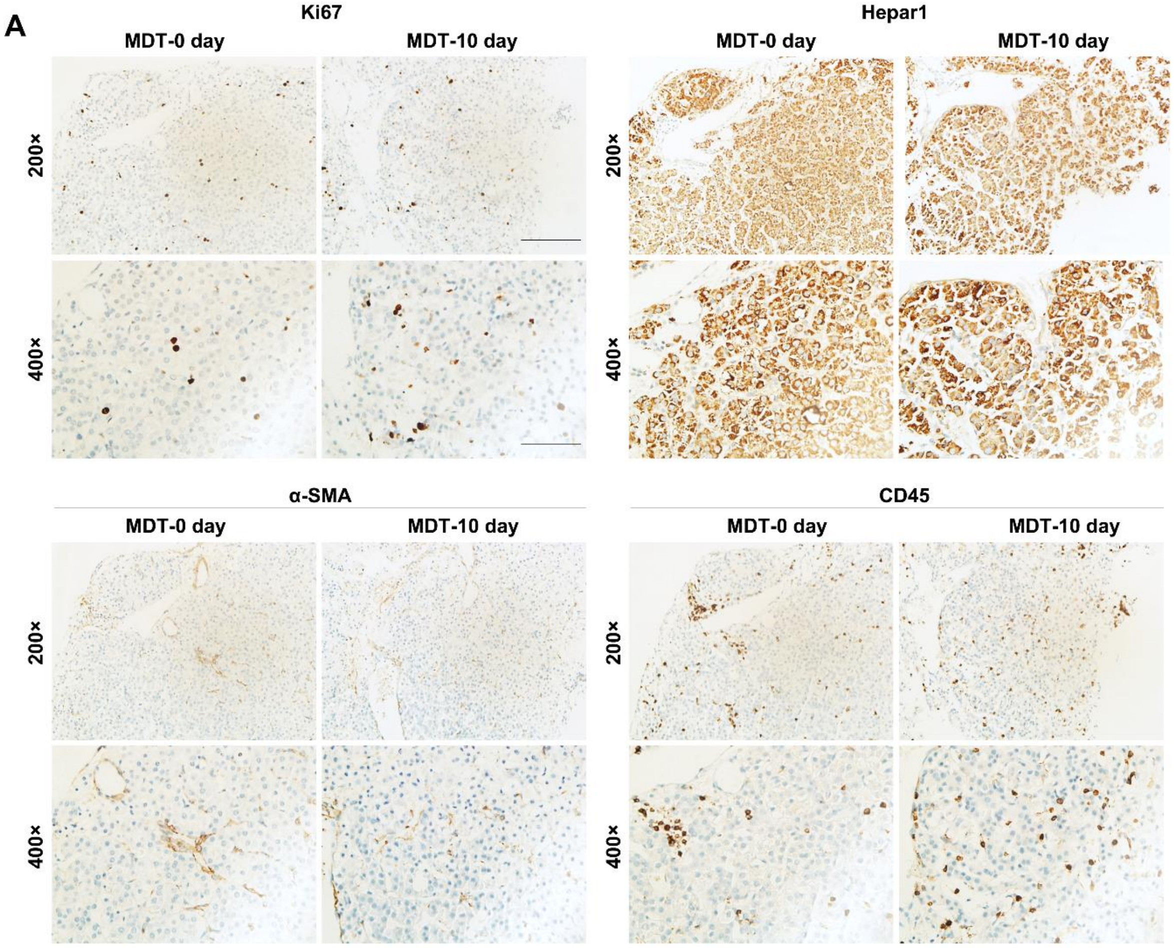

Supplementary Figure 1 Immunohistochemical analysis of cellular composition in MDTs. A). Ki67, Hepar1, α-SMA and CD45 staining in MDTs at day 0 and day 10. scale bars, 200 μm for 200 ×; 100 μm for 400 ×.

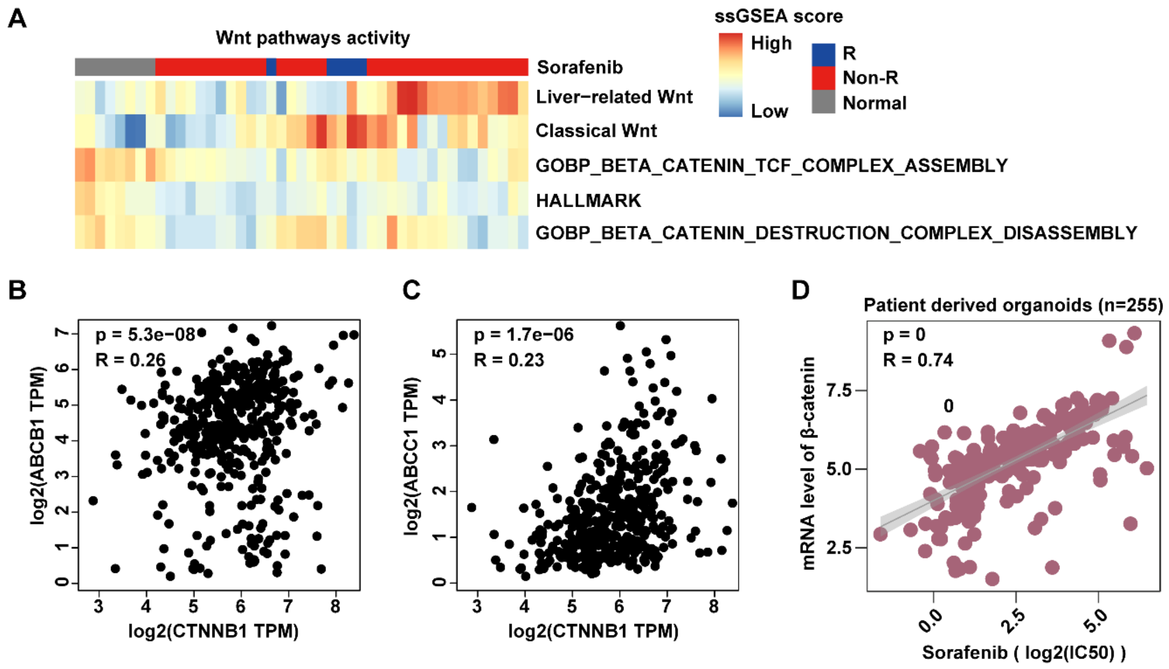

Supplementary Figure 2 Wnt/β-catenin signal is associated with sorafenib resistant in HCC. A&B) Analysis of GEPIA datasets to further confirm the correlation between β-catenin expression and resistance linked markers including ABCB1 and ABCC1. C) The correlation analysis between β-catein level and sorafenib IC50 in 255 organoids from HCC patients.

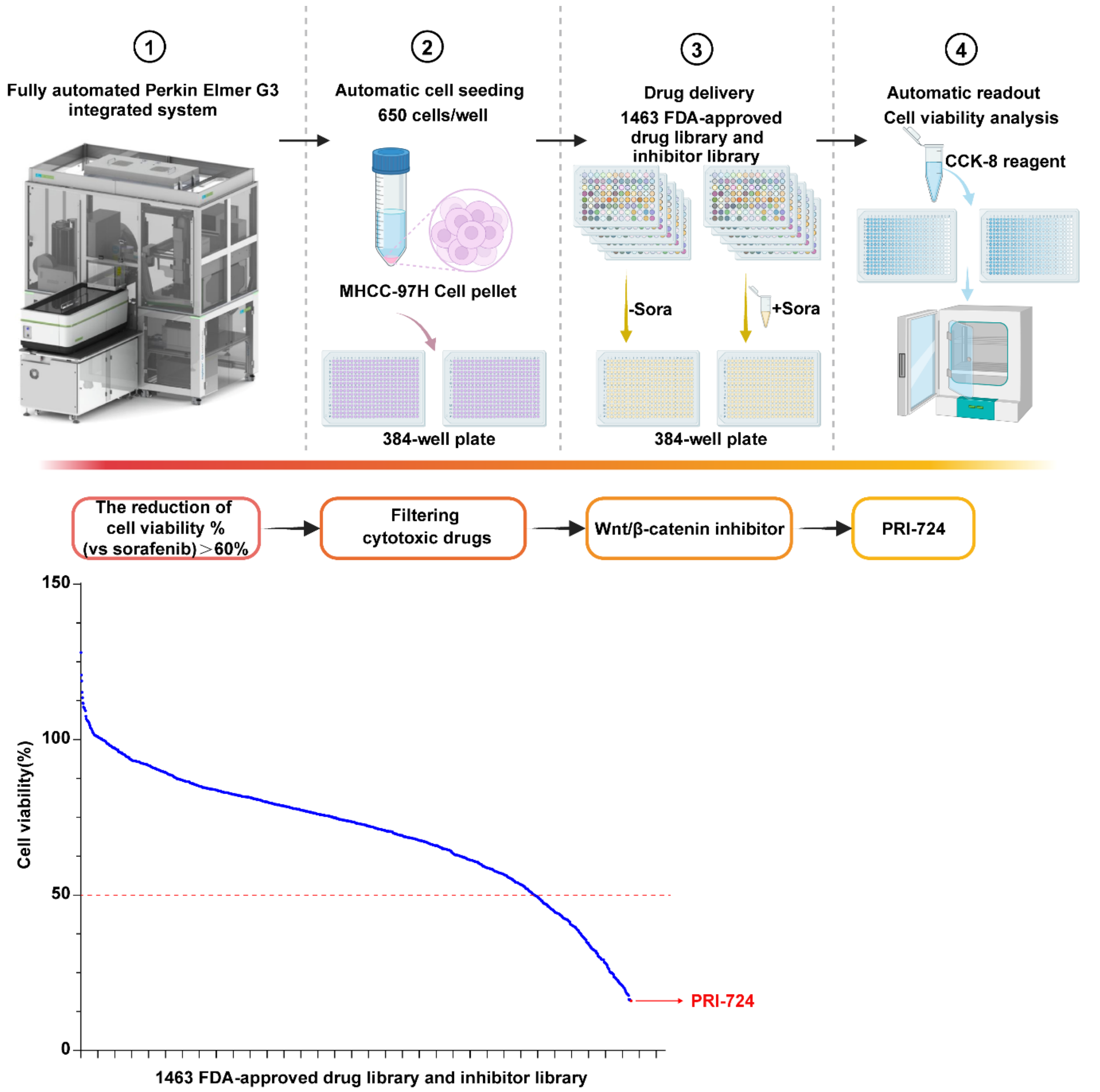

Supplementary Figure 3 Up, screening diagram showing the procedure for screening drugs in FDA-approved drug library that can enhance the sensitivity of sorafenib. Down, statistical distribution curve of cell viability in HCC cells of 1463 FDA-approved drugs.

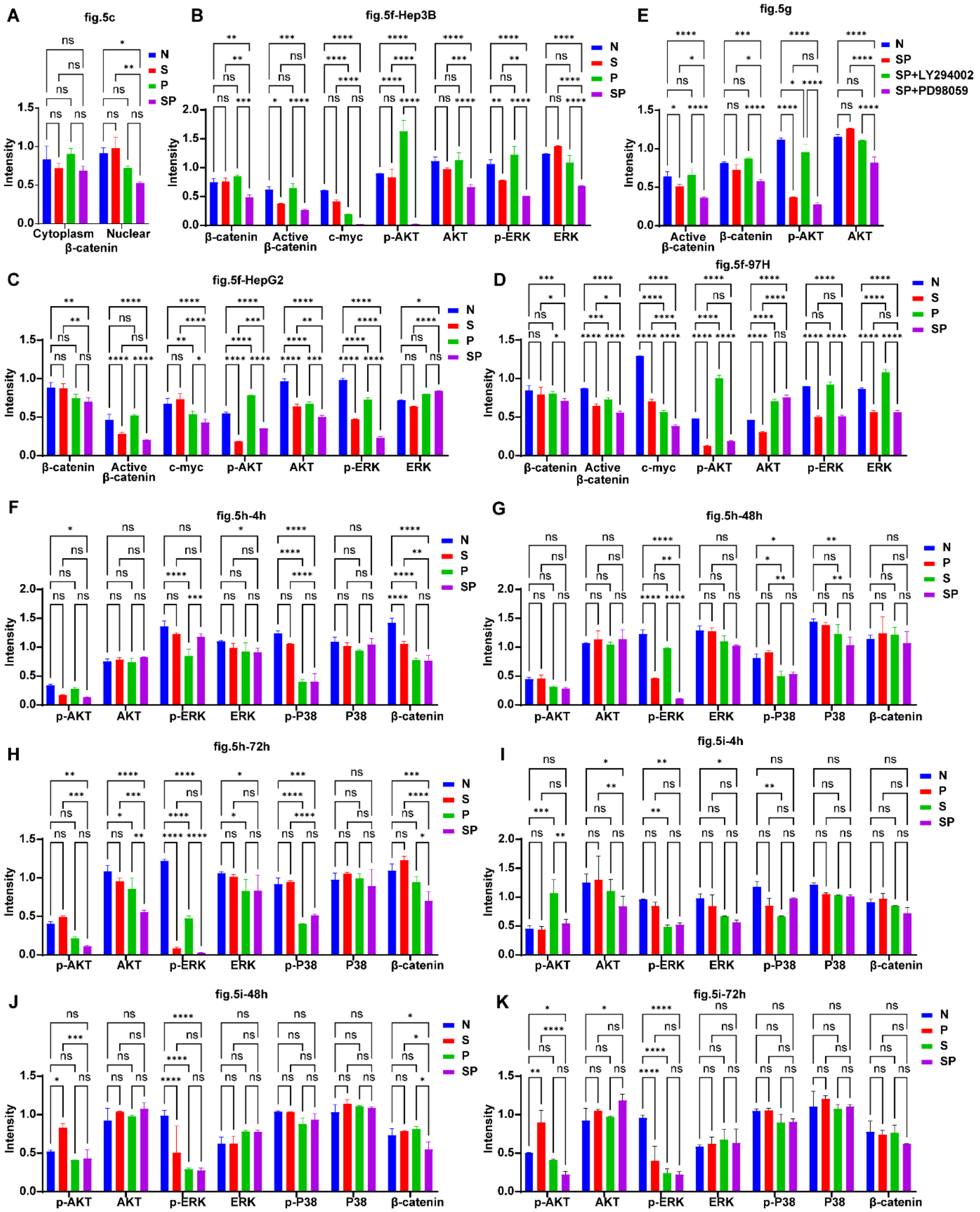

Supplementary Figure 4 The quantified intensities of Western blot bands from Figure 5. A) Cytoplasmic and nuclear distribution of β-catenin in MHCC-97H cells, and the quantified intensities of from Figure 5C. B–D). Relative expression of total and active β-catenin, c-myc, p-AKT, AKT, p-ERK, and ERK in Hep3B, HepG2, and MHCC-97H cells, respectively, and the quantified intensities of from Figure 5F. E) Effects of PI3K inhibitor (LY294002) or ERK inhibitor (PD98059) on β-catenin pathway activity in MHCC-97H cells, and the quantified intensities of from Figure 5G. F–K) Time-course

analysis (4 h, 48 h, 72 h) of signaling pathway proteins (AKT, ERK, P38, β-catenin) in Hep3B and MHCC-97H cells treated with vehicle (N), sorafenib (S), PRI-724 (P), or combination (SP), and the quantified intensities of from Figure 5H and 5I.

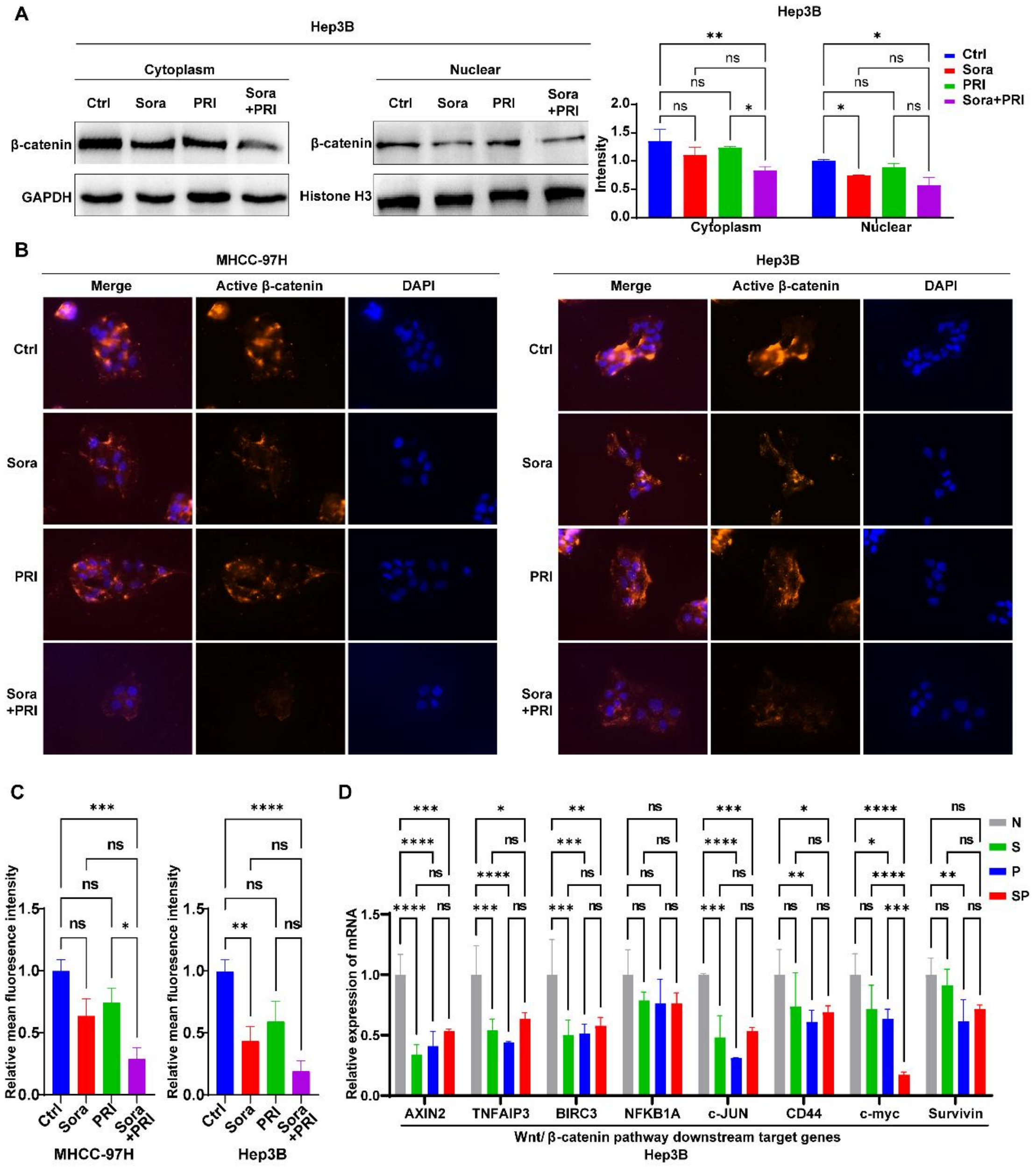

Supplementary Figure 5 Validation of β-catenin pathway inhibition by combination treatment in Hep3B and MHCC-97H cells. A) Western blot analysis of cytoplasmic and nuclear β-catenin in Hep3B cells treated with vehicle (Ctrl), sorafenib, 1.9 μM (Sora), PRI-724, 2.5 μM (PRI), or their combination (Sora+PRI) for 24 h. GAPDH and Histone H3 were used as loading controls for cytoplasmic and nuclear fractions, respectively. Densitometric quantification is shown in the right panel (mean ± SD, n = 3). B) Immunofluorescence staining of active β-catenin (orange) and nuclei (DAPI, blue) in MHCC-97H and Hep3B cells under the indicated treatment conditions. Scale bar = 10 μm. C) Quantification of mean fluorescence intensity for active β-catenin in MHCC-97H and Hep3B cells (n = 3 independent fields per group). D) qRT-PCR analysis of canonical Wnt/β-catenin downstream target genes in Hep3B cells treated as in (A).

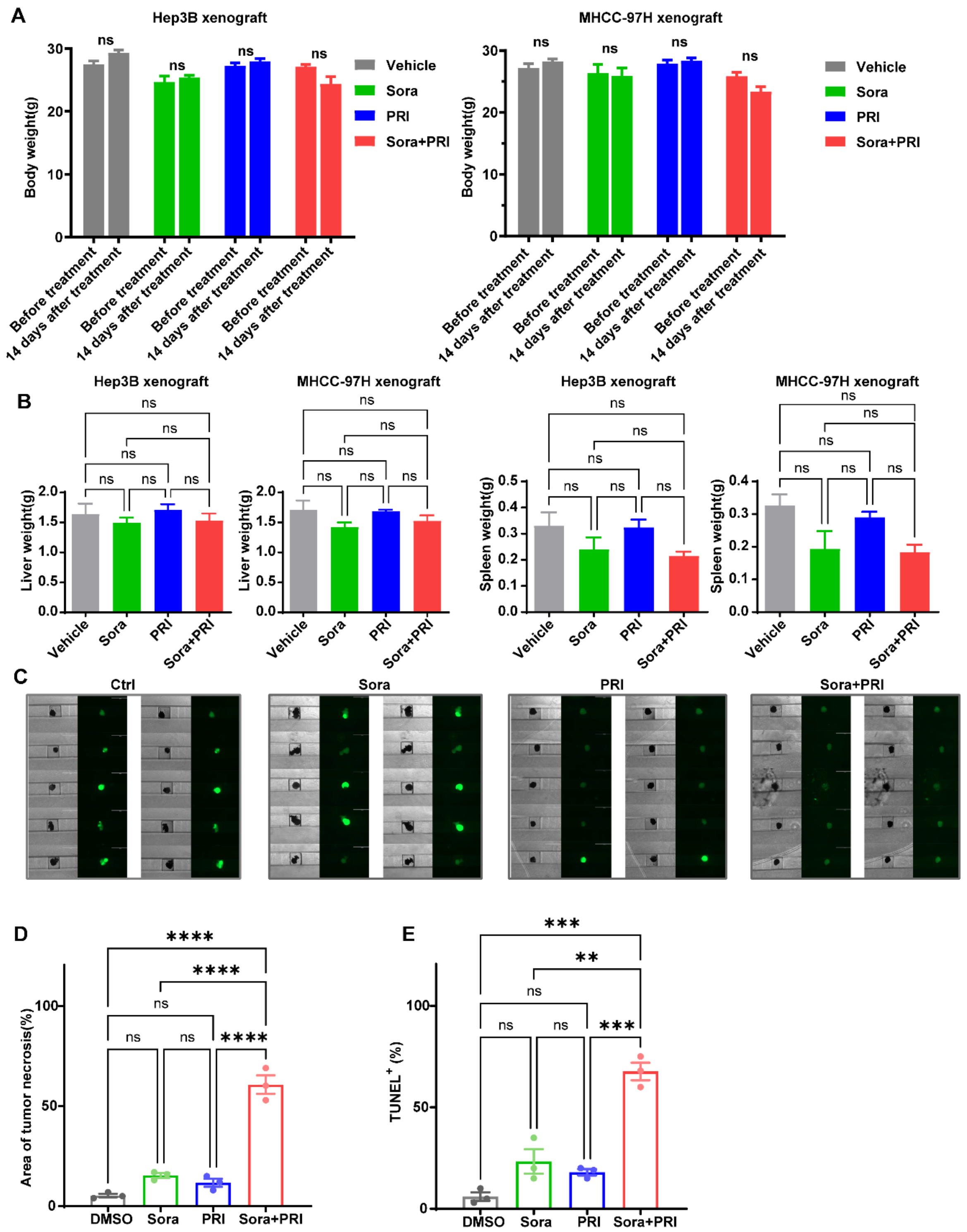

Supplementary Figure 6 The safety and antitumor activity of combination treatment with sorafenib and PRI-724. A) The mice body weight before treatment and after 14 days treatment with sorafenib, PRI-724 and their combination. B) The liver and spleen weight after different treatments in the same batch of experiments. C) The viability of MDTs from Hep3B xenograft tumor tissues on chip by CTG staining after treatment with PRI-724 (3.8 μM) and sorafenib (5 μM) for 7days. D&E) Percentage of tumor necrotic area (D) and TUNEL^{+} apoptosis area (E) in 4 indicated treatment groups based on H&E staining of MHCC-97H xenograft.

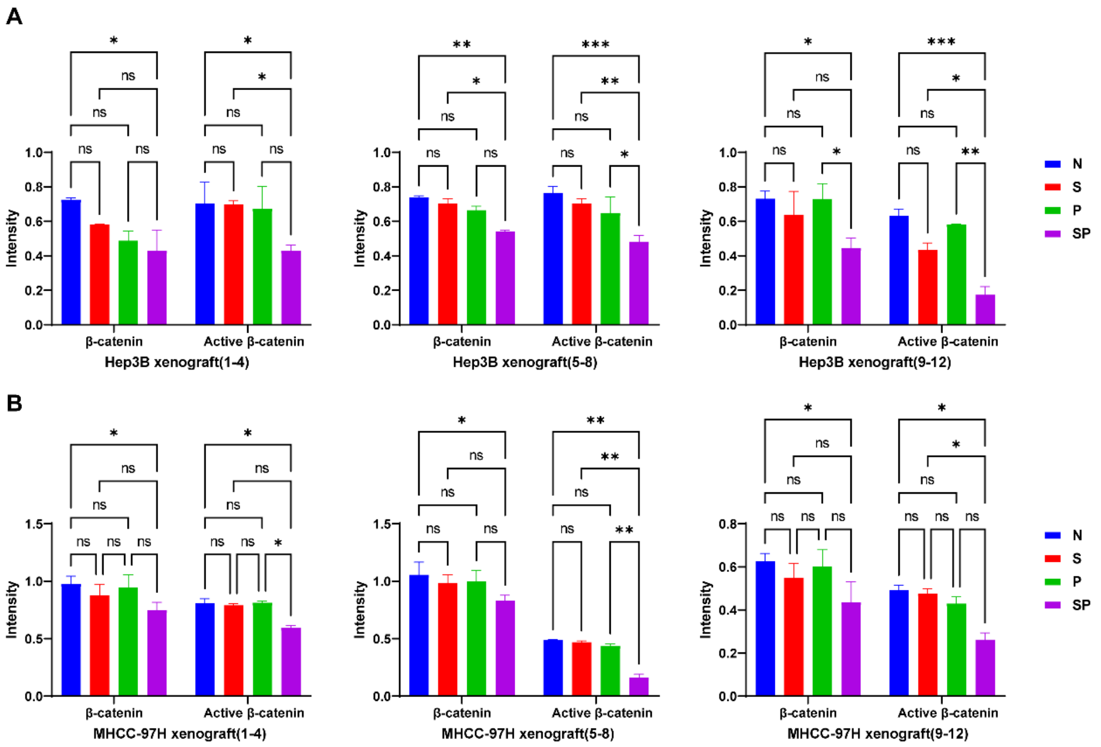

Supplementary Figure 7 The quantified intensities of Western blot bands from Figure 6I and 6J.

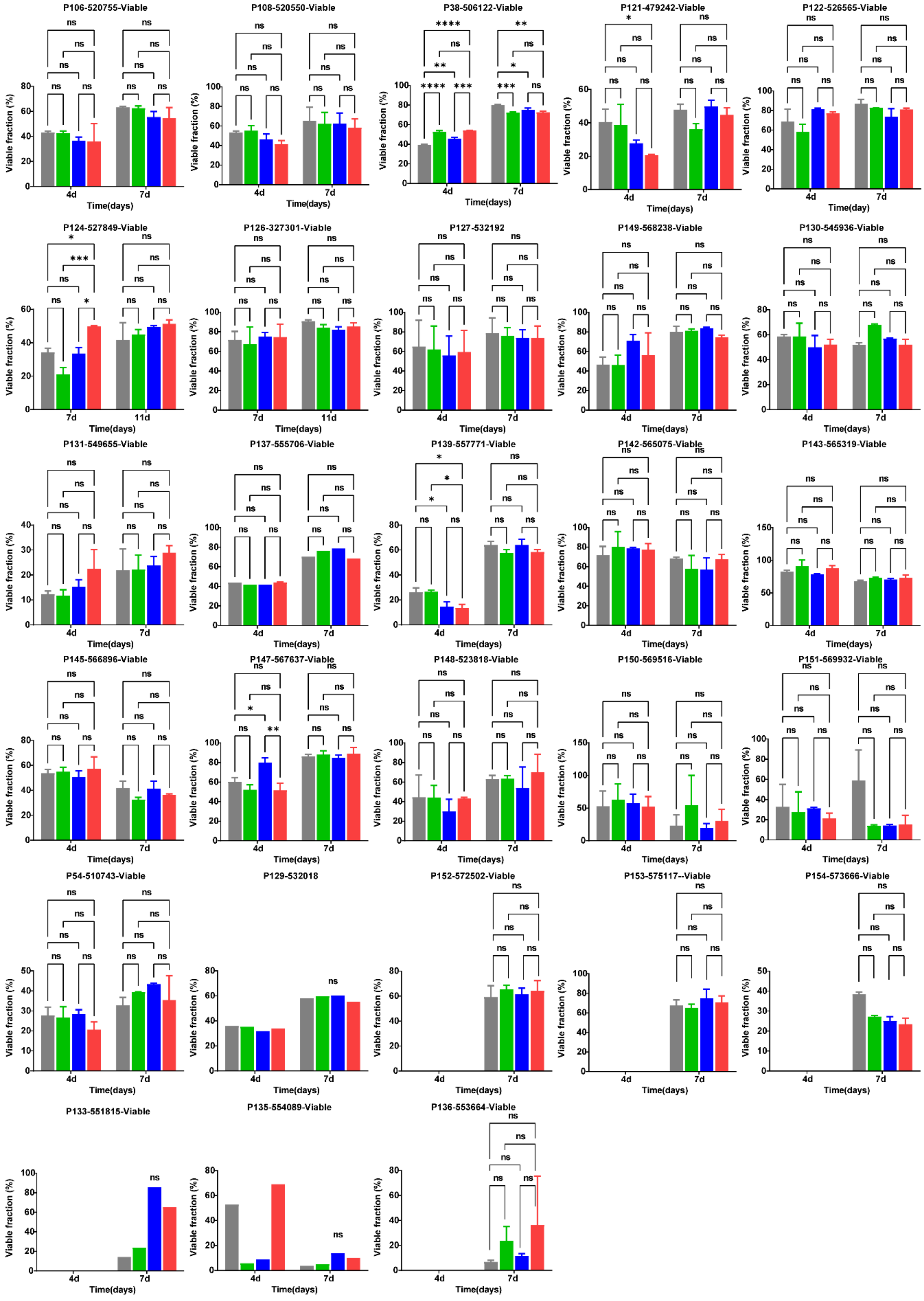

Supplementary Figure 8 The MDT samples from 37 HCC patients were subjected to 3D culture and drug sensitivity testing using the MDTs chip system treated with PRI-724 (5 μM) and sorafenib (5 μM)

for 4 and 7 days. The results for the 28 cases that not responded to single, or combination drug treatments are shown by the apoptosis rates analysis.

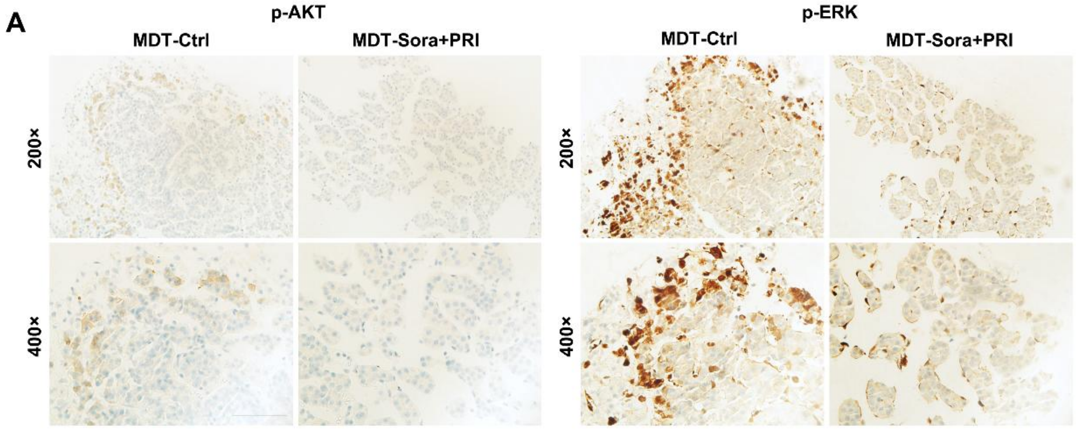

Supplementary Figure 9 Immunohistochemical analysis of p-ERK and p-AKT in MDTs. A). The comparison of p-ERK and p-AKT staining in MDTs between combination treatment and control groups. scale bars, 200 µm for 200 ×; 100 µm for 400 ×.

Supplementary Table 1. The list of 1463 FDA-approved compound library.

No.	Cat No.	Drug name
1	S8968	PRI-724
2	S1393	Pirarubicin
3	S2481	Manidipine
4	S1302	Ifosfamide
5	S1042	Sunitinib Malate
6	S1787	Teniposide
7	S2456	Chlorpromazine HCl
8	S1351	Ivermectin
9	S1432	Sumatriptan Succinate
10	S2031	Fenticonazole Nitrate
11	S2382	Evodiamine
12	S4631	Prochlorperazine dimaleate salt
13	S3611	(+)-Fangchinoline
14	S1305	Mercaptopurine (6-MP)
15	S2098	Bexarotene
16	S1053	Entinostat (MS-275)
17	S4107	Clofazimine
18	S1550	Pimobendan
19	S1640	Albendazole
20	S2077	Atorvastatin Calcium
21	S1207	Tivozanib (AV-951)
22	S4589	Amodiaquine dihydrochloride dihydrate
23	S1485	HMN-214
24	S1771	Chlorprothixene
25	S3698	Nortriptyline hydrochloride
26	S1178	Regorafenib (BAY 73-4506)
27	S1453	Tipifarnib
28	S1831	Carvedilol
29	S1526	Quizartinib (AC220)
30	S1390	Ondansetron HCl
31	S1144	Ivacaftor (VX-770)
32	S1189	Aprepitant
33	S2517	Maprotiline HCl
34	S4282	Nelfinavir Mesylate
35	S1241	Vincristine sulfate
36	S2284	Colchicine
37	S4505	Vinblastine sulfate
38	S4269	Vinorelbine Tartrate
39	S4296	Salmeterol Xinafoate
40	S1792	Simvastatin
41	S1257	Posaconazole
42	S4084	Lomerizine 2HCl
43	S3019	Ciclopirox ethanolamine
44	S2528	Ciclopirox
45	S1776	Toremifene Citrate
46	S2028	Diclazuril
47	S1848	Curcumin
48	S4783	Benzyl isothiocyanate
49	S4541	Triclosan
50	S4619	Itopride hydrochloride

51	S4223	Mevastatin
52	S3030	Niclosamide
53	S4406	Pindolol
54	S2075	Rosiglitazone HCl
55	S2217	Irinotecan HCl Trihydrate
56	S3676	Carbendazim
57	S3022	Cabazitaxel
58	S2334	Oleanolic Acid
59	S1333	Fluoxetine HCl
60	S4000	Pergolide Mesylate
61	S2625	Fostamatinib (R788)
62	S4182	Nifuroxazide
63	S1120	Everolimus (RAD001)
64	S3689	Ethidium bromide
65	S2475	Imatinib (STI571)
66	S1190	Bicalutamide
67	S3671	Quinestrol
68	S2814	Alpelisib (BYL719)
69	S1721	Azathioprine
70	S2151	Sonidegib (Erismodegib, NVP-LDE225)
71	S1979	Amiodarone HCl
72	S3060	Medetomidine HCl
73	S2290	Dihydroartemisinin (DHA)
74	S1830	Oxfendazole
75	S4597	Lercanidipine hydrochloride
76	S1021	Dasatinib
77	S3047	Otilonium Bromide
78	S4394	Clofoctol
79	S2680	Ibrutinib (PCI-32765)
80	S2017	Benidipine HCl
81	S1775	Thiotepa
82	S4007	Pentamidine isethionate
83	S2762	Alectinib (CH5424802)
84	S2348	Rotenone (Barbasco)
85	S1869	Dapoxetine HCl
86	S2536	Miconazole
87	S2541	Clomipramine HCl
88	S1328	Etodolac
89	S1497	Pralatrexate
90	S3817	Harmine hydrochloride
91	S1625	Ethinyl Estradiol
92	S3914	α-Hederin
93	S1148	Docetaxel
94	S2552	Azelastine HCl
95	S1714	Gemcitabine
96	S1267	Vemurafenib (PLX4032, RG7204)
97	S2030	Flunarizine 2HCl
98	S4504	6-Mercaptopurine (6-MP) Monohydrate
99	S1150	Paclitaxel
100	S4354	Thiostrepton
101	S1210	Methotrexate
102	S1035	Pazopanib HCl (GW786034 HCl)
103	S1949	Menadione
104	S1289	Carmofur

105	S2303	gossypol-Acetic acid
106	S1225	Etoposide
107	S1330	Felbamate
108	S3883	Protopine
109	S3692	N-Ethylmaleimide (NEM)
110	S3738	Travoprost
111	S4237	Primaquine Diphosphate
112	S1378	Ruxolitinib (INCB018424)
113	S2226	Idelalisib (CAL-101, GS-1101)
114	S1199	Cladribine
115	S2321	Magnolol
116	S1098	Rucaparib (AG-014699,PF-01367338) phosphate
117	S1994	Lacidipine
118	S1026	Imatinib Mesylate (STI571)
119	S3713	Moxidectin
120	S2246	Abiraterone Acetate
121	S1491	Fludarabine
122	S1813	Amlodipine Besylate
123	S1218	Clofarabine
124	S1229	Fludarabine Phosphate
125	S2001	Elvitegravir (GS-9137, JTK-303)
126	S1644	Nitrofural
127	S3161	Sertaconazole nitrate
128	S3868	Harmine
129	S3643	Amitraz
130	S1319	Costunolide
131	S1973	Cyclocytidine HCl
132	S1987	Mometasone furoate
133	S1613	Silodosin
134	S4285	Ospemifene
135	S2797	Lonafarnib
136	S3183	Amitriptyline HCl
137	S4283	Cyclobenzaprine HCl
138	S3966	Nifuratel
139	S1025	Gefitinib (ZD1839)
140	S1514	Cyclosporine
141	S1044	Temsirolimus (CCI-779, NSC 683864)
142	S3621	Pazufloxacin mesylate
143	S1833	Butoconazole nitrate
144	S2037	Candesartan Cilexetil
145	S2286	Cyclosporin A
146	S1501	Mycophenolate Mofetil
147	S1836	Albendazole Oxide
148	S1610	Metolazone
149	S4286	Anidulafungin (LY303366)
150	S1200	Decitabine
151	S2188	Phenprocoumon
152	S2265	Artesunate
153	S4646	Ciclesonide
154	S1880	Roxatidine Acetate HCl
155	S1956	Miconazole Nitrate
156	S1959	Tolfenamic Acid
157	S4011	Ampiroxicam
158	S1293	Cilnidipine

159	S1733	Methylprednisolone
160	S4228	Fluorometholone Acetate
161	S1712	Deferasirox
162	S2006	Pyrimethamine
163	S1299	Floxuridine
164	S3747	Levothyroxine sodium
165	S2169	Rosuvastatin Calcium
166	S1443	Zileuton
167	S1652	Monobenzone
168	S1008	Selumetinib (AZD6244)
169	S4012	Desloratadine
170	S1060	Olaparib (AZD2281, Ku-0059436)
171	S1782	Azacitidine
172	S1156	Capecitabine
173	S3080	Etravirine (TMC125)
174	S3618	Acetylspiramycin (ASPM)
175	S1905	Amlodipine
176	S4120	Sulconazole Nitrate
177	S1426	Repaglinide
178	S4221	Benzbromarone
179	S4690	Escin
180	S4674	Hydroxyprogesterone caproate
181	S1137	Malotilate
182	S1483	Iloperidone
183	S1431	Sildenafil Citrate
184	S3895	Sophoridine
185	S1227	Raloxifene HCl
186	S3876	Indigo
187	S2126	Naftopidil
188	S4181	Nicardipine HCl
189	S1403	Tigecycline
190	S3189	Ropinirole HCl
191	S3722	Isavuconazole
192	S1885	Felodipine
193	S2125	Mestranol
194	S1468	Alfacalcidol
195	S3038	Fosaprepitant dimeglumine salt
196	S2741	Niraparib (MK-4827)
197	S2229	Eltrombopag Olamine
198	S1276	Adapalene
199	S1778	Trifluridine
200	S3163	Benztropine mesylate
201	S1214	Bleomycin sulfate
202	S3872	Guaiacol
203	S1386	Nafamostat Mesylate
204	S2062	Tiopronin
205	S1601	Reserpine
206	S2310	Honokiol
207	S1022	Ridaforolimus (Deforolimus, MK-8669)
208	S1202	Dutasteride
209	S2603	Tioxolone
210	S1914	Pregnenolone
211	S1430	Rolipram
212	S4080	Triamterene

213	S1883	Idoxuridine
214	S3149	Estradiol valerate
215	S2487	Mycophenolic acid
216	S3706	Sarpogrelate hydrochloride
217	S1457	Atazanavir Sulfate
218	S3144	Darifenacin HBr
219	S2470	Fluocinolone Acetonide
220	S2515	Vardenafil HCl Trihydrate
221	S1718	Adefovir Dipivoxil
222	S2673	Trametinib (GSK1120212)
223	S2606	Mifepristone
224	S1839	Chloroxine
225	S1387	Naftopidil DiHCl
226	S1191	Fulvestrant
227	S4552	Bithionol
228	S1465	Moxifloxacin HCl
229	S1854	Bifonazole
230	S1394	Pizotifen Malate
231	S1467	Doxercalciferol
232	S1992	Fluticasone propionate
233	S1185	Ritonavir
234	S3951	Tannic acid
235	S3716	Flibanserin
236	S4518	Chloroxylenol
237	S3000	Carbazochrome sodium sulfonate (AC-17)
238	S4056	Retapamulin
239	S1458	VX-745
240	S2567	Medroxyprogesterone acetate
241	S1547	Febuxostat
242	S1604	Olmesartan Medoxomil
243	S4259	Vilazodone HCl
244	S1082	Vismodegib (GDC-0449)
245	S1283	Asenapine maleate
246	S4059	Sodium Nitroprusside Dihydrate
247	S1055	Enzastaurin (LY317615)
248	S1435	Tamsulosin hydrochloride
249	S1284	Benazepril HCl
250	S4381	Proadifen HCl
251	S1505	Aztreonam
252	S2354	Sclareol
253	S3959	(+)-Borneol
254	S1676	Amorolfine HCl
255	S1007	Roxadustat (FG-4592)
256	S1735	Mesna
257	S2041	Dyclonine HCl
258	S1322	Dexamethasone (DHAP)
259	S1920	Haloperidol
260	S1368	Acitretin
261	S1466	Calcitriol
262	S1358	Loratadine
263	S2261	Andrographolide
264	S4601	Clioquinol
265	S1593	Apixaban
266	S1773	Oxytetracycline (Terramycin)

267	S1859	Diethylstilbestrol
268	S1472	Safinamide Mesylate
269	S4585	Succinylsulfathiazole
270	S1685	Sulfanilamide
271	S1192	Raltitrexed
272	S4054	Spironolactone
273	S2459	Clozapine
274	S1895	Dipyridamole
275	S2584	Clobetasol propionate
276	S3674	Levamlodipine
277	S4751	Cisapride hydrate
278	S4079	Ticagrelor
279	S1627	Nitazoxanide
280	S4072	Decamethonium Bromide
281	S1832	Atracurium Besylate
282	S3003	Prostaglandin E2 (PGE2)
283	S3081	Ulipristal Acetate (CDB 2914)
284	S1669	Loteprednol etabonate
285	S3899	Hederagenin
286	S1974	Meglumine
287	S1233	2-Methoxyestradiol (2-MeOE2)
288	S2422	Ipriflavone (Osteofix)
289	S1499	Cefaclor
290	S1209	Fluorouracil (5-Fluoracil, 5-FU)
291	S4343	Oxethazaine
292	S1743	NEXIUM (esomeprazole magnesium)
293	S2473	Hexestrol
294	S3728	Grazoprevir
295	S1649	Zolmitriptan
296	S2534	Isoconazole nitrate
297	S2040	Nimesulide
298	S1673	Aminophylline
299	S1005	Axitinib
300	S2295	Emodin
301	S1688	Betamethasone Dipropionate
302	S1425	Ranolazine 2HCl
303	S1794	Fenofibrate
304	S4609	Diflunisal
305	S4274	Rotigotine
306	S2535	Econazole nitrate
307	S2043	Memantine HCl
308	S1763	Quetiapine Fumarate
309	S1754	Oxybutynin
310	S2064	Balofloxacin
311	S1646	Ketorolac
312	S3012	Pazopanib
313	S2500	Propafenone HCl
314	S4078	Mefenamic Acid
315	S2118	Ibutilide Fumarate
316	S1805	Acetylcholine Chloride
317	S1738	Telmisartan
318	S2721	Nilvadipine
319	S2087	Rivastigmine Tartrate
320	S1799	Ranolazine

321	S1569	Tazarotene
322	S2066	Moxonidine
323	S1376	Gestodene
324	S2922	Icotinib
325	S1039	Rapamycin (Sirolimus)
326	S1278	Altretamine
327	S3002	Rivaroxaban
328	S2067	Ozagrel HCl
329	S1910	Tioconazole
330	S3746	Lumefantrine
331	S4416	Trimipramine Maleate
332	S3024	Lamotrigine
333	S2332	Neohesperidin
334	S2044	Cyproheptadine HCl
335	S2365	Tanshinone IIA
336	S1760	Rifapentine
337	S1620	Darunavir Ethanolate
338	S1286	Budesonide
339	S4227	Fidaxomicin
340	S2450	Equol
341	S1382	Mianserin HCl
342	S2271	Berberine chloride
343	S1835	Azithromycin
344	S2318	Lappaconitine
345	S1380	Lopinavir
346	S4602	Acetohydroxamic acid
347	S4207	Clofibric Acid
348	S2358	Silymarin
349	S1488	Naratriptan HCl
350	S1808	Nifedipine
351	S2038	Phentolamine Mesylate
352	S4038	Dibucaine HCl
353	S1404	Trilostane
354	S2790	Istradefylline
355	S2208	Formestane
356	S1941	Enalapril Maleate
357	S2060	Bromhexine HCl
358	S1816	Chlorpheniramine Maleate
359	S1238	Tamoxifen
360	S1603	Furosemide
361	S2344	Piperine
362	S2256	4-Methylumbelliferone (4-MU)
363	S1960	Pranoprofen
364	S4502	Eltrombopag
365	S1439	Tranilast
366	S1811	Amiloride HCl
367	S1741	Rifabutin
368	S4046	Estradiol Cypionate
369	S1655	Ezetimibe
370	S4797	Nicergoline
371	S2471	Gallamine Triethiodide
372	S1716	Glyburide (Glibenclamide)
373	S4727	Cinnarizine
374	S3714	Lifitegrast

375	S2085	Trimebutine
376	S1748	Nisoldipine
377	S2807	Dabrafenib (GSK2118436)
378	S1713	Piroxicam
379	S1482	Daclatasvir (BMS-790052)
380	S2293	DL-Carnitine HCl
381	S4373	Dicyclomine HCl
382	S1807	Aciclovir
383	S2787	Laquinimod
384	S1727	Levonorgestrel
385	S1651	Telbivudine
386	S2349	Rutaecarpine
387	S1385	Mosapride Citrate
388	S1606	Clotrimazole
389	S1777	Ethionamide
390	S1618	Sulfameter
391	S1633	Zafirlukast
392	S4091	Ifenprodil Tartrate
393	S1657	Enalaprilat Dihydrate
394	S3079	Atovaquone
395	S2499	Phenoxybenzamine HCl
396	S1336	Fluvoxamine maleate
397	S1829	Pranlukast
398	S2479	Lincomycin HCl
399	S2091	Betaxolol
400	S1689	Meprednisone
401	S4264	Etofibrate
402	S1304	Megestrol Acetate
403	S1690	Betamethasone Valerate
404	S2058	Tolnaftate
405	S2108	Flunixin Meglumin
406	S1312	Streptozotocin (STZ)
407	S3984	Nordihydroguaiaretic acid (NDGA)
408	S2452	Amfebutamone (Bupropion) HCl
409	S1261	Celecoxib
410	S2029	Uridine
411	S2287	Cytisine
412	S3635	Medroxyprogesterone
413	S1737	Prednisolone
414	S2101	Gabexate Mesylate
415	S1271	Acarbose
416	S1614	Riluzole
417	S3023	Bufexamac
418	S1196	Exemestane
419	S3052	Rupatadine Fumarate
420	S2090	Dexmedetomidine HCl
421	S4230	Oxaprozin
422	S1540	Saxagliptin
423	S2320	Luteolin
424	S2042	Cyproterone Acetate
425	S1235	Letrozole
426	S1681	Mesalamine
427	S3727	Vilanterol Trifenate
428	S1331	Fluconazole

429	S1324	Doxazosin Mesylate
430	S1705	Progesterone
431	S1029	Lenalidomide (CC-5013)
432	S1801	Ranitidine Hydrochloride
433	S2059	Terazosin HCl Dihydrate
434	S4588	Docusate Sodium
435	S1626	Naproxen Sodium
436	S4089	Halobetasol Propionate
437	S1820	Clofibrate
438	S1784	Vidarabine
439	S2109	Imidapril HCl
440	S2380	Diosmetin
441	S1381	Meropenem
442	S2015	Suplatast Tosylate
443	S2262	Apigenin
444	S1643	Ursodiol
445	S2304	Gramine
446	S2461	Domperidone
447	S1247	Leflunomide
448	S1538	Telaprevir (VX-950)
449	S2053	Cytidine
450	S4026	Hydroxyzine 2HCl
451	S2065	Lafutidine
452	S1653	Tretinoin
453	S1548	Dapagliflozin
454	S2123	Dextrose
455	S1444	Ziprasidone HCl
456	S1921	Phenindione
457	S3001	Clevudine
458	S4528	Furazolidone
459	S1639	Amprenavir
460	S1396	Resveratrol
461	S4547	8-Hydroxyquinoline
462	S1291	Cetirizine DiHCl
463	S2154	Dabigatran Etexilate
464	S4195	Broxyquinoline
465	S3036	Pravastatin sodium
466	S3733	Boceprevir
467	S2289	Daidzin
468	S2082	Adiphenine HCl
469	S3120	Doxepin HCl
470	S2092	Detomidine HCl
471	S1401	Tenofovir
472	S1294	Cilostazol
473	S2364	Tanshinone I
474	S1407	Bimatoprost
475	S1764	Rifampin
476	S4685	Efavirenz
477	S2131	Roflumilast
478	S2907	Pirfenidone
479	S3781	Ginkgolide C
480	S2593	Tolvaptan
481	S2083	Procaterol HCl
482	S3160	Ethynodiol diacetate

483	S3704	4-Methylbenzylidene camphor
484	S1884	Sparfloxacin
485	S4203	Furaltadone HCl
486	S4281	Tasimelteon
487	S1342	Genistein
488	S4638	Desogestrel
489	S3033	Vildagliptin (LAF-237)
490	S2437	Rotundine
491	S4019	Avanafil
492	S2099	Temocapril HCl
493	S1762	Pyrazinamide
494	S4561	Danthron
495	S2569	Phenylephrine HCl
496	S4110	Estradiol Benzoate
497	S1379	Isotretinoin
498	S3121	Ornidazole
499	S2233	Esomeprazole sodium
500	S1888	Deflazacort
501	S4683	Sildenafil Mesylate
502	S2250	(-)-Epigallocatechin Gallate
503	S1374	Doripenem Hydrate
504	S2903	Lumiracoxib
505	S3675	Umbelliferone
506	S1221	Dacarbazine
507	S2390	Polydatin
508	S1014	Bosutinib (SKI-606)
509	S1377	Drospirenone
510	S4292	Diphenidol HCl
511	S4165	Benzydamine HCl
512	S2102	Rasagiline Mesylate
513	S1197	Finasteride
514	S4584	Butylparaben
515	S4155	Chlorzoxazone
516	S3124	Dexamethasone Acetate
517	S3083	Indacaterol Maleate
518	S2900	Cobicistat (GS-9350)
519	S1332	Flumazenil
520	S1855	Pefloxacin Mesylate
521	S1865	Diltiazem HCl
522	S3167	Altrenogest
523	S1768	Cefditoren Pivoxil
524	S1354	Lansoprazole
525	S1806	Acipimox
526	S2309	Hesperidin
527	S4718	Acetylcholine iodide
528	S2359	Sinomenine
529	S1978	Methscopolamine
530	S1405	Vecuronium Bromide
531	S2086	Ivabradine HCl
532	S2357	Silibinin
533	S3751	Quinidine sulfate
534	S1645	Ketoprofen
535	S1445	Zonisamide
536	S2454	Bupivacaine HCl

537	S3732	Avibactam sodium
538	S1344	Glimepiride
539	S1243	Agomelatine
540	S1607	Rizatriptan Benzoate
541	S4249	Flopropione
542	S4362	Glafenine HCl
543	S1334	Flupirtine maleate
544	S4051	Nabumetone
545	S2346	Puerarin
546	S1878	Ganciclovir
547	S1326	Edaravone
548	S4389	Bephenium Hydroxynaphthoate
549	S1010	Nintedanib (BIBF 1120)
550	S2054	Orphenadrine Citrate
551	S1756	Enoxacin
552	S1898	Tropisetron HCl
553	S1206	Bisoprolol fumarate
554	S1188	Anastrozole
555	S4607	2,2′-Dihydroxy-4-methoxybenzophenone
556	S2391	Quercetin
557	S1280	Amisulpride
558	S1845	Cimetidine
559	S1896	Hydroxyurea
560	S1662	Isradipine
561	S4612	Dapson
562	S1565	VX-809 (Lumacaftor)
563	S2124	Xylose
564	S1437	Tizanidine HCl
565	S3200	Triflusal
566	S1770	Sulfadiazine
567	S1636	Amphotericin B
568	S1903	Diclofenac Sodium
569	S2443	Tolbutamide
570	S2024	Ketotifen Fumarate
571	S1866	Diphenhydramine HCl
572	S4253	Epinastine HCl
573	S1734	Meloxicam
574	S3175	Atomoxetine HCl
575	S4049	Valdecoxib
576	S2386	Indirubin
577	S1611	Cefoperazone
578	S3031	Linagliptin
579	S2542	Phenformin HCl
580	S1591	Bestatin
581	S1964	Rimantadine
582	S2373	Yohimbine HCl
583	S2051	Captopril
584	S1933	Triamcinolone
585	S3654	Tauroursodeoxycholic Acid (TUDCA)
586	S2007	Sulindac
587	S4021	Tolcapone
588	S1245	Latrepirdine 2HCl
589	S1683	Ipratropium Bromide
590	S4189	Cyclandelate

591	S1761	Suprofen
592	S1193	Thalidomide
593	S1890	Nizatidine
594	S2336	Orotic acid (6-Carboxyuracil)
595	S4031	Aclidinium Bromide
596	S1793	Ramipril
597	S4268	Flufenamic acid
598	S3893	Bornyl acetate
599	S2052	Oxytetracycline Dihydrate
600	S2760	Canagliflozin
601	S1849	Daidzein
602	S2410	Paeoniflorin
603	S1950	Metformin HCl
604	S4113	Desvenlafaxine
605	S1742	Nevirapine
606	S2411	Geniposide
607	S1916	Sulfisoxazole
608	S2383	Gastrodin
609	S4260	Tamibarotene
610	S1971	Nicorandil
611	S2328	Nalidixic acid
612	S2130	Atropine sulfate monohydrate
613	S1222	Dexrazoxane HCl (ICRF-187, ADR-529)
614	S1894	Valsartan
615	S1907	Metronidazole
616	S1717	Fomepizole
617	S4673	Etonogestrel
618	S2883	4-Aminohippuric Acid
619	S2199	Aliskiren Hemifumarate
620	S4309	Bromocriptine Mesylate
621	S2607	Buflomedil HCl
622	S2025	Urapidil HCl
623	S4571	Hexylresorcinol
624	S3117	Oxybutynin chloride
625	S3054	Alverine Citrate
626	S4034	Diphemanil Methylsulfate
627	S1709	Estradiol
628	S2339	Paeonol
629	S2362	Synephrine
630	S3724	Velpatasvir
631	S2503	Racecadotril
632	S4086	Loxapine Succinate
633	S1701	Desonide
634	S1696	Hydrocortisone
635	S2393	Sorbitol
636	S1908	Flutamide
637	S4173	1-Hexadecanol
638	S1635	Erythromycin
639	S2325	Morin Hydrate
640	S4559	Cloxiquine
641	S3854	Tetrahydropalmatine hydrochloride
642	S2127	S- (+)-Rolipram
643	S1250	Enzalutamide (MDV3100)
644	S1952	Methoxsalen

645	S3045	Cinepazide maleate
646	S1164	Lenvatinib (E7080)
647	S2608	Fluocinonide
648	S3647	Mafenide Acetate
649	S2078	Famotidine
650	S2508	Scopolamine HBr
651	S1212	Bendamustine HCl
652	S3739	Calcipotriene
653	S1441	Venlafaxine HCl
654	S1747	Nimodipine
655	S4114	Triclabendazole
656	S4734	Retigabine 2HCl
657	S2119	Probucol
658	S1899	Nicotinamide (Vitamin B3)
659	S1702	Didanosine
660	S2258	Esculin
661	S4699	Etretinate
662	S2614	Arecoline HBr
663	S3741	Benznidazole
664	S4225	Mexiletine HCl
665	S2381	D-Mannitol
666	S4377	Imipramine HCl
667	S4288	Chloroambucil
668	S2097	Ambrisentan
669	S2069	Argatroban
670	S3078	Beclomethasone dipropionate
671	S3919	Hederacoside C
672	S1631	Allopurinol Sodium
673	S1658	Dofetilide
674	S2296	Enoxolone
675	S2113	Cisatracurium Besylate
676	S2874	Camostat Mesilate
677	S2351	Salicin
678	S4591	Nitroxoline
679	S2384	Hematoxylin
680	S2596	Clindamycin palmitate HCl
681	S2282	Cinchonidine
682	S3186	Azatadine dimaleate
683	S1576	Sulfasalazine
684	S1715	Glipizide
685	S2438	Synephrine HCl
686	S4250	Sulfamethoxypyridazine
687	S2177	Dichlorphenamide (Diclofenamide)
688	S3998	(+)-α-Lipoic acid
689	S4102	Eprosartan Mesylate
690	S3074	Chlorthalidone
691	S1619	Prilocaine
692	S1578	Candesartan
693	S2506	Roxithromycin
694	S4839	Mosapride
695	S1340	Gatifloxacin
696	S4200	Tolperisone HCl
697	S4202	Verapamil HCl
698	S2016	Mirtazapine

699	S1962	Sulphadimethoxine
700	S1679	Flurbiprofen
701	S3824	Quercitrin
702	S2611	Ethisterone
703	S2579	Zidovudine
704	S4071	Griseofulvin
705	S4169	Teriflunomide
706	S1732	Mitotane
707	S1677	Chloramphenicol
708	S1204	Melatonin
709	S2302	Glycyrrhizin (Glycyrrhizic Acid)
710	S2467	Famciclovir
711	S2011	Pramipexole 2HCl Monohydrate
712	S2055	Gimeracil
713	S2266	Asiatic Acid
714	S4098	Halcinonide
715	S1897	Potassium Iodide
716	S4106	Closantel
717	S2388	L-carnitine
718	S3043	Rofecoxib
719	S3847	Panaxatriol
720	S1904	Avobenzone
721	S3759	Norcantharidin
722	S1287	Bumetanide
723	S4147	Azithromycin Dihydrate
724	S3892	Isopsoralen
725	S1915	Sulfamethoxazole
726	S4507	17-Hydroxyprogesterone
727	S4187	Salicylanilide
728	S1517	Natamycin
729	S4583	Butamben
730	S1703	Divalproex Sodium
731	S3017	Aspirin
732	S2036	Aspartame
733	S2533	Ritodrine HCl
734	S1675	Lubiprostone
735	S2446	Levosimendan
736	S3677	Cinnamic acid
737	S1891	Carbidopa
738	S3731	Tipiracil hydrochloride
739	S2451	Amantadine HCl
740	S1881	Protionamide
741	S4604	Levofloxacin hydrate
742	S4590	Dithranol
743	S1706	Lamivudine
744	S1691	Praziquantel
745	S4672	Dimenhydrinate
746	S2314	Kaempferol
747	S4159	Bezafibrate
748	S3641	Osalmid
749	S3027	Fenoprofen calcium hydrate
750	S4077	Mequinol
751	S3105	Nadifloxacin
752	S2389	Naringin Dihydrochalcone

753	S2331	Neohesperidin Dihydrochalcone (Nhdc)
754	S1858	Dienestrol
755	S2103	Naltrexone HCl
756	S1408	Linezolid
757	S2299	Formononetin
758	S2492	Novobiocin Sodium
759	S1442	Voriconazole
760	S1573	Fasudil (HA-1077) HCl
761	S4065	Guanabenz Acetate
762	S2080	Clevidipine Butyrate
763	S3776	Sophoricoside
764	S1665	Estrone
765	S2322	(+)-Matrine
766	S3133	Sulfamethazine
767	S2543	Ceftiofur HCl
768	S1823	Fenoprofen Calcium
769	S4178	Climbazole
770	S2316	Kinetin
771	S3648	Amlexanox
772	S2046	Pioglitazone HCl
773	S2072	Seratrodast(AA-2414, ABT-001)
774	S2525	Phenytoin
775	S4838	Acotiamide hydrochloride
776	S1438	Topiramate
777	S2032	Rebamipide
778	S1940	Levofloxacin
779	S2329	Naringin
780	S4706	Eugenol
781	S4711	Esculetin
782	S4210	Benzocaine
783	S4096	Droperidol
784	S1629	Orlistat
785	S1282	Artemisinin
786	S2105	Pantoprazole
787	S2396	Salidroside
788	S1827	Betaxolol HCl
789	S2338	Oxymatrine
790	S2439	Guanosine
791	S2347	Quercetin Dihydrate
792	S1730	Indapamide
793	S3137	Sodium salicylate
794	S1605	Cefdinir
795	S3181	Flumequine
796	S4160	Penicillin G Sodium
797	S4057	Methyclothiazide
798	S3645	Kitasamycin
799	S1628	Triamcinolone Acetonide
800	S3185	Adrenalone HCl
801	S4176	Trometamol
802	S2519	Naphazoline HCl
803	S1389	Omeprazole
804	S4317	Clorgyline HCl
805	S3138	Methylthiouracil
806	S3701	Benactyzine hydrochloride

807	S1840	Lomustine
808	S4258	Luliconazole
809	S3673	Sulfaphenazole
810	S1790	Rifaximin
811	S3132	Sulfamerazine
812	S4208	Chromocarb
813	S2369	Troxerutin
814	S2401	Sodium Danshensu
815	S2337	Osthole
816	S2378	Butylscopolamine Bromide
817	S4039	Methazolamide
818	S3179	Carbenicillin disodium
819	S4105	Closantel Sodium
820	S1957	Sulfamethizole
821	S3212	Moclobemide (Ro 111163)
822	S2020	Formoterol Hemifumarate
823	S2537	Secnidazole
824	S4256	Buspirone HCl
825	S1876	Valaciclovir HCl
826	S4535	Methylene Blue
827	S3842	Isoquercitrin
828	S3063	Diclofenac Diethylamine
829	S1937	Isoniazid
830	S1856	Metoprolol Tartrate
831	S4709	Latanoprost
832	S3924	Ginsenoside Rb1
833	S4263	Efaproxiral Sodium
834	S3730	Metaxalone
835	S3711	Carbasalate Calcium
836	S4334	Mesoridazine Besylate
837	S3140	Milnacipran HCl
838	S4402	Oxeladin Citrate
839	S2317	L-(+)-Rhamnose Monohydrate
840	S2868	Alogliptin（SYK-322）benzoate
841	S1654	Phenylbutazone
842	S1789	Tetrabenazine (Xenazine)
843	S1988	Propylthiouracil
844	S1512	Tadalafil
845	S4216	Valnemulin HCl
846	S2875	Prucalopride
847	S3887	L-Rhamnose monohydrate
848	S1415	Clopidogrel
849	S3077	Tazobactam
850	S4744	Anethole trithione
851	S4577	Terpin hydrate
852	S2594	Pramiracetam
853	S1255	Nepafenac
854	S3846	Eupatilin
855	S3070	Piracetam
856	S2308	Hesperetin
857	S2021	Chlormezanone
858	S3130	Biotin (Vitamin B7)
859	S3622	Diammonium Glycyrrhizinate
860	S3995	Guaiazulene

861	S1409	Alfuzosin HCl
862	S3735	Umeclidinium bromide
863	S2547	Tiotropium Bromide hydrate
864	S1693	Carbamazepine
865	S1622	Prednisone
866	S2453	Benserazide HCl
867	S1739	Thiabendazole
868	S4143	Pentoxyverine Citrate
869	S2263	Arbutin
870	S3935	Nonivamide
871	S3945	L-Cycloserine
872	S1500	Betamethasone
873	S3797	Helicide
874	S2830	Clindamycin
875	S1623	Acetylcysteine
876	S2270	Bergenin
877	S4188	Sasapyrine
878	S3672	Cefonicid sodium
879	S3690	Pargyline hydrochloride
880	S4284	Chloroprocaine HCl
881	S3021	Rimonabant
882	S1725	Terbinafine
883	S4100	Esmolol HCl
884	S4217	Liothyronine Sodium
885	S1359	Losartan Potassium (DuP 753)
886	S4578	Tyloxapol
887	S1608	Pyridostigmine Bromide
888	S1251	Dienogest
889	S3151	Gliquidone
890	S4565	Diiodohydroxyquinoline
891	S3835	Loganin
892	S3965	Vanillyl Butyl Ether
893	S2375	Aloin
894	S3766	Tanshinone IIA sulfonate (sodium)
895	S2057	Cyclophosphamide Monohydrate
896	S3659	Fludrocortisone acetate
897	S1704	Emtricitabine
898	S2003	Maraviroc
899	S1828	Proparacaine HCl
900	S2840	Apalutamide?(ARN-509)
901	S2509	Sotalol HCl
902	S4064	Escitalopram Oxalate
903	S1647	Adenosine
904	S2458	Clonidine HCl
905	S2045	Doxifluridine
906	S1300	Tegafur (FT-207, NSC 148958)
907	S4040	Norethindrone
908	S2576	Xylometazoline HCl
909	S1252	Entecavir Hydrate
910	S4037	Doxapram HCl
911	S3202	Catharanthine
912	S2524	Phenytoin Sodium
913	S1729	Gemfibrozil
914	S4239	Bergapten

915	S1213	Nelarabine
916	S1791	Bacitracin Zinc
917	S2493	Olanzapine
918	S1256	Rufinamide
919	S3809	Imperatorin
920	S4082	Spiramycin
921	S3640	Methoxyphenamine Hydrochloride
922	S1928	Alibendol
923	S2366	Taxifolin (Dihydroquercetin)
924	S2529	Dopamine HCl
925	S2581	Quinapril HCl
926	S4622	3,4-Diaminopyridine
927	S4845	Rabeprazole
928	S4737	Psoralen
929	S2510	Spectinomycin 2HCl
930	S2564	Cloxacillin Sodium
931	S3926	Forsythin
932	S3018	Niflumic acid
933	S1990	Capsaicin(Vanilloid)
934	S1744	Nicotinic Acid
935	S4081	Sulfacetamide Sodium
936	S4139	Cyclizine 2HCl
937	S3967	Flavone
938	S1258	Prasugrel
939	S1373	Daptomycin
940	S2376	Ammonium Glycyrrhizinate
941	S3173	Antipyrine
942	S3668	Thymopentin
943	S2096	Almotriptan Malate
944	S2387	Lappaconite HBr
945	S4111	Dicloxacillin Sodium
946	S3811	Ginsenoside Re
947	S2071	Prulifloxacin (NM441, AF 3013)
948	S1553	ABT-492(Delafloxacin, RX-3341, WQ-3034)
949	S1183	Danoprevir (ITMN-191)
950	S3154	Butenafine HCl
951	S4104	Diminazene Aceturate
952	S3779	cis-Anethole
953	S3705	Chlorobutanol
954	S3170	Ampicillin sodium
955	S4061	Levobupivacaine HCl
956	S4118	Histamine 2HCl
957	S3889	Arteether
958	S4837	Ibudilast
959	S4214	Sucralose
960	S1723	Indomethacin
961	S2599	L-Thyroxine
962	S3703	Phenethyl alcohol
963	S3625	Tyramine
964	S4543	Trimetazidine dihydrochloride
965	S3655	Cefepime Dihydrochloride Monohydrate
966	S3669	Carmustine
967	S2504	Ribavirin
968	S1666	Flucytosine

969	S4018	Sennoside B
970	S1692	Busulfan
971	S3062	Diclofenac Potassium
972	S2350	Rutin
973	S3763	Cinnamaldehyde
974	S2280	Chlorogenic Acid
975	S1397	Rocuronium Bromide
976	S3986	L(+)-Arabinose
977	S3774	Dehydroandrographolide Succinate Potasium Salt
978	S2665	Ciprofibrate
979	S2610	Lonidamine
980	S1736	Methocarbamol
981	S3983	Caryophyllene oxide
982	S3987	L-Tryptophan
983	S2583	Thiamphenicol
984	S2577	Phenacetin
985	S3116	Sulfathiazole
986	S4083	Vitamin A Acetate
987	S4161	Benzoic Acid
988	S2466	Estriol
989	S4665	Rebeprazole sodium
990	S1356	Levetiracetam
991	S1667	Trichlormethiazide
992	S2512	Tenoxicam
993	S3907	Bulleyaconi cine A
994	S2570	Prednisolone Acetate
995	S2264	Artemether
996	S4060	Erythromycin Ethylsuccinate
997	S3843	Madecassoside
998	S4243	Deoxycorticosterone acetate
999	S4624	5,5-Dimethyloxazolidine-2,4-dione
1000	S4231	Pilocarpine HCl
1001	S1934	Nystatin (Fungicidin)
1002	S2269	Baicalin
1003	S4351	Ractopamine HCl
1004	S1391	Oxcarbazepine
1005	S3104	Moguisteine
1006	S2501	Pyrantel Pamoate
1007	S3802	Trigonelline Hydrochloride
1008	S1641	Chlorothiazide
1009	S4544	Urethane
1010	S4248	Bromfenac Sodium
1011	S3129	Trimethoprim
1012	S3694	Glucosamine hydrochloride
1013	S3666	Ilaprazole
1014	S4190	Cinchophen
1015	S3785	Notoginsenoside R1
1016	S1913	Tropicamide
1017	S2268	Baicalein
1018	S3180	Eletriptan HBr
1019	S4048	Carbimazole
1020	S4167	Cyromazine
1021	S2884	Acesulfame Potassium
1022	S2574	Tetracycline HCl

1023	S4812	Ceftizoxime
1024	S3726	Selexipag
1025	S4045	Pheniramine Maleate
1026	S1400	Tenofovir Disoproxil Fumarate
1027	S4166	Chlorpropamide
1028	S1843	Chenodeoxycholic Acid
1029	S4566	DL-Panthenol
1030	S2902	S-Ruxolitinib (INCB018424)
1031	S3681	Vitamin E Acetate
1032	S4831	Piperonyl butoxide
1033	S2555	Clarithromycin
1034	S3769	Palmatine
1035	S4536	Nitrofurantoin
1036	S4184	Penciclovir
1037	S2521	Epinephrine bitartrate
1038	S2079	Moexipril HCl
1039	S1969	Nefiracetam
1040	S4204	Isosorbide
1041	S2300	Ferulic Acid
1042	S4635	Cyproheptadine hydrochloride
1043	S3708	Sulfachloropyridazine
1044	S3791	Succinic acid
1045	S2374	5-hydroxytryptophan (5-HTP)
1046	S3155	Mepivacaine HCl
1047	S4349	Procyclidine HCl
1048	S2005	Raltegravir (MK-0518)
1049	S4191	Betamipron
1050	S4095	Difluprednate
1051	S2516	Xylazine HCl
1052	S2809	MPEP
1053	S4640	Lesinurad
1054	S4331	Meclocycline Sulfosalicylate
1055	S4235	Phenazopyridine HCl
1056	S3061	Epinephrine HCl
1057	S2667	Dolutegravir (GSK1349572)
1058	S4332	Medrysone
1059	S2442	Inosine
1060	S4772	alpha-Asarone
1061	S2104	Levosulpiride
1062	S4698	Vitamin K1
1063	S4393	Cephapirin Sodium
1064	S3858	Lawsone
1065	S2397	Palmatine chloride
1066	S3756	Methyl salicylate
1067	S3199	Reboxetine mesylate
1068	S1609	Methimazole
1069	S4661	Tiagabine hydrochloride
1070	S3208	Fexofenadine HCl
1071	S2565	Amoxicillin Sodium
1072	S3783	Echinacoside
1073	S4017	Allylthiourea
1074	S3100	2-Thiouracil
1075	S4720	Cefotaxime
1076	S4044	Toltrazuril

1077	S4179	Mezlocillin Sodium
1078	S2496	Ozagrel
1079	S3742	Cholic acid
1080	S4009	Mirabegron
1081	S3015	Amoxicillin
1082	S4136	Carprofen
1083	S3106	Pidotimod
1084	S4197	Bemegride
1085	S3772	5-Hydroxymethylfurfural
1086	S3172	Anagrelide HCl
1087	S1508	Alprostadil
1088	S4025	Homatropine Bromide
1089	S3745	Balsalazide disodium
1090	S4367	Suxibuzone
1091	S4820	Diastase
1092	S3147	Entacapone
1093	S3968	Histamine
1094	S1719	Zalcitabine
1095	S2789	Tofacitinib (CP-690550,Tasocitinib)
1096	S3656	Piribedil
1097	S4336	Metaraminol Bitartrate
1098	S4240	Doxylamine Succinate
1099	S1237	Temozolomide
1100	S4112	Desvenlafaxine Succinate
1101	S2609	Inulin
1102	S4125	Sodium Phenylbutyrate
1103	S4733	Retigabine
1104	S3997	Oxaceprol
1105	S4587	Pentylenetetrazol
1106	S3113	Pyridoxine HCl
1107	S4270	Oxiracetam
1108	S3758	Sinomenine hydrochloride
1109	S3970	Vindoline
1110	S4177	Uracil
1111	S4224	Erythritol
1112	S3687	Urea
1113	S1162	PA-824
1114	S4326	Ethoxzolamide
1115	S4647	Cefmenoxime hydrochloride
1116	S3921	Lathyrol
1117	S2792	Torcetrapib
1118	S4522	Dehydroacetic acid
1119	S4841	Laurocapram
1120	S2586	Dimethyl Fumarate
1121	S2260	Amygdalin
1122	S3114	Vitamin C
1123	S4076	Propranolol HCl
1124	S1168	Valproic acid sodium salt (Sodium valproate)
1125	S4748	Ondansetron Hydrochloride Dihydrate
1126	S4510	4-Aminobenzoic acid
1127	S1357	Lidocaine
1128	S4415	Misoprostol
1129	S4062	Ronidazole
1130	S4163	Doxycycline Hyclate

1131	S2491	Nitrendipine
1132	S3930	Liquiritin
1133	S4141	Dinitolmide
1134	S3707	Ethopabate
1135	S4229	Oxybuprocaine HCl
1136	S3849	D-Galactose
1137	S4542	Trihexyphenidyl hydrochloride
1138	S3616	Asiaticoside
1139	S3805	Stevioside
1140	S4185	Tiratricol
1141	S3956	Tetramethylpyrazine
1142	S4058	Ropivacaine HCl
1143	S4593	Chlormadinone acetate
1144	S4391	Camylofin Chlorhydrate
1145	S4206	Cysteamine HCl
1146	S3051	Bosentan Hydrate
1147	S2794	Sofosbuvir (PSI-7977, GS-7977)
1148	S3810	Scutellarin
1149	S1825	Erdosteine
1150	S4010	Acebutolol HCl
1151	S4050	Valganciclovir HCl
1152	S2602	Acemetacin
1153	S3773	Tyrosol
1154	S3996	Thioctic acid
1155	S4164	Doxofylline
1156	S4750	Sulfacetamide sodium salt hydrate
1157	S1398	Stavudine (d4T)
1158	S4330	Isoetharine Mesylate
1159	S3775	Ligustrazine hydrochloride
1160	S4701	2-Deoxy-D-glucose
1161	S2497	Pancuronium dibromide
1162	S4002	Sitagliptin phosphate monohydrate
1163	S3644	Sulfamonomethoxine
1164	S3982	Batyl alcohol
1165	S4138	Dropropizine
1166	S4834	Propantheline bromide
1167	S1740	Guaifenesin
1168	S3193	Ticarcillin sodium
1169	S4099	Dexlansoprazole
1170	S1638	Ibuprofen
1171	S4149	Amfenac Sodium Monohydrate
1172	S3623	Ceftibuten dihydrate
1173	S1965	Primidone
1174	S4754	Betulin
1175	S4020	Sodium Picosulfate
1176	S4043	Tetrahydrozoline HCl
1177	S4042	Nafcillin Sodium
1178	S4246	Tranylcypromine (2-PCPA) HCl
1179	S3162	Tylosin tartrate
1180	S4707	Oleic Acid
1181	S4692	Succimer
1182	S3684	Methacholine chloride
1183	S3693	2,6-Dihydroxypurine
1184	S2455	Bethanechol chloride

1185	S3927	Swertiamarin
1186	S4576	Sulfabenzamide
1187	S3663	Afloqualone
1188	S4521	DEET
1189	S4651	Etoricoxib
1190	S4023	Procaine HCl
1191	S4581	Triacetin
1192	S3957	Gamma-Oryzanol
1193	S4375	Mepenzolate Bromide
1194	S3923	Ginsenoside Rg1
1195	S4520	2-Aminoheptane
1196	S4803	Thymidine
1197	S4664	Molsidomine
1198	S4666	Sivelestat sodium tetrahydrate
1199	S1511	Lactulose
1200	S2553	5-Aminolevulinic acid HCl
1201	S2159	Tebipenem Pivoxil
1202	S4404	Pasiniazid
1203	S4085	Levobetaxolol HCl
1204	S2585	Brompheniramine hydrogen maleate
1205	S2460	Pramipexole
1206	S1958	Sulbactam
1207	S4101	Voglibose
1208	S4833	Cefoxitin sodium
1209	S1567	Pomalidomide
1210	S4265	Nicaraven
1211	S2554	Daphnetin
1212	S3195	Azlocillin sodium salt
1213	S4662	Atazanavir
1214	S3697	Mafenide hydrochloride
1215	S4022	Probenecid
1216	S4836	Nilutamide
1217	S3807	Dehydroandrographolide
1218	S4676	Gluconolactone
1219	S4736	Trapidil
1220	S3755	Betaine
1221	S4846	Meropenem Trihydrate
1222	S3866	Galanthamine
1223	S1750	Octocrylene
1224	S4122	Tilmicosin
1225	S3723	Ramosetron Hydrochloride
1226	S4068	Tinidazole
1227	S4835	Aceclofenac
1228	S1630	Allopurinol
1229	S4506	Acetazolamide
1230	S3788	Carvacrol
1231	S4266	Brimonidine Tartrate
1232	S2277	Caffeic Acid
1233	S4648	Dantrolene sodium hemiheptahydrate
1234	S4689	Deoxycholic acid
1235	S4152	Ethamsylate
1236	S2549	Trospium chloride
1237	S4714	(-)-Menthol
1238	S4312	Carbadox

1239	S4658	Hydroquinidine
1240	S3925	(-)-Epicatechin gallate
1241	S3969	Veratric acid
1242	S4636	Teneligliptin hydrobromide
1243	S4357	Tacrine HCl
1244	S4562	Dehydrocholic acid
1245	S2502	Quinine HCl Dihydrate
1246	S2505	Rosiglitazone maleate
1247	S4527	Fenofibric acid
1248	S3657	Promestriene
1249	S4620	Cefuroxime sodium
1250	S4558	Citiolone
1251	S3153	levalbuterol tartrate
1252	S4135	Clorprenaline HCl
1253	S4830	Maltose
1254	S4556	Carzenide
1255	S3176	Betahistine 2HCl
1256	S4695	D panthenol
1257	S4024	Homatropine Methylbromide
1258	S4293	Promethazine HCl
1259	S4088	Flumethasone
1260	S4219	Azaperone
1261	S4339	Meticrane
1262	S3071	Vanillin
1263	S4517	Cefotaxime sodium
1264	S4092	Pramoxine HCl
1265	S2601	Gliclazide
1266	S4123	Timolol Maleate
1267	S4175	Sulfaguanidine
1268	S4725	Benzenesulfonamide
1269	S4366	Pinacidil
1270	S1902	Vitamin B12
1271	S2489	Nateglinide
1272	S2311	Hyodeoxycholic acid (HDCA)
1273	S4600	Benzyl alcohol
1274	S4041	Olsalazine Sodium
1275	S4691	Oxybenzone
1276	S4368	Carbenoxolone Sodium
1277	S4003	Lithocholic acid
1278	S4213	Dirithromycin
1279	S3944	Valproic acid
1280	S4650	Atipamezole
1281	S4304	Anisindione
1282	S4222	Piperacillin Sodium
1283	S3627	Tryptamine
1284	S2566	Isoprenaline HCl
1285	S3850	Glucosamine sulfate
1286	S4070	Guanidine HCl
1287	S3719	Topiroxostat
1288	S3980	Pyridoxine
1289	S2457	Clindamycin HCl
1290	S2511	Sulfadoxine
1291	S4652	Sulisobenzone
1292	S2494	Olopatadine HCl

1293	S4660	Glycopyrrolate
1294	S4850	Flucloxacillin sodium
1295	S4847	Faropenem Sodium
1296	S4570	Halothane
1297	S3037	Bepotastine Besilate
1298	S4004	Ethambutol 2HCl
1299	S3794	Palmitic acid
1300	S4376	Aceclidine HCl
1301	S4131	Levodropropizine
1302	S3985	Methyl 4-hydroxybenzoate
1303	S3638	Cefamandole nafate
1304	S4693	Guanfacine Hydrochloride
1305	S3856	Allantoin
1306	S4655	Sulpiride
1307	S4073	Sodium 4-Aminosalicylate
1308	S3851	Camphor
1309	S4128	Troxipide
1310	S2589	Miglitol
1311	S3971	Fusidine
1312	S2557	Terbinafine HCl
1313	S4649	Atipamezole hydrochloride
1314	S3729	Iron sucrose
1315	S4509	4-Aminoantipyrine
1316	S4516	(+)-Camphor
1317	S4512	Aceglutamide
1318	S4641	Tedizolid Phosphate
1319	S3979	Zinc Undecylenate
1320	S4759	p-Coumaric Acid
1321	S4843	Potassium acetate
1322	S4170	Coumarin
1323	S3950	Maltitol
1324	S4119	Pefloxacin Mesylate Dihydrate
1325	S4546	Xylitol
1326	S4344	Oxprenolol HCl
1327	S3881	Scopoletin
1328	S3909	Catalpol
1329	S4257	Alizapride HCl
1330	S4211	Montelukast Sodium
1331	S2590	Pioglitazone
1332	S3992	D-(+)-Trehalose dihydrate
1333	S4244	Serotonin HCl
1334	S4667	Lidocaine hydrochloride
1335	S4568	Ethylvanillin
1336	S2573	Tetracaine HCl
1337	S4047	Bisacodyl
1338	S3178	Brinzolamide
1339	S4345	Pentoxifylline
1340	S4679	Terazosin HCl
1341	S4539	Salicylic acid
1342	S4308	Benzthiazide
1343	S3754	4-Hydroxybenzoic acid
1344	S4848	Dalbavancin
1345	S2765	MK-2048
1346	S4538	Pantoprazole sodium

1347	S2551	Sulbactam sodium
1348	S4657	Eslicarbazepine Acetate
1349	S4563	Diatrizoic acid
1350	S4532	Iopamidol
1351	S4669	Benzocaine hydrochloride
1352	S4524	2-Ethoxybenzamide
1353	S4382	Pyrilamine Maleate
1354	S2283	Cinchonine(LA40221)
1355	S1504	Dyphylline
1356	S4696	CarbinoxaMine Maleate
1357	S3975	Protocatechuic acid
1358	S4201	Florfenicol
1359	S3612	Rosmarinic acid
1360	S4387	Bendroflumethiazide
1361	S3977	(-)-Borneol
1362	S4813	Cefuroxime axetil
1363	S4090	Fenspiride HCl
1364	S4637	Prasugrel Hydrochloride
1365	S4717	Isatin
1366	S4656	Parecoxib
1367	S3799	Gentisic acid
1368	S4316	Clopamide
1369	S3870	D-Pinitol
1370	S4014	Hyoscyamine
1371	S4697	Saxagliptin hydrate
1372	S4819	Saccharin
1373	S3046	Azilsartan
1374	S4663	Fusidate Sodium
1375	S4626	Ethosuximide
1376	S4365	Phthalylsulfacetamide
1377	S4124	Tolazoline HCl
1378	S4594	Cephalothin
1379	S4247	Prucalopride Succinate
1380	S3750	Sodium benzoate
1381	S4299	Dicoumarol
1382	S2613	Clorsulon
1383	S2116	Conivaptan HCl
1384	S4792	N-Acetylneuraminic acid
1385	S4361	Cinoxacin
1386	S2484	Milrinone
1387	S4595	Cefazolin Sodium
1388	S4338	Methoxamine HCl
1389	S3639	Tacrine hydrochloride hydrate
1390	S4534	6-Acetamidohexanoic acid
1391	S4769	L-5-Hydroxytryptophan
1392	S4530	i-Inositol
1393	S4749	Citalopram HBr
1394	S3008	Zaltoprofen
1395	S4514	Acetylleucine
1396	S3637	Cefpirome sulfate
1397	S4385	Fosfomycin Tromethamine
1398	S4753	Ganoderic acid A
1399	S4678	Povidone iodine
1400	S4388	Bentiromide

1401	S3885	Pyrogallol
1402	S2615	Noradrenaline bitartrate monohydrate
1403	S4515	Ademetionine disulfate tosylate
1404	S4322	Disopyramide Phosphate
1405	S4844	Cefcapene Pivoxil Hydrochloride
1406	S2559	Cortisone acetate
1407	S4625	Alcaftadine
1408	S4618	Fenoldopam mesylate
1409	S4687	Rivastigmine
1410	S4297	Mupirocin
1411	S4716	Evans Blue
1412	S4752	Corticosterone
1413	S4525	Ethylparaben
1414	S4815	L-Cysteine HCl
1415	S4295	Meclofenamate Sodium
1416	S4682	Loxoprofen
1417	S4817	Atenolol
1418	S4722	(+)-Catechin
1419	S3901	Astragaloside IV
1420	S4553	Bronopol
1421	S2851	Baricitinib (LY3009104, INCB028050)
1422	S4279	Demeclocycline HCl
1423	S4723	(-)Epicatechin
1424	S4277	Bambuterol HCl
1425	S4503	Calcium D-Panthotenate
1426	S4278	Carteolol HCl
1427	S4550	Azelaic acid
1428	S4779	Menadiol Diacetate
1429	S4301	(R)-(+)-Atenolol HCl
1430	S4548	Aminoguanidine hydrochloride
1431	S3974	(+)-Catechin hydrate
1432	S4171	Choline Chloride
1433	S4816	Diatrizoate sodium
1434	S2486	Moroxydine HCl
1435	S4564	Diethylcarbamazine citrate
1436	S4267	Diacerein
1437	S4768	Melibiose
1438	S4849	Levocetirizine Dihydrochloride
1439	S4628	(+/-)-Sulfinpyrazone
1440	S4294	Procainamide HCl
1441	S2556	Rosiglitazone
1442	S4735	Salvianolic acid B
1443	S4596	Cefixime
1444	S4726	Lauric Acid
1445	S4832	Tolmetin
1446	S4526	Fenbufen
1447	S4289	Metoclopramide HCl
1448	S4305	Anisotropine Methylbromide
1449	S4599	Benzyl benzoate
1450	S1983	Adenine HCl
1451	S4623	Methylbenactyzine Bromide
1452	S2664	Clinofibrate
1453	S4335	Metaproterenol Sulfate
1454	S4686	Vitamin E

1455	S4629	Chlorotrianisene
1456	S4148	Ampicillin Trihydrate
1457	S3207	Iopromide
1458	S3066	Naloxone HCl
1459	S3057	Azilsartan Medoxomil
1460	S4634	Sodium sulfadiazine
1461	S4630	Diazoxide
1462	S4633	Isosorbide Mononitrate
1463	S4291	Labetalol HCl

Supplementary Table 2. Primers used in qRT-PCR

Primer	Sequence (5’ to 3’)
GAPDH-Forward	TGGTATCGTGGAAGGACTCA
GAPDH-Reverse	CCAGTAGAGGCAGGGATGAT
AXIN2-Forward	CCTGGGGGCAGCGAGTATTA
AXIN2-Reverse	GCTTTGGGCACCAAACTGCT
TNFAIP3-Forward	GCCAAGAGAGATCACACCCC
TNFAIP3-Reverse	GCGATCCTTTCGCAAAGTCC
BIRC3-Forward	CGACAGGTGTCGCTTGAAAA
BIRC3-Reverse	TCCTTTGTAAACCTGCTGCC
NFκB1A-Forward	GGGCCAGCTGACACTAGAAA
NFκB1A-Reverse	GTCATCATAGGGCAGCTCGT
c-JUN-Forward	TGCTCTGGGAAGTGAGTTCG
c-JUN-Reverse	TGAAAAGTCGCGGTCACTCA
CD44-Forward	GCAAACACAACCTCTGGTCC
CD44-Reverse	CCCACACCTTCTTCGACTGT
c-myc-Forward	CCCTCCACTCGGAAGGACTA
c-myc-Reverse	GCTGGTGCATTTTCGGTTGT
Survivin-Forward	CACTGCTGTGGACCCTACTG
Survivin-Reverse	AGAGACAACTGCGTCTCTGC

Supplementary Table 3. Univariate and multivariate analysis of prognostic factors associated with OS in all 41 HCC patients with sorafenib.

HCC patients with sorafenib	Number			Multivariate Analysis	
(n=41)		HR(95% CI)	*p*-value	HR(95% CI)	*p*-value
**Sex**					
male	33				
female	8	**0.7(0.32,1.53)**	**0.371**		
**HBV**					
No	11				
Yes	33	**0.63 (0.31,1.32)**	**0.221**		
**Liver cirrhosis**					
No	16				
Yes	25	**0.84 (0.45,1.60)**	**0.606**		
**Pre-medication AFP(ng/ml)**					
≦20	17				
＞20	24	**1.3 (0.68,2.5)**	**0.433**		
**TBIL (μmol/mL)**					
≦19	15				
＞19	26	**0.55(0.28,1.08)**	**0.084**	**0.54(0.27,1.06)**	**0.074**
**ALT (U/L)**					
≦50	20				
＞50	21	**1.04(0.56,1.96)**	**0.894**		
**AST (U/L)**					
≦40	18				
＞40	23	**1.59 (0.84, 3)**	**0.154**		
**Tumor size**					
≦5	9				
＞5	13	**0.58 (0.24, 1.44)**	**0.24**		
**Tumor number**					
＞1	14				
≦1	17	**0.57 (0.26, 1.22)**	**0.145**		
**Vascular invasion**					
No	32				
Yes	9	**0.72 (0.32, 1.59)**	**0.411**		
**Lymph nodes metastasis**					
No	33				
Yes	8	**1.23 (0.56, 2.69)**	**0.609**		
**Distant Metastasis**					
No	31				
Yes	10	**1.37 (0.69, 2.75)**	**0.369**		
**Accompanied by TACE**					
Yes	10				
No	31	**1.68 (0.81, 3.48)**	**0.167**		
**Staining of overall β-catenin**					
Low	13				
High	28	**1.09 (0.55, 2.16)**	**0.794**		
**Staining of cytosolic β-catenin**					
Low	13				
High	28	**1.09 (0.55, 2.16)**	**0.794**		
**Staining of nuclear β-catenin**					
Negative	29				
Positive	12	**6.46 (2.76, 15.09)**	**1.67E-05**	**6.62(2.81,1.56)**	**<0.001**

Supplementary Table 4. The cell viability of candidate compounds treatment in MHCC-97H cells, related to supplementary figure 2

Cat No.	Drug name	Target	Pathway	cell viability (sorafenib+ drugs) %	The reduction of cell viability (sorafenib +drugs vs sorafenib)%
S8968	PRI-724	CBP/β-catenin	Wnt/β-catenin	15.99%	60.86%
S1393	Pirarubicin	Topoisomerase	Topoisomerase	16.06%	60.79%
S2481	Manidipine	Calcium Channel	Calcium	16.12%	60.73%
S1302	Ifosfamide	DNA/RNA Synthesis	DNA/RNA	16.18%	60.67%
S1042	Sunitinib Malate	c-Kit,PDGFR,VEGFR	c-Kit,PDGFR,VE	16.38%	60.47%
S1787	Teniposide	Topoisomerase	Topoisomerase	16.44%	60.41%
S2456	Chlorpromazine HCl	Potassium Channel,Dopamine Receptor	Potassium Channel,Dopamine Receptor	16.44%	60.41%
S1351	Ivermectin	Chloride Channel,Anti-infection	Chloride Channel,Anti-infection	17.68%	59.17%
S1432	Sumatriptan	5-HT Receptor	5-HT Receptor	17.93%	58.92%
S2031	Fenticonazole	Anti-infection	Anti-infection	18.06%	58.79%
S2382	Evodiamine	NF-κB	NF-κB	18.13%	58.72%
S4631	Prochlorperazine dimaleate salt	Dopamine Receptor	Dopamine Receptor	18.19%	58.66%
S3611	(+)-Fangchinoline	Others	Others	18.45%	58.40%
S1305	Mercaptopurine (6-	DNA/RNA Synthesis	DNA/RNA	18.58%	58.27%
S2098	Bexarotene	Retinoid Receptor	Retinoid	18.97%	57.88%
S1053	Entinostat (MS-	HDAC	HDAC	19.04%	57.81%
S4107	Clofazimine	Phospholipase (e.g. PLA)	Phospholipase (e.g. PLA)	19.56%	57.29%
S1550	Pimobendan	PDE	PDE	19.88%	56.97%
S1640	Albendazole	Microtubule Associated	Microtubule Associated	20.07%	56.78%
S2077	Atorvastatin Calcium	HMG-CoA Reductase	HMG-CoA Reductase	20.20%	56.65%
S1207	Tivozanib (AV-951)	c-Kit,PDGFR,VEGFR	c-Kit,PDGFR,VE	20.27%	56.58%
S4589	Amodiaquine dihydrochloride dihydrate	Transferase,Histone Methyltransferase	Transferase,Histone Methyltransfera	20.53%	56.32%
S1485	HMN-214	PLK	PLK	20.66%	56.19%
S1771	Chlorprothixene	Dopamine Receptor	Dopamine	20.79%	56.06%
S3698	Nortriptyline hydrochloride	Others	Others	20.85%	56.00%
S1178	Regorafenib (BAY 73-4506)	c-RET,VEGFR	c-RET,VEGFR	20.98%	55.87%
S1453	Tipifarnib	Transferase	Transferase	21.11%	55.74%
S1831	Carvedilol	Adrenergic Receptor	Adrenergic Receptor	21.24%	55.61%
S1526	Quizartinib	FLT3	FLT3	21.37%	55.48%
S1390	Ondansetron HCl	5-HT Receptor	5-HT Receptor	21.43%	55.42%
S1144	Ivacaftor (VX-770)	CFTR	CFTR	21.50%	55.35%
S1189	Aprepitant	Substance P	Substance P	21.63%	55.22%
S2517	Maprotiline HCl	Adrenergic Receptor	Adrenergic Receptor	21.95%	54.90%
S4282	Nelfinavir Mesylate	HIV Protease	HIV Protease	22.02%	54.83%

S1241	Vincristine sulfate	Autophagy,Microtubule Associated	Autophagy,Microtubule	22.28%	54.57%
S2284	Colchicine	Microtubule Associated	Microtubule Associated	22.34%	54.51%
S4505	Vinblastine sulfate	Microtubule Associated,AChR	Microtubule Associated,AC	22.60%	54.25%
S4269	Vinorelbine Tartrate	Microtubule Associated	Microtubule Associated	22.67%	54.18%
S4296	Salmeterol Xinafoate	Adrenergic Receptor	Adrenergic Receptor	22.80%	54.05%
S1792	Simvastatin	HMG-CoA Reductase	HMG-CoA Reductase	22.86%	53.99%
S1257	Posaconazole	P450 (e.g. CYP17)	P450 (e.g.	23.12%	53.73%
S4084	Lomerizine 2HCl	Calcium Channel	Calcium	23.25%	53.60%
S3019	Ciclopirox	ATPase	ATPase	23.38%	53.47%
S2528	Ciclopirox	ATPase,Anti-infection	ATPase,Anti-infection	23.44%	53.41%
S1776	Toremifene Citrate	Estrogen/progestogen Receptor	Estrogen/progestogen	23.57%	53.28%
S2028	Diclazuril	Anti-infection	Anti-infection	23.64%	53.21%
S1848	Curcumin	NF-κB,HDAC,Histone Acetyltransferase,Nrf2	NF-κB,HDAC,Histone Acetyltransfera	23.70%	53.15%
S4783	Benzyl	Others	Others	24.35%	52.50%
S4541	Triclosan	Anti-infection	Anti-infection	24.55%	52.30%
S4619	Itopride	AChR	AChR	24.61%	52.24%
S4223	Mevastatin	HMG-CoA Reductase	HMG-CoA Reductase	24.87%	51.98%
S3030	Niclosamide	STAT	STAT	24.87%	51.98%
S4406	Pindolol	Others	Others	25.00%	51.85%
S2075	Rosiglitazone HCl	PPAR	PPAR	25.00%	51.85%
S2217	Irinotecan HCl Trihydrate	Topoisomerase	Topoisomerase	25.00%	51.85%
S3676	Carbendazim	Anti-infection	Anti-infection	25.13%	51.72%
S3022	Cabazitaxel	Microtubule Associated	Microtubule Associated	25.19%	51.66%
S2334	Oleanolic Acid	Anti-infection	Anti-infection	25.39%	51.46%
S1333	Fluoxetine HCl	5-HT Receptor（选择性5-HT再摄取抑制剂（SSRI）	5-HT Receptor（选择性5-HT再摄取抑制剂	26.04%	50.81%
S4000	Pergolide Mesylate	Dopamine Receptor	Dopamine	26.10%	50.75%
S2625	Fostamatinib	Syk	Syk	26.36%	50.49%
S4182	Nifuroxazide	STAT	STAT	26.49%	50.36%
S1120	Everolimus	mTOR	mTOR	26.81%	50.04%
S3689	Ethidium bromide	Others	Others	26.81%	50.04%
S2475	Imatinib (STI571)	PDGFR	PDGFR	27.40%	49.45%
S1190	Bicalutamide	Androgen Receptor	Androgen	27.66%	49.19%
S3671	Quinestrol	Estrogen/progestogen Receptor	Estrogen/progestogen	27.72%	49.13%
S2814	Alpelisib (BYL719)	PI3K	PI3K	27.98%	48.87%
S1721	Azathioprine	Rho	Rho	28.11%	48.74%
S2151	Sonidegib (Erismodegib,	Hedgehog/Smoothened	Hedgehog/Smoothened	28.11%	48.74%
S1979	Amiodarone HCl	Autophagy,Potassium Channel	Autophagy,Potassium	28.24%	48.61%
S3060	Medetomidine HCl	Adrenergic Receptor	Adrenergic Receptor	28.31%	48.54%
S2290	Dihydroartemisinin (DHA)	Others	Others	28.50%	48.35%

S1830	Oxfendazole	Anti-infection	Anti-infection	29.21%	47.64%
S4597	Lercanidipine hydrochloride	Calcium Channel	Calcium Channel	29.21%	47.64%
S1021	Dasatinib	Bcr-Abl,c-Kit,Src	Bcr-Abl,c-	29.28%	47.57%
S3047	Otilonium Bromide	AChR	AChR	29.28%	47.57%
S4394	Clofoctol	Others	Others	29.28%	47.57%
S2680	Ibrutinib (PCI-	BTK	BTK	29.34%	47.51%
S2017	Benidipine HCl	Calcium Channel	Calcium	29.34%	47.51%
S1775	Thiotepa	Others	Others	29.41%	47.44%
S4007	Pentamidine	phosphatase	phosphatase	29.73%	47.12%
S2762	Alectinib	ALK	ALK	29.80%	47.05%
S2348	Rotenone	Anti-infection	Anti-infection	29.93%	46.92%
S1869	Dapoxetine HCl	5-HT Receptor（5-羟色胺再吸收抑制剂（SSRI））	5-HT Receptor（5-羟色胺再吸收抑制剂	30.06%	46.79%
S2536	Miconazole	Anti-infection	Anti-infection	30.06%	46.79%
S2541	Clomipramine HCl	5-HT Receptor（阻断 5-HT 再摄取）	5-HT Receptor（阻断 5-HT 再	30.18%	46.67%
S1328	Etodolac	COX	COX	30.25%	46.60%
S1497	Pralatrexate	DHFR	DHFR	30.38%	46.47%
S3817	Harmine	DYRK	DYRK	30.96%	45.89%
S1625	Ethinyl Estradiol	Estrogen/progestogen Receptor	Estrogen/progestogen	31.09%	45.76%
S3914	α-Hederin	Others	Others	31.35%	45.50%
S1148	Docetaxel	Microtubule Associated	Microtubule Associated	31.35%	45.50%
S2552	Azelastine HCl	Histamine Receptor	Histamine	31.87%	44.98%
S1714	Gemcitabine	Autophagy,DNA/RNA Synthesis	Autophagy,DNA/RNA	31.93%	44.92%
S1267	Vemurafenib (PLX4032,	Raf	Raf	31.93%	44.92%
S2030	Flunarizine 2HCl	Calcium Channel	Calcium	32.06%	44.79%
S4504	6-Mercaptopurine (6-MP)	DNA/RNA Synthesis	DNA/RNA Synthesis	32.13%	44.72%
S1150	Paclitaxel	Autophagy,Microtubule Associated	Autophagy,Microtubule	32.19%	44.66%
S4354	Thiostrepton	Others	Others	32.26%	44.59%
S1210	Methotrexate	DHFR	DHFR	32.45%	44.40%
S1035	Pazopanib HCl (GW786034 HCl)	c-Kit,PDGFR,VEGFR	c-Kit,PDGFR,VE	32.45%	44.40%
S1949	Menadione	DNA/RNA Synthesis,phosphatase	DNA/RNA Synthesis,phosphatase	32.58%	44.27%
S1289	Carmofur	DNA/RNA Synthesis	DNA/RNA	32.65%	44.20%
S2303	gossypol-Acetic	Dehydrogenase	Dehydrogenas	32.71%	44.14%
S1225	Etoposide	Topoisomerase	Topoisomerase	32.84%	44.01%
S1330	Felbamate	NMDAR	NMDAR	33.04%	43.81%
S3883	Protopine	Calcium Channel	Calcium	33.23%	43.62%
S3692	N-Ethylmaleimide (NEM)	Cysteine Protease	Cysteine Protease	33.23%	43.62%
S3738	Travoprost	Others	Others	33.56%	43.29%
S4237	Primaquine Diphosphate	Anti-infection	Anti-infection	33.88%	42.97%
S1378	Ruxolitinib (INCB018424)	JAK	JAK	33.88%	42.97%
S2226	Idelalisib (CAL-101, GS-1101)	PI3K	PI3K	33.94%	42.91%
S1199	Cladribine	DNA/RNA Synthesis	DNA/RNA	34.20%	42.65%
S2321	Magnolol	NF-κB	NF-κB	34.53%	42.32%

S1098	Rucaparib (AG-014699,PF-01367338)	PARP	PARP	34.66%	42.19%
S1994	Lacidipine	Calcium Channel	Calcium	34.66%	42.19%
S1026	Imatinib Mesylate (STI571)	Bcr-Abl,c-Kit,PDGFR	Bcr-Abl,c-Kit,PDGFR	34.79%	42.06%
S3713	Moxidectin	Anti-infection	Anti-infection	34.85%	42.00%
S2246	Abiraterone	P450 (e.g. CYP17)	P450 (e.g.	34.92%	41.93%
S1491	Fludarabine	DNA/RNA Synthesis,STAT	DNA/RNA Synthesis,STA	35.56%	41.29%
S1813	Amlodipine	Calcium Channel	Calcium	35.69%	41.16%
S1218	Clofarabine	DNA/RNA Synthesis	DNA/RNA	35.82%	41.03%
S1229	Fludarabine	DNA/RNA Synthesis	DNA/RNA	35.82%	41.03%
S2001	Elvitegravir (GS-9137, JTK-303)	Integrase	Integrase	35.95%	40.90%
S1644	Nitrofural	Anti-infection	Anti-infection	36.15%	40.70%
S3161	Sertaconazole	Anti-infection	Anti-infection	36.34%	40.51%
S3868	Harmine	PPAR,MAO	PPAR,MAO	36.47%	40.38%
S3643	Amitraz	Adrenergic Receptor	Adrenergic Receptor	36.47%	40.38%
S1319	Costunolide	Telomerase	Telomerase	36.67%	40.18%
S1973	Cyclocytidine HCl	DNA/RNA Synthesis	DNA/RNA	36.86%	39.99%
S1987	Mometasone furoate	Glucocorticoid Receptor	Glucocorticoid Receptor	36.99%	39.86%
S1613	Silodosin	Adrenergic Receptor	Adrenergic Receptor	37.06%	39.79%
S4285	Ospemifene	Estrogen/progestogen Receptor	Estrogen/progestogen	37.51%	39.34%
S2797	Lonafarnib	Transferase	Transferase	37.51%	39.34%
S3183	Amitriptyline HCl	5-HT Receptor（5-HT4、5-HT2 和 sigma 1 受体的抑制剂）	5-HT Receptor（5-HT4、5-HT2 和 sigma 1 受体的抑制	37.64%	39.21%
S4283	Cyclobenzaprine HCl	5-HT Receptor（拮抗 ADRA2、5-HT2A 受体和 H1 受体）	5-HT Receptor（拮抗 ADRA2、5-HT2A 受体	37.70%	39.15%
S3966	Nifuratel	Antibacterial	Antibacterial	37.90%	38.95%
S1025	Gefitinib (ZD1839)	EGFR	EGFR	38.09%	38.76%
S1514	Cyclosporine	phosphatase,Immunology & Inflammation related	phosphatase,Immunology & Inflammation	38.16%	38.69%
S1044	Temsirolimus (CCI-779, NSC 683864)	mTOR	mTOR	38.61%	38.24%
S3621	Pazufloxacin	Anti-infection	Anti-infection	38.68%	38.17%
S1833	Butoconazole	Anti-infection	Anti-infection	38.68%	38.17%
S2037	Candesartan	RAAS	RAAS	38.81%	38.04%
S2286	Cyclosporin A	Immunology & Inflammation related	Immunology & Inflammation	38.87%	37.98%
S1501	Mycophenolate	Dehydrogenase	Dehydrogenas	39.26%	37.59%
S1836	Albendazole Oxide	Microtubule Associated	Microtubule Associated	39.32%	37.53%
S1610	Metolazone	Others	Others	39.39%	37.46%
S4286	Anidulafungin (LY303366)	Anti-infection	Anti-infection	39.65%	37.20%
S1200	Decitabine	DNA Methyltransferase	DNA Methyltransfera	39.71%	37.14%
S2188	Phenprocoumon	5-alpha Reductase	5-alpha	39.78%	37.07%
S2265	Artesunate	STAT	STAT	39.84%	37.01%
S4646	Ciclesonide	Others	Others	39.91%	36.94%
S1880	Roxatidine Acetate	Histamine Receptor	Histamine	39.97%	36.88%

S1956	Miconazole Nitrate	Anti-infection	Anti-infection	40.10%	36.75%
S1959	Tolfenamic Acid	COX	COX	40.10%	36.75%
S4011	Ampiroxicam	COX	COX	40.23%	36.62%
S1293	Cilnidipine	Calcium Channel	Calcium	40.30%	36.55%
S1733	Methylprednisolone	Glucocorticoid Receptor,Immunology & Inflammation related	Glucocorticoid Receptor,Immunology & Inflammation	40.36%	36.49%
S4228	Fluorometholone Acetate	Glucocorticoid Receptor	Glucocorticoid Receptor	40.36%	36.49%
S1712	Deferasirox	P450 (e.g. CYP17)	P450 (e.g.	40.43%	36.42%
S2006	Pyrimethamine	DHFR	DHFR	40.75%	36.10%
S1299	Floxuridine	DNA/RNA Synthesis	DNA/RNA	40.75%	36.10%
S3747	Levothyroxine	Others	Others	41.33%	35.52%
S2169	Rosuvastatin Calcium	HMG-CoA Reductase	HMG-CoA Reductase	41.40%	35.45%
S1443	Zileuton	Lipoxygenase	Lipoxygenase	41.46%	35.39%
S1652	Monobenzone	Tyrosinase	Tyrosinase	41.59%	35.26%
S1008	Selumetinib	MEK	MEK	41.72%	35.13%
S4012	Desloratadine	Histamine Receptor	Histamine	41.79%	35.06%
S1060	Olaparib (AZD2281, Ku-	PARP	PARP	41.98%	34.87%
S1782	Azacitidine	DNA Methyltransferase	DNA Methyltransfera	42.11%	34.74%
S1156	Capecitabine	DNA/RNA Synthesis	DNA/RNA	42.11%	34.74%
S3080	Etravirine (TMC125)	Reverse Transcriptase	Reverse Transcriptase	42.18%	34.67%
S3618	Acetylspiramycin (ASPM)	Anti-infection	Anti-infection	42.18%	34.67%
S1905	Amlodipine	Calcium Channel	Calcium	42.24%	34.61%
S4120	Sulconazole Nitrate	Anti-infection	Anti-infection	42.24%	34.61%
S1426	Repaglinide	Potassium Channel	Potassium	42.37%	34.48%
S4221	Benzbromarone	P450 (e.g. CYP17)	P450 (e.g.	42.43%	34.42%
S4690	Escin	Immunology & Inflammation related	Immunology & Inflammation	42.50%	34.35%
S4674	Hydroxyprogesterone caproate	Estrogen/progestogen Receptor	Estrogen/progestogen	42.50%	34.35%
S1137	Malotilate	Others	Others	42.50%	34.35%
S1483	Iloperidone	5-HT Receptor（5-HT2 受体拮抗剂，5-羟色胺2A受体拮抗剂）	5-HT Receptor（5-HT2 受体拮抗剂，5-羟色胺2A受体拮抗	42.69%	34.16%
S1431	Sildenafil Citrate	PDE	PDE	42.76%	34.09%
S3895	Sophoridine	Others	Others	42.95%	33.90%
S1227	Raloxifene HCl	Estrogen/progestogen Receptor	Estrogen/progestogen	43.02%	33.83%
S3876	Indigo	Others	Others	43.34%	33.51%
S2126	Naftopidil	Adrenergic Receptor	Adrenergic Receptor	43.34%	33.51%
S4181	Nicardipine HCl	Calcium Channel	Calcium	43.41%	33.44%
S1403	Tigecycline	Anti-infection	Anti-infection	43.47%	33.38%
S3189	Ropinirole HCl	Dopamine Receptor	Dopamine	43.54%	33.31%
S3722	Isavuconazole	Anti-infection	Anti-infection	43.80%	33.05%
S1885	Felodipine	Calcium Channel	Calcium	43.86%	32.99%
S2125	Mestranol	Estrogen/progestogen Receptor	Estrogen/progestogen	43.93%	32.92%
S1468	Alfacalcidol	Vitamin	Vitamin	43.99%	32.86%
S3038	Fosaprepitant dimeglumine salt	Others	Others	44.06%	32.79%
S2741	Niraparib (MK-	PARP	PARP	44.12%	32.73%

S2229	Eltrombopag	Thrombin	Thrombin	44.12%	32.73%
S1276	Adapalene	Retinoid Receptor	Retinoid	44.18%	32.67%
S1778	Trifluridine	DNA/RNA Synthesis	DNA/RNA	44.25%	32.60%
S3163	Benztropine	Dopamine Receptor	Dopamine	44.25%	32.60%
S1214	Bleomycin sulfate	DNA/RNA Synthesis	DNA/RNA	44.31%	32.54%
S3872	Guaiacol	Others	Others	44.38%	32.47%
S1386	Nafamostat	Serine Protease	Serine	44.38%	32.47%
S2062	Tiopronin	ROS	ROS	44.51%	32.34%
S1601	Reserpine	Others	Others	44.83%	32.02%
S2310	Honokiol	Akt,MEK	Akt,MEK	44.83%	32.02%
S1022	Ridaforolimus (Deforolimus, MK-	mTOR	mTOR	45.09%	31.76%
S1202	Dutasteride	5-alpha Reductase	5-alpha	45.16%	31.69%
S2603	Tioxolone	Carbonic Anhydrase	Carbonic	45.22%	31.63%
S1914	Pregnenolone	Estrogen/progestoge n Receptor	Estrogen/proge stogen	45.22%	31.63%
S1430	Rolipram	PDE	PDE	45.29%	31.56%
S4080	Triamterene	Sodium Channel	Sodium	45.48%	31.37%
S1883	Idoxuridine	Anti-infection	Anti-infection	45.48%	31.37%
S3149	Estradiol valerate	Estrogen/progestoge n Receptor	Estrogen/proge stogen	45.61%	31.24%
S2487	Mycophenolic acid	Dehydrogenase	Dehydrogenas	45.68%	31.17%
S3706	Sarpogrelate hydrochloride	5-HT Receptor（5-HT2R 拮抗剂，5-HT2A，5-HT2B，和 5-HT2C 受体。）	5-HT Receptor （5-HT2R 拮抗剂，5-HT2A，5-HT2B，和 5-	45.81%	31.04%
S1457	Atazanavir Sulfate	HIV Protease	HIV Protease	45.93%	30.92%
S3144	Darifenacin HBr	AChR	AChR	46.00%	30.85%
S2470	Fluocinolone Acetonide	Glucocorticoid Receptor	Glucocorticoid Receptor	46.19%	30.66%
S2515	Vardenafil HCl Trihydrate	PDE	PDE	46.26%	30.59%
S1718	Adefovir Dipivoxil	Reverse Transcriptase	Reverse Transcriptase	46.26%	30.59%
S2673	Trametinib (GSK1120212)	MEK	MEK	46.39%	30.46%
S2606	Mifepristone	Estrogen/progestoge n Receptor	Estrogen/proge stogen	46.39%	30.46%
S1839	Chloroxine	Anti-infection	Anti-infection	46.78%	30.07%
S1387	Naftopidil DiHCl	Adrenergic Receptor	Adrenergic Receptor	46.84%	30.01%
S1191	Fulvestrant	Estrogen/progestoge n Receptor	Estrogen/proge stogen	46.84%	30.01%
S4552	Bithionol	cAMP	cAMP	47.04%	29.81%
S1465	Moxifloxacin HCl	Topoisomerase	Topoisomerase	47.04%	29.81%
S1854	Bifonazole	Anti-infection	Anti-infection	47.10%	29.75%
S1394	Pizotifen Malate	5-HT Receptor（5-HT2A ）	5-HT Receptor （5-HT2A ）	47.23%	29.62%
S1467	Doxercalciferol	Vitamin	Vitamin	47.23%	29.62%
S1992	Fluticasone propionate	Glucocorticoid Receptor	Glucocorticoid Receptor	47.23%	29.62%
S1185	Ritonavir	HIV Protease,P450 (e.g. CYP17)	HIV Protease,P450	47.30%	29.55%
S3951	Tannic acid	CXCR	CXCR	47.49%	29.36%
S3716	Flibanserin	5-HT Receptor （2A）	5-HT Receptor （2A）	47.49%	29.36%
S4518	Chloroxylenol	Anti-infection	Anti-infection	47.75%	29.10%
S3000	Carbazochrome sodium sulfonate	Others	Others	47.88%	28.97%
S4056	Retapamulin	Anti-infection	Anti-infection	48.14%	28.71%

S1458	VX-745	p38 MAPK	p38 MAPK	48.14%	28.71%
S2567	Medroxyprogesterone acetate	Estrogen/progestogen Receptor	Estrogen/progestogen	48.14%	28.71%
S1547	Febuxostat	ROS	ROS	48.46%	28.39%
S1604	Olmesartan	RAAS	RAAS	48.53%	28.32%
S4259	Vilazodone HCl	5-HT Receptor（5-羟色胺1A受体激动剂）	5-HT Receptor（5-羟色胺1A受体激动剂）	48.66%	28.19%
S1082	Vismodegib (GDC-0449)	Hedgehog/Smoothened	Hedgehog/Smoothened	48.66%	28.19%
S1283	Asenapine maleate	5-HT Receptor,Adrenergic Receptor（5-HT2A）	5-HT Receptor,Adrenergic Receptor（5-	48.79%	28.06%
S4059	Sodium Nitroprusside	Others	Others	48.98%	27.87%
S1055	Enzastaurin	PKC	PKC	49.11%	27.74%
S1435	Tamsulosin hydrochloride	alpha1 adrenoreceptor	alpha1 adrenoreceptor	49.11%	27.74%
S1284	Benazepril HCl	RAAS	RAAS	49.50%	27.35%
S4381	Proadifen HCl	Others	Others	49.50%	27.35%
S1505	Aztreonam	Anti-infection	Anti-infection	49.50%	27.35%
S2354	Sclareol	Others	Others	49.56%	27.29%
S3959	(+)-Borneol	Others	Others	49.69%	27.16%
S1676	Amorolfine HCl	Anti-infection	Anti-infection	49.69%	27.16%
S1007	Roxadustat (FG-	HIF	HIF	49.76%	27.09%
S1735	Mesna	Others	Others	49.82%	27.03%
S2041	Dyclonine HCl	Sodium Channel	Sodium	49.82%	27.03%
S1322	Dexamethasone (DHAP)	Autophagy,IL Receptor	Autophagy,IL Receptor	49.89%	26.96%
S1920	Haloperidol	Dopamine Receptor	Dopamine	50.02%	26.83%
S1368	Acitretin	Retinoid Receptor	Retinoid	50.15%	26.70%
S1466	Calcitriol	Vitamin	Vitamin	50.15%	26.70%
S1358	Loratadine	Histamine Receptor	Histamine	50.21%	26.64%
S2261	Andrographolide	NF-κB	NF-κB	50.34%	26.51%
S4601	Clioquinol	Anti-infection	Anti-infection	50.60%	26.25%
S1593	Apixaban	Factor Xa	Factor Xa	50.67%	26.18%
S1773	Oxytetracycline (Terramycin)	Anti-infection	Anti-infection	50.80%	26.05%
S1859	Diethylstilbestrol	Estrogen/progestogen Receptor	Estrogen/progestogen	50.86%	25.99%
S1472	Safinamide	MAO	MAO	50.93%	25.92%
S4585	Succinylsulfathiazol	Anti-infection	Anti-infection	51.12%	25.73%
S1685	Sulfanilamide	Anti-infection	Anti-infection	51.12%	25.73%
S1192	Raltitrexed	DNA/RNA Synthesis	DNA/RNA	51.18%	25.67%
S4054	Spironolactone	Androgen Receptor	Androgen	51.18%	25.67%
S2459	Clozapine	5-HT Receptor（5HT2A）	5-HT Receptor（5HT2A）	51.51%	25.34%
S1895	Dipyridamole	PDE	PDE	51.57%	25.28%
S2584	Clobetasol propionate	Glucocorticoid Receptor	Glucocorticoid Receptor	51.70%	25.15%
S3674	Levamlodipine	Calcium Channel	Calcium	51.96%	24.89%
S4751	Cisapride hydrate	5-HT Receptor（选择性5-HT4 receptor激动剂）	5-HT Receptor（选择性5-HT4 receptor激动	52.03%	24.82%
S4079	Ticagrelor	P2 Receptor	P2 Receptor	52.03%	24.82%
S1627	Nitazoxanide	Others	Others	52.03%	24.82%
S4072	Decamethonium Bromide	AChR	AChR	52.09%	24.76%
S1832	Atracurium	AChR	AChR	52.09%	24.76%

S3003	Prostaglandin E2 (PGE2)	Others	Others	52.35%	24.50%
S3081	Ulipristal Acetate (CDB 2914)	Estrogen/progestogen Receptor	Estrogen/progestogen	52.42%	24.43%
S1669	Loteprednol etabonate	Glucocorticoid Receptor	Glucocorticoid Receptor	52.68%	24.17%
S3899	Hederagenin	Others	Others	52.68%	24.17%
S1974	Meglumine	Others	Others	52.74%	24.11%
S1233	2-Methoxyestradiol (2-MeOE2)	HIF	HIF	52.93%	23.92%
S2422	Ipriflavone	Others	Others	53.00%	23.85%
S1499	Cefaclor	Anti-infection	Anti-infection	53.06%	23.79%
S1209	Fluorouracil (5-Fluoracil, 5-FU)	DNA/RNA Synthesis	DNA/RNA Synthesis	53.19%	23.66%
S4343	Oxethazaine	Others	Others	53.19%	23.66%
S1743	NEXIUM (esomeprazole	Proton Pump	Proton Pump	53.19%	23.66%
S2473	Hexestrol	Estrogen/progestogen Receptor	Estrogen/progestogen	53.26%	23.59%
S3728	Grazoprevir	HCV Protease	HCV Protease	53.39%	23.46%
S1649	Zolmitriptan	5-HT Receptor（5-HT1B/1D 受体部分激动剂）	5-HT Receptor（5-HT1B/1D 受体部分激动	53.45%	23.40%
S2534	Isoconazole nitrate	Anti-infection	Anti-infection	53.45%	23.40%
S2040	Nimesulide	COX	COX	53.52%	23.33%
S1673	Aminophylline	PDE	PDE	53.71%	23.14%
S1005	Axitinib	c-Kit,PDGFR,VEGFR	c-Kit,PDGFR,VE	53.78%	23.07%
S2295	Emodin	Dehydrogenase	Dehydrogenas	53.78%	23.07%
S1688	Betamethasone Dipropionate	Glucocorticoid Receptor	Glucocorticoid Receptor	53.84%	23.01%
S1425	Ranolazine 2HCl	Calcium Channel	Calcium	53.91%	22.94%
S1794	Fenofibrate	PPAR	PPAR	53.97%	22.88%
S4609	Diflunisal	COX	COX	53.97%	22.88%
S4274	Rotigotine	Dopamine Receptor	Dopamine	54.04%	22.81%
S2535	Econazole nitrate	Anti-infection,Calcium Channel	Anti-infection,Calcium Channel	54.04%	22.81%
S2043	Memantine HCl	P450 (e.g. CYP17),AMPA Receptor-kainate Receptor-NMDA Receptor	P450 (e.g. CYP17),AMPA Receptor-kainate Receptor-	54.04%	22.81%
S1763	Quetiapine	Dopamine Receptor	Dopamine	54.10%	22.75%
S1754	Oxybutynin	AChR	AChR	54.10%	22.75%
S2064	Balofloxacin	Topoisomerase	Topoisomerase	54.30%	22.55%
S1646	Ketorolac	COX	COX	54.43%	22.42%
S3012	Pazopanib	c-Kit,PDGFR,VEGFR	c-Kit,PDGFR,VE	54.56%	22.29%
S2500	Propafenone HCl	Sodium Channel	Sodium	54.62%	22.23%
S4078	Mefenamic Acid	COX	COX	54.75%	22.10%
S2118	Ibutilide Fumarate	Sodium Channel	Sodium	54.75%	22.10%
S1805	Acetylcholine	AChR	AChR	54.94%	21.91%
S1738	Telmisartan	RAAS	RAAS	54.94%	21.91%
S2721	Nilvadipine	Calcium Channel	Calcium	55.14%	21.71%
S2087	Rivastigmine	AChR	AChR	55.14%	21.71%
S1799	Ranolazine	Calcium Channel	Calcium	55.33%	21.52%
S1569	Tazarotene	Retinoid Receptor	Retinoid	55.33%	21.52%
S2066	Moxonidine	Others	Others	55.33%	21.52%

S1376	Gestodene	Estrogen/progestogen Receptor	Estrogen/progestogen	55.40%	21.45%
S2922	Icotinib	EGFR	EGFR	55.46%	21.39%
S1039	Rapamycin	Autophagy,mTOR	Autophagy,mT	55.59%	21.26%
S1278	Altretamine	DNA alkylator	DNA alkylator	55.59%	21.26%
S3002	Rivaroxaban	Factor Xa	Factor Xa	55.59%	21.26%
S2067	Ozagrel HCl	P450 (e.g. CYP17)	P450 (e.g.	55.72%	21.13%
S1910	Tioconazole	Anti-infection	Anti-infection	55.79%	21.06%
S3746	Lumefantrine	Anti-infection	Anti-infection	55.85%	21.00%
S4416	Trimipramine	Others	Others	56.11%	20.74%
S3024	Lamotrigine	Sodium Channel,5-HT Receptor	Sodium Channel,5-HT	56.24%	20.61%
S2332	Neohesperidin	ROS	ROS	56.24%	20.61%
S2044	Cyproheptadine	Histamine Receptor	Histamine	56.31%	20.54%
S2365	Tanshinone IIA	Lipase	Lipase	56.31%	20.54%
S1760	Rifapentine	DNA/RNA Synthesis	DNA/RNA	56.37%	20.48%
S1620	Darunavir	HIV Protease	HIV Protease	56.43%	20.42%
S1286	Budesonide	Glucocorticoid Receptor	Glucocorticoid Receptor	56.56%	20.29%
S4227	Fidaxomicin	DNA/RNA Synthesis	DNA/RNA	56.63%	20.22%
S2450	Equol	Estrogen/progestogen Receptor	Estrogen/progestogen	56.69%	20.16%
S1382	Mianserin HCl	Histamine Receptor	Histamine	56.69%	20.16%
S2271	Berberine chloride	Anti-infection	Anti-infection	56.82%	20.03%
S1835	Azithromycin	Anti-infection,Autophagy	Anti-infection,Autop	56.89%	19.96%
S2318	Lappaconitine	Sodium Channel	Sodium	56.89%	19.96%
S1380	Lopinavir	HIV Protease	HIV Protease	56.89%	19.96%
S4602	Acetohydroxamic	Anti-infection	Anti-infection	56.95%	19.90%
S4207	Clofibric Acid	PPAR	PPAR	57.02%	19.83%
S2358	Silymarin	Others	Others	57.02%	19.83%
S1488	Naratriptan HCl	5-HT Receptor（5-HT1B/1D受体激动剂）	5-HT Receptor （5-HT1B/1D受体激动剂）	57.08%	19.77%
S1808	Nifedipine	Calcium Channel	Calcium	57.08%	19.77%
S2038	Phentolamine Mesylate	Adrenergic Receptor	Adrenergic Receptor	57.08%	19.77%
S4038	Dibucaine HCl	Sodium Channel	Sodium	57.15%	19.70%
S1404	Trilostane	Dehydrogenase	Dehydrogenas	57.21%	19.64%
S2790	Istradefylline	Adenosine Receptor	Adenosine	57.28%	19.57%
S2208	Formestane	Aromatase	Aromatase	57.28%	19.57%
S1941	Enalapril Maleate	RAAS	RAAS	57.41%	19.44%
S2060	Bromhexine HCl	Others	Others	57.41%	19.44%
S1816	Chlorpheniramine Maleate	Histamine Receptor	Histamine Receptor	57.47%	19.38%
S1238	Tamoxifen	Estrogen/progestogen Receptor,Autophagy	Estrogen/progestogen Receptor,Autop	57.54%	19.31%
S1603	Furosemide	Sodium Channel	Sodium	57.54%	19.31%
S2344	Piperine	P450 (e.g. CYP17)	P450 (e.g.	57.60%	19.25%
S2256	4-Methylumbelliferon	Others	Others	57.67%	19.18%
S1960	Pranoprofen	COX	COX	57.67%	19.18%
S4502	Eltrombopag	Thrombin	Thrombin	57.73%	19.12%
S1439	Tranilast	Immunology & Inflammation related	Immunology & Inflammation	57.73%	19.12%
S1811	Amiloride HCl	Sodium Channel,Calcium Channel	Sodium Channel,Calcium Channel	57.86%	18.99%
S1741	Rifabutin	Anti-infection	Anti-infection	57.99%	18.86%

S4046	Estradiol Cypionate	Estrogen/progestogen Receptor	Estrogen/progestogen	58.12%	18.73%
S1655	Ezetimibe	LDL	LDL	58.18%	18.67%
S4797	Nicergoline	Androgen Receptor	Androgen	58.31%	18.54%
S2471	Gallamine	AChR	AChR	58.31%	18.54%
S1716	Glyburide (Glibenclamide)	Potassium Channel	Potassium Channel	58.31%	18.54%
S4727	Cinnarizine	Calcium Channel	Calcium	58.38%	18.47%
S3714	Lifitegrast	Integrin	Integrin	58.44%	18.41%
S2085	Trimebutine	Opioid Receptor	Opioid	58.44%	18.41%
S1748	Nisoldipine	Calcium Channel	Calcium	58.44%	18.41%
S2807	Dabrafenib (GSK2118436)	Raf	Raf	58.51%	18.34%
S1713	Piroxicam	COX	COX	58.51%	18.34%
S1482	Daclatasvir (BMS-790052)	HCV Protease	HCV Protease	58.57%	18.28%
S2293	DL-Carnitine HCl	Others	Others	58.64%	18.21%
S4373	Dicyclomine HCl	Others	Others	58.70%	18.15%
S1807	Aciclovir	Anti-infection	Anti-infection	58.70%	18.15%
S2787	Laquinimod	Immunology & Inflammation related	Immunology & Inflammation	58.77%	18.08%
S1727	Levonorgestrel	Estrogen/progestogen Receptor	Estrogen/progestogen	58.77%	18.08%
S1651	Telbivudine	Reverse Transcriptase	Reverse Transcriptase	58.77%	18.08%
S2349	Rutaecarpine	COX	COX	58.83%	18.02%
S1385	Mosapride Citrate	5-HT Receptor（5-HT4受体激动药，能促进乙酰胆碱的释放）	5-HT Receptor（5-HT4受体激动药，能促进乙酰胆碱的释	58.90%	17.95%
S1606	Clotrimazole	Anti-infection	Anti-infection	58.90%	17.95%
S1777	Ethionamide	Anti-infection	Anti-infection	58.96%	17.89%
S1618	Sulfameter	DHFR	DHFR	59.16%	17.69%
S1633	Zafirlukast	LTR	LTR	59.16%	17.69%
S4091	Ifenprodil Tartrate	NMDAR	NMDAR	59.22%	17.63%
S1657	Enalaprilat	RAAS	RAAS	59.29%	17.56%
S3079	Atovaquone	Anti-infection	Anti-infection	59.29%	17.56%
S2499	Phenoxybenzamine HCl	Adrenergic Receptor	Adrenergic Receptor	59.29%	17.56%
S1336	Fluvoxamine maleate	5-HT Receptor（有效的选择性血清素 (5-HT) 再摄取抑制剂（SSRI））	5-HT Receptor（有效的选择性血清素 (5-HT) 再摄取抑制	59.42%	17.43%
S1829	Pranlukast	Immunology & Inflammation related	Immunology & Inflammation	59.61%	17.24%
S2479	Lincomycin HCl	Anti-infection	Anti-infection	59.61%	17.24%
S2091	Betaxolol	Adrenergic Receptor	Adrenergic Receptor	59.81%	17.04%
S1689	Meprednisone	Glucocorticoid Receptor	Glucocorticoid Receptor	59.87%	16.98%
S4264	Etofibrate	Others	Others	59.93%	16.92%
S1304	Megestrol Acetate	Androgen Receptor,Estrogen/progestogen Receptor	Androgen Receptor,Estrogen/progestogen Receptor	59.93%	16.92%
S1690	Betamethasone Valerate	Glucocorticoid Receptor	Glucocorticoid Receptor	60.00%	16.85%
S2058	Tolnaftate	Anti-infection	Anti-infection	60.06%	16.79%
S2108	Flunixin Meglumin	COX	COX	60.19%	16.66%
S1312	Streptozotocin	DNA alkylator	DNA alkylator	60.26%	16.59%

S3984	Nordihydroguaiaretic acid (NDGA)	Lipoxygenase	Lipoxygenase	60.32%	16.53%
S2452	Amfebutamone (Bupropion) HCl	AChR,Dopamine Receptor	AChR,Dopamine Receptor	60.32%	16.53%
S1261	Celecoxib	COX	COX	60.58%	16.27%
S2029	Uridine	DNA/RNA Synthesis	DNA/RNA	60.58%	16.27%
S2287	Cytisine	AChR	AChR	60.65%	16.20%
S3635	Medroxyprogesterone	Estrogen/progestogen Receptor	Estrogen/progestogen	60.65%	16.20%
S1737	Prednisolone	Immunology & Inflammation related,Glucocorticoid Receptor	Immunology & Inflammation related,Glucocorticoid	60.71%	16.14%
S2101	Gabexate Mesylate	Serine Protease	Serine	60.71%	16.14%
S1271	Acarbose	Others	Others	60.71%	16.14%
S1614	Riluzole	GluR,Sodium Channel	GluR,Sodium Channel	60.78%	16.07%
S3023	Bufexamac	COX	COX	60.78%	16.07%
S1196	Exemestane	Aromatase	Aromatase	60.84%	16.01%
S3052	Rupatadine	Histamine Receptor	Histamine	60.91%	15.94%
S2090	Dexmedetomidine HCl	Adrenergic Receptor	Adrenergic Receptor	60.97%	15.88%
S4230	Oxaprozin	COX	COX	60.97%	15.88%
S1540	Saxagliptin	DPP-4	DPP-4	61.04%	15.81%
S2320	Luteolin	PDE	PDE	61.10%	15.75%
S2042	Cyproterone	Androgen Receptor	Androgen	61.10%	15.75%
S1235	Letrozole	Aromatase	Aromatase	61.17%	15.68%
S1681	Mesalamine	IκB/IKK,Immunology & Inflammation related	Iκ B/IKK,Immunology &	61.17%	15.68%
S3727	Vilanterol Trifenate	Adrenergic Receptor	Adrenergic Receptor	61.23%	15.62%
S1331	Fluconazole	P450 (e.g. CYP17)	P450 (e.g.	61.23%	15.62%
S1324	Doxazosin Mesylate	Adrenergic Receptor	Adrenergic Receptor	61.30%	15.55%
S1705	Progesterone	Estrogen/progestogen Receptor	Estrogen/progestogen	61.30%	15.55%
S1029	Lenalidomide (CC-	TNF-alpha	TNF-alpha	61.36%	15.49%
S1801	Ranitidine	Histamine Receptor	Histamine	61.36%	15.49%
S2059	Terazosin HCl Dihydrate	Adrenergic Receptor	Adrenergic Receptor	61.36%	15.49%
S4588	Docusate Sodium	Opioid Receptor	Opioid	61.43%	15.42%
S1626	Naproxen Sodium	COX	COX	61.49%	15.36%
S4089	Halobetasol Propionate	Phospholipase (e.g. PLA)	Phospholipase (e.g. PLA)	61.56%	15.29%
S1820	Clofibrate	Others	Others	61.56%	15.29%
S1784	Vidarabine	DNA/RNA Synthesis	DNA/RNA	61.62%	15.23%
S2109	Imidapril HCl	RAAS	RAAS	61.81%	15.04%
S2380	Diosmetin	P450 (e.g. CYP17)	P450 (e.g.	61.88%	14.97%
S1381	Meropenem	Anti-infection	Anti-infection	61.88%	14.97%
S2015	Suplatast Tosylate	IL Receptor	IL Receptor	61.88%	14.97%
S2262	Apigenin	P450 (e.g. CYP17)	P450 (e.g.	62.01%	14.84%
S1643	Ursodiol	Others	Others	62.07%	14.78%
S2304	Gramine	Others	Others	62.07%	14.78%
S2461	Domperidone	Dopamine Receptor	Dopamine	62.14%	14.71%
S1247	Leflunomide	Dehydrogenase	Dehydrogenas	62.14%	14.71%
S1538	Telaprevir (VX-950)	HCV Protease	HCV Protease	62.14%	14.71%
S2053	Cytidine	Others	Others	62.14%	14.71%
S4026	Hydroxyzine 2HCl	Histamine Receptor	Histamine	62.33%	14.52%
S2065	Lafutidine	Histamine Receptor	Histamine	62.33%	14.52%

S1653	Tretinoin	Retinoid Receptor	Retinoid	62.40%	14.45%
S1548	Dapagliflozin	SGLT	SGLT	62.40%	14.45%
S2123	Dextrose	0	0	62.46%	14.39%
S1444	Ziprasidone HCl	5-HT Receptor,Dopamine Receptor（5HT2，5HT2A, 5HT2C，）	5-HT Receptor,Dopamine Receptor（5HT2，	62.46%	14.39%
S1921	Phenindione	Vitamin	Vitamin	62.53%	14.32%
S3001	Clevudine	DNA/RNA Synthesis	DNA/RNA	62.59%	14.26%
S4528	Furazolidone	Anti-infection	Anti-infection	62.72%	14.13%
S1639	Amprenavir	HIV Protease	HIV Protease	62.72%	14.13%
S1396	Resveratrol	Autophagy	Autophagy	62.72%	14.13%
S4547	8-Hydroxyquinoline	Anti-infection	Anti-infection	62.79%	14.06%
S1291	Cetirizine DiHCl	Histamine Receptor	Histamine	62.79%	14.06%
S2154	Dabigatran	Thrombin	Thrombin	62.79%	14.06%
S4195	Broxyquinoline	Anti-infection	Anti-infection	62.85%	14.00%
S3036	Pravastatin sodium	HMG-CoA Reductase	HMG-CoA Reductase	62.85%	14.00%
S3733	Boceprevir	HCV Protease	HCV Protease	62.85%	14.00%
S2289	Daidzin	Dehydrogenase	Dehydrogenas	62.85%	14.00%
S2082	Adiphenine HCl	AChR	AChR	62.85%	14.00%
S3120	Doxepin HCl	Histamine Receptor	Histamine	62.92%	13.93%
S2092	Detomidine HCl	Adrenergic Receptor	Adrenergic Receptor	62.98%	13.87%
S1401	Tenofovir	Reverse Transcriptase	Reverse Transcriptase	63.05%	13.80%
S1294	Cilostazol	PDE	PDE	63.18%	13.67%
S2364	Tanshinone I	Phospholipase (e.g. PLA)	Phospholipase (e.g. PLA)	63.24%	13.61%
S1407	Bimatoprost	Immunology & Inflammation related	Immunology & Inflammation	63.37%	13.48%
S1764	Rifampin	DNA/RNA Synthesis	DNA/RNA	63.43%	13.42%
S4685	Efavirenz	Reverse Transcriptase	Reverse Transcriptase	63.63%	13.22%
S2131	Roflumilast	PDE	PDE	63.63%	13.22%
S2907	Pirfenidone	TGF-beta/Smad	TGF-	63.76%	13.09%
S3781	Ginkgolide C	Others	Others	63.95%	12.90%
S2593	Tolvaptan	Vasopressin Receptor	Vasopressin Receptor	64.08%	12.77%
S2083	Procaterol HCl	Adrenergic Receptor	Adrenergic Receptor	64.15%	12.70%
S3160	Ethynodiol diacetate	Estrogen/progestogen Receptor	Estrogen/progestogen	64.28%	12.57%
S3704	4-Methylbenzylidene	Others	Others	64.41%	12.44%
S1884	Sparfloxacin	Anti-infection	Anti-infection	64.41%	12.44%
S4203	Furaltadone HCl	Anti-infection	Anti-infection	64.47%	12.38%
S4281	Tasimelteon	Melatonin Receptor	Melatonin	64.47%	12.38%
S1342	Genistein	EGFR,Topoisomerase	EGFR,Topoisomerase	64.47%	12.38%
S4638	Desogestrel	Estrogen/progestogen Receptor	Estrogen/progestogen	64.54%	12.31%
S3033	Vildagliptin (LAF-	DPP-4	DPP-4	64.54%	12.31%
S2437	Rotundine	Dopamine Receptor	Dopamine	64.60%	12.25%
S4019	Avanafil	PDE	PDE	64.67%	12.18%
S2099	Temocapril HCl	RAAS	RAAS	64.67%	12.18%
S1762	Pyrazinamide	Anti-infection	Anti-infection	64.73%	12.12%
S4561	Danthron	Others	Others	64.80%	12.05%
S2569	Phenylephrine HCl	Adrenergic Receptor	Adrenergic Receptor	64.80%	12.05%

S4110	Estradiol Benzoate	Estrogen/progestogen Receptor	Estrogen/progestogen	64.86%	11.99%
S1379	Isotretinoin	Hydroxylase	Hydroxylase	64.86%	11.99%
S3121	Ornidazole	Anti-infection	Anti-infection	64.86%	11.99%
S2233	Esomeprazole	ATPase	ATPase	64.86%	11.99%
S1888	Deflazacort	Glucocorticoid Receptor	Glucocorticoid Receptor	64.86%	11.99%
S4683	Sildenafil Mesylate	PDE	PDE	64.93%	11.92%
S2250	(-)-Epigallocatechin Gallate	DNA Methyltransferase,HER2,Telomerase,EGFR,Fatty Acid Synthase	DNA Methyltransferase,HER2,Telomerase,EGFR, Fatty Acid	64.93%	11.92%
S1374	Doripenem Hydrate	Anti-infection	Anti-infection	64.93%	11.92%
S2903	Lumiracoxib	COX	COX	64.99%	11.86%
S3675	Umbelliferone	Others	Others	65.25%	11.60%
S1221	Dacarbazine	DNA/RNA Synthesis	DNA/RNA	65.31%	11.54%
S2390	Polydatin	Phospholipase (e.g. PLA)	Phospholipase (e.g. PLA)	65.31%	11.54%
S1014	Bosutinib (SKI-606)	Src	Src	65.38%	11.47%
S1377	Drospirenone	Estrogen/progestogen Receptor	Estrogen/progestogen	65.38%	11.47%
S4292	Diphenidol HCl	AChR	AChR	65.44%	11.41%
S4165	Benzydamine HCl	Immunology & Inflammation related	Immunology & Inflammation	65.57%	11.28%
S2102	Rasagiline	MAO	MAO	65.57%	11.28%
S1197	Finasteride	5-alpha Reductase	5-alpha	65.64%	11.21%
S4584	Butylparaben	Anti-infection	Anti-infection	65.70%	11.15%
S4155	Chlorzoxazone	P450 (e.g. CYP17)	P450 (e.g.	65.70%	11.15%
S3124	Dexamethasone Acetate	Autophagy,IL Receptor	Autophagy,IL Receptor	65.70%	11.15%
S3083	Indacaterol Maleate	Adrenergic Receptor	Adrenergic Receptor	65.90%	10.95%
S2900	Cobicistat (GS-	P450 (e.g. CYP17)	P450 (e.g.	65.90%	10.95%
S1332	Flumazenil	GABA Receptor	GABA	65.90%	10.95%
S1855	Pefloxacin	Anti-infection	Anti-infection	65.90%	10.95%
S1865	Diltiazem HCl	Calcium Channel	Calcium	65.90%	10.95%
S3167	Altrenogest	Estrogen/progestogen Receptor	Estrogen/progestogen	65.96%	10.89%
S1768	Cefditoren Pivoxil	Anti-infection	Anti-infection	65.96%	10.89%
S1354	Lansoprazole	Proton Pump	Proton Pump	65.96%	10.89%
S1806	Acipimox	Others	Others	66.03%	10.82%
S2309	Hesperidin	Others	Others	66.03%	10.82%
S4718	Acetylcholine	AChR	AChR	66.09%	10.76%
S2359	Sinomenine	Others	Others	66.09%	10.76%
S1978	Methscopolamine	AChR	AChR	66.16%	10.69%
S1405	Vecuronium	AChR	AChR	66.22%	10.63%
S2086	Ivabradine HCl	Adrenergic Receptor	Adrenergic Receptor	66.22%	10.63%
S2357	Silibinin	Others	Others	66.22%	10.63%
S3751	Quinidine sulfate	Sodium Channel	Sodium	66.29%	10.56%
S1645	Ketoprofen	COX	COX	66.35%	10.50%
S1445	Zonisamide	Sodium Channel	Sodium	66.35%	10.50%
S2454	Bupivacaine HCl	Sodium Channel	Sodium	66.55%	10.30%
S3732	Avibactam sodium	Anti-infection	Anti-infection	66.61%	10.24%
S1344	Glimepiride	Potassium Channel	Potassium	66.61%	10.24%
S1243	Agomelatine	5-HT Receptor（5-HT2C 受体的竞争性拮抗剂）	5-HT Receptor （5-HT2C 受体的竞争性拮	66.61%	10.24%
S1607	Rizatriptan	5-HT Receptor	5-HT Receptor	66.68%	10.17%

S4249	Flopropione	5-HT Receptor（5-HT1A 受体拮抗）	5-HT Receptor（5-HT1A 受体	66.74%	10.11%
S4362	Glafenine HCl	Others	Others	66.87%	9.98%
S1334	Flupirtine maleate	DNA/RNA Synthesis	DNA/RNA	66.87%	9.98%
S4051	Nabumetone	COX	COX	66.93%	9.92%
S2346	Puerarin	5-HT Receptor（5-HT2C）	5-HT Receptor（5-HT2C）	66.93%	9.92%
S1878	Ganciclovir	Anti-infection	Anti-infection	67.00%	9.85%
S1326	Edaravone	Others	Others	67.06%	9.79%
S4389	Bephenium Hydroxynaphthoate	Others	Others	67.06%	9.79%
S1010	Nintedanib (BIBF 1120)	FGFR,PDGFR,VEGFR	FGFR,PDGFR,VEGFR	67.13%	9.72%
S2054	Orphenadrine	AChR	AChR	67.13%	9.72%
S1756	Enoxacin	Topoisomerase	Topoisomerase	67.13%	9.72%
S1898	Tropisetron HCl	5-HT Receptor（5-HT3受体拮抗剂）	5-HT Receptor（5-HT3受体拮	67.26%	9.59%
S1206	Bisoprolol fumarate	Adrenergic Receptor	Adrenergic Receptor	67.32%	9.53%
S1188	Anastrozole	Aromatase	Aromatase	67.32%	9.53%
S4607	2,2′-Dihydroxy-4-methoxybenzophen	Others	Others	67.39%	9.46%
S2391	Quercetin	Src,Sirtuin,PKC,PI3K	Src,Sirtuin,PKC,PI3K	67.39%	9.46%
S1280	Amisulpride	Dopamine Receptor	Dopamine	67.45%	9.40%
S1845	Cimetidine	Histamine Receptor	Histamine	67.45%	9.40%
S1896	Hydroxyurea	DNA/RNA Synthesis	DNA/RNA	67.45%	9.40%
S1662	Isradipine	Calcium Channel	Calcium	67.45%	9.40%
S4612	Dapson	Anti-infection	Anti-infection	67.52%	9.33%
S1565	VX-809	CFTR	CFTR	67.52%	9.33%
S2124	Xylose	Others	Others	67.52%	9.33%
S1437	Tizanidine HCl	Adrenergic Receptor	Adrenergic Receptor	67.58%	9.27%
S3200	Triflusal	Thrombin	Thrombin	67.65%	9.20%
S1770	Sulfadiazine	Anti-infection	Anti-infection	67.65%	9.20%
S1636	Amphotericin B	Anti-infection	Anti-infection	67.65%	9.20%
S1903	Diclofenac Sodium	COX	COX	67.71%	9.14%
S2443	Tolbutamide	Potassium Channel	Potassium	67.84%	9.01%
S2024	Ketotifen Fumarate	Histamine Receptor	Histamine	67.91%	8.94%
S1866	Diphenhydramine	Histamine Receptor	Histamine	67.91%	8.94%
S4253	Epinastine HCl	Histamine Receptor	Histamine	67.97%	8.88%
S1734	Meloxicam	COX	COX	67.97%	8.88%
S3175	Atomoxetine HCl	5-HT Receptor（选择性去甲肾上腺素再摄取抑制剂 (noradrenaline reuptake)，去甲肾上腺素 (NE)、5-羟色胺 (5-HT) 和多巴胺 (DA)）	5-HT Receptor（选择性去甲肾上腺素再摄取抑制剂 (noradrenaline reuptake)，去甲肾上腺素 (NE)、5-羟色胺	67.97%	8.88%
S4049	Valdecoxib	COX	COX	68.04%	8.81%
S2386	Indirubin	GSK-3	GSK-3	68.04%	8.81%
S1611	Cefoperazone	Anti-infection	Anti-infection	68.10%	8.75%
S3031	Linagliptin	DPP-4	DPP-4	68.17%	8.68%
S2542	Phenformin HCl	AMPK	AMPK	68.17%	8.68%
S1591	Bestatin	Immunology & Inflamm	Immunology & Inflamm	68.17%	8.68%
S1964	Rimantadine	Anti-infection	Anti-infection	68.23%	8.62%
S2373	Yohimbine HCl	Adrenergic Receptor	Adrenergic Receptor	68.30%	8.55%

S2051	Captopril	RAAS	RAAS	68.30%	8.55%
S1933	Triamcinolone	Glucocorticoid Receptor	Glucocorticoid Receptor	68.30%	8.55%
S3654	Tauroursodeoxycholic Acid (TUDCA)	Others	Others	68.30%	8.55%
S2007	Sulindac	COX	COX	68.30%	8.55%
S4021	Tolcapone	Transferase	Transferase	68.36%	8.49%
S1245	Latrepirdine 2HCl	5-HT Receptor,GluR,Histamine Receptor（多靶点拮抗剂，包括histamine receptors、GluR和5-HT receptors）	5-HT Receptor,GluR,Histamine Receptor（多靶点拮抗剂，包括histamine receptors	68.36%	8.49%
S1683	Ipratropium	AChR	AChR	68.36%	8.49%
S4189	Cyclandelate	Others	Others	68.43%	8.42%
S1761	Suprofen	COX	COX	68.43%	8.42%
S1193	Thalidomide	E3 Ligase ,TNF-alpha	E3 Ligase ,TNF-alpha	68.49%	8.36%
S1890	Nizatidine	Histamine Receptor	Histamine	68.49%	8.36%
S2336	Orotic acid (6-Carboxyuracil)	Others	Others	68.49%	8.36%
S4031	Aclidinium Bromide	AChR	AChR	68.55%	8.30%
S1793	Ramipril	RAAS	RAAS	68.55%	8.30%
S4268	Flufenamic acid	Immunology & Inflammation related	Immunology & Inflammation	68.55%	8.30%
S3893	Bornyl acetate	Immunology & Inflammation related	Immunology & Inflammation	68.62%	8.23%
S2052	Oxytetracycline Dihydrate	Anti-infection	Anti-infection	68.62%	8.23%
S2760	Canagliflozin	SGLT	SGLT	68.68%	8.17%
S1849	Daidzein	Others	Others	68.75%	8.10%
S2410	Paeoniflorin	Others	Others	68.75%	8.10%
S1950	Metformin HCl	Autophagy	Autophagy	68.75%	8.10%
S4113	Desvenlafaxine	5-HT Receptor,Adrenergic Receptor（能透过血脑屏障的 5-HT 和 去甲肾上腺素(norepinephrine) 再摄取的抑制剂）	5-HT Receptor,Adrenergic Receptor（能透过血脑屏障的 5-HT 和 去甲肾上腺素	68.81%	8.04%
S1742	Nevirapine	Reverse Transcriptase	Reverse Transcriptase	68.81%	8.04%
S2411	Geniposide	Others	Others	68.81%	8.04%
S1916	Sulfisoxazole	Anti-infection	Anti-infection	68.88%	7.97%
S2383	Gastrodin	Immunology & Inflammation related	Immunology & Inflammation	69.01%	7.84%
S4260	Tamibarotene	Retinoid Receptor	Retinoid	69.07%	7.78%
S1971	Nicorandil	Potassium Channel	Potassium	69.20%	7.65%
S2328	Nalidixic acid	Topoisomerase	Topoisomerase	69.20%	7.65%
S2130	Atropine sulfate monohydrate	AChR	AChR	69.20%	7.65%
S1222	Dexrazoxane HCl (ICRF-187, ADR-	Topoisomerase	Topoisomerase	69.20%	7.65%
S1894	Valsartan	RAAS	RAAS	69.20%	7.65%
S1907	Metronidazole	DNA/RNA Synthesis	DNA/RNA	69.20%	7.65%
S1717	Fomepizole	Dehydrogenase	Dehydrogenas	69.27%	7.58%
S4673	Etonogestrel	Estrogen/progestogen Receptor	Estrogen/progestogen	69.33%	7.52%
S2883	4-Aminohippuric	Others	Others	69.33%	7.52%

S2199	Aliskiren	RAAS	RAAS	69.33%	7.52%
S4309	Bromocriptine	Others	Others	69.46%	7.39%
S2607	Buflomedil HCl	Adrenergic Receptor	Adrenergic Receptor	69.46%	7.39%
S2025	Urapidil HCl	5-HT Receptor（兴奋5-羟色胺-1A受体）	5-HT Receptor（兴奋5-羟色胺-1A受体）	69.46%	7.39%
S4571	Hexylresorcinol	Tyrosinase	Tyrosinase	69.53%	7.32%
S3117	Oxybutynin	AChR	AChR	69.59%	7.26%
S3054	Alverine Citrate	OX Receptor	OX Receptor	69.66%	7.19%
S4034	Diphemanil Methylsulfate	AChR	AChR	69.72%	7.13%
S1709	Estradiol	Estrogen/progestogen Receptor	Estrogen/progestogen	69.72%	7.13%
S2339	Paeonol	MAO	MAO	69.72%	7.13%
S2362	Synephrine	Adrenergic Receptor	Adrenergic Receptor	69.72%	7.13%
S3724	Velpatasvir	HCV Protease	HCV Protease	69.85%	7.00%
S2503	Racecadotril	Opioid Receptor	Opioid	69.85%	7.00%
S4086	Loxapine Succinate	5-HT Receptor,Dopamine Receptor	5-HT Receptor,Dopamine Receptor	69.92%	6.93%
S1701	Desonide	Glucocorticoid Receptor	Glucocorticoid Receptor	69.92%	6.93%
S1696	Hydrocortisone	Glucocorticoid Receptor	Glucocorticoid Receptor	70.05%	6.80%
S2393	Sorbitol	Others	Others	70.24%	6.61%
S1908	Flutamide	Androgen Receptor	Androgen	70.30%	6.55%
S4173	1-Hexadecanol	Others	Others	70.37%	6.48%
S1635	Erythromycin	Anti-infection	Anti-infection	70.37%	6.48%
S2325	Morin Hydrate	Others	Others	70.37%	6.48%
S4559	Cloxiquine	Anti-infection	Anti-infection	70.43%	6.42%
S3854	Tetrahydropalmatine hydrochloride	Others	Others	70.43%	6.42%
S2127	S- (+)-Rolipram	PDE	PDE	70.43%	6.42%
S1250	Enzalutamide (MDV3100)	Androgen Receptor	Androgen Receptor	70.43%	6.42%
S1952	Methoxsalen	P450 (e.g. CYP17)	P450 (e.g.	70.43%	6.42%
S3045	Cinepazide	Calcium Channel	Calcium	70.50%	6.35%
S1164	Lenvatinib (E7080)	VEGFR	VEGFR	70.50%	6.35%
S2608	Fluocinonide	Glucocorticoid Receptor	Glucocorticoid Receptor	70.50%	6.35%
S3647	Mafenide Acetate	Carbonic Anhydrase	Carbonic	70.50%	6.35%
S2078	Famotidine	Histamine Receptor	Histamine	70.50%	6.35%
S2508	Scopolamine HBr	AChR	AChR	70.50%	6.35%
S1212	Bendamustine HCl	DNA/RNA Synthesis	DNA/RNA	70.63%	6.22%
S3739	Calcipotriene	Others	Others	70.69%	6.16%
S1441	Venlafaxine HCl	5-HT Receptor（5-羟色胺-去甲肾上腺素再摄取抑制剂（SNRI））	5-HT Receptor（5-羟色胺-去甲肾上腺素再摄取抑制剂	70.69%	6.16%
S1747	Nimodipine	Autophagy,Calcium Channel	Autophagy,Calcium Channel	70.69%	6.16%
S4114	Triclabendazole	Microtubule Associated	Microtubule Associated	70.76%	6.09%
S4734	Retigabine 2HCl	Potassium Channel	Potassium	70.82%	6.03%
S2119	Probucol	LDL	LDL	70.82%	6.03%
S1899	Nicotinamide (Vitamin B3)	Sirtuin	Sirtuin	70.82%	6.03%

S1702	Didanosine	Reverse Transcriptase	Reverse Transcriptase	70.89%	5.96%
S2258	Esculin	Others	Others	70.89%	5.96%
S4699	Etretinate	Retinoid Receptor	Retinoid	70.95%	5.90%
S2614	Arecoline HBr	AChR	AChR	70.95%	5.90%
S3741	Benznidazole	Anti-infection	Anti-infection	71.02%	5.83%
S4225	Mexiletine HCl	Sodium Channel	Sodium	71.02%	5.83%
S2381	D-Mannitol	Others	Others	71.02%	5.83%
S4377	Imipramine HCl	Others	Others	71.08%	5.77%
S4288	Chloroambucil	DNA/RNA Synthesis	DNA/RNA	71.15%	5.70%
S2097	Ambrisentan	Endothelin Receptor	Endothelin	71.15%	5.70%
S2069	Argatroban	Thrombin	Thrombin	71.15%	5.70%
S3078	Beclomethasone dipropionate	Glucocorticoid Receptor	Glucocorticoid Receptor	71.21%	5.64%
S3919	Hederacoside C	Others	Others	71.21%	5.64%
S1631	Allopurinol Sodium	ROS	ROS	71.21%	5.64%
S1658	Dofetilide	Potassium Channel	Potassium	71.28%	5.57%
S2296	Enoxolone	Dehydrogenase	Dehydrogenas	71.28%	5.57%
S2113	Cisatracurium Besylate	Adrenergic Receptor	Adrenergic Receptor	71.28%	5.57%
S2874	Camostat Mesilate	Sodium Channel	Sodium	71.34%	5.51%
S2351	Salicin	COX	COX	71.41%	5.44%
S4591	Nitroxoline	Topoisomerase	Topoisomerase	71.47%	5.38%
S2384	Hematoxylin	Others	Others	71.47%	5.38%
S2596	Clindamycin palmitate HCl	Others	Others	71.54%	5.31%
S2282	Cinchonidine	Anti-infection	Anti-infection	71.54%	5.31%
S3186	Azatadine	Histamine Receptor	Histamine	71.60%	5.25%
S1576	Sulfasalazine	Immunology & Inflammation related	Immunology & Inflammation	71.60%	5.25%
S1715	Glipizide	Others	Others	71.67%	5.18%
S2438	Synephrine HCl	Adrenergic Receptor	Adrenergic Receptor	71.73%	5.12%
S4250	Sulfamethoxypyrid	Anti-infection	Anti-infection	71.80%	5.05%
S2177	Dichlorphenamide (Diclofenamide)	Others	Others	71.80%	5.05%
S3998	(+)-α-Lipoic acid	Others	Others	71.86%	4.99%
S4102	Eprosartan	RAAS	RAAS	71.93%	4.92%
S3074	Chlorthalidone	Others	Others	71.93%	4.92%
S1619	Prilocaine	Sodium Channel	Sodium	71.93%	4.92%
S1578	Candesartan	RAAS	RAAS	71.93%	4.92%
S2506	Roxithromycin	Anti-infection	Anti-infection	71.99%	4.86%
S4839	Mosapride	5-HT Receptor（5-HT4 receptor激动剂）	5-HT Receptor （5-HT4 receptor激动	71.99%	4.86%
S1340	Gatifloxacin	Topoisomerase	Topoisomerase	72.05%	4.80%
S4200	Tolperisone HCl	Sodium Channel	Sodium	72.12%	4.73%
S4202	Verapamil HCl	Calcium Channel	Calcium	72.12%	4.73%
S2016	Mirtazapine	5-HT Receptor（5-HT2、5-HT3，组胺 H1 受体 (histamine H1 receptor) 和 α2-肾上腺素受体 (α2-adrenoceptor) 拮抗剂）	5-HT Receptor （5-HT2、5-HT3，组胺 H1 受体 (histamine H1 receptor) 和 α2-肾上腺素受体 (α2-	72.12%	4.73%
S1962	Sulphadimethoxine	Anti-infection	Anti-infection	72.12%	4.73%
S1679	Flurbiprofen	Immunology & Inflammation related	Immunology & Inflammation	72.18%	4.67%
S3824	Quercitrin	Immunology & Inflammation related	Immunology & Inflammation	72.25%	4.60%

S2611	Ethisterone	Estrogen/progestogen	Estrogen/progestogen	72.25%	4.60%
S2579	Zidovudine	Reverse Transcriptase	Reverse Transcriptase	72.31%	4.54%
S4071	Griseofulvin	Microtubule Associated	Microtubule Associated	72.31%	4.54%
S4169	Teriflunomide	Dehydrogenase,Immunology & Inflammation related	Dehydrogenase,Immunology & Inflammation	72.51%	4.34%
S1732	Mitotane	Others	Others	72.51%	4.34%
S1677	Chloramphenicol	Anti-infection	Anti-infection	72.51%	4.34%
S1204	Melatonin	MT Receptor	MT Receptor	72.51%	4.34%
S2302	Glycyrrhizin (Glycyrrhizic Acid)	Dehydrogenase,MAO,High-mobility Group	Dehydrogenase,MAO,High-mobility Group	72.51%	4.34%
S2467	Famciclovir	Anti-infection	Anti-infection	72.64%	4.21%
S2011	Pramipexole 2HCl Monohydrate	Dopamine Receptor	Dopamine Receptor	72.64%	4.21%
S2055	Gimeracil	Dehydrogenase	Dehydrogenas	72.64%	4.21%
S2266	Asiatic Acid	p38 MAPK	p38 MAPK	72.64%	4.21%
S4098	Halcinonide	Immunology & Inflammation related	Immunology & Inflammation	72.70%	4.15%
S1897	Potassium Iodide	Others	Others	72.70%	4.15%
S4106	Closantel	Anti-infection	Anti-infection	72.77%	4.08%
S2388	L-carnitine	Others	Others	72.77%	4.08%
S3043	Rofecoxib	COX	COX	72.83%	4.02%
S3847	Panaxatriol	Others	Others	72.83%	4.02%
S1904	Avobenzone	Others	Others	72.83%	4.02%
S3759	Norcantharidin	EGFR,c-Met	EGFR,c-Met	72.90%	3.95%
S1287	Bumetanide	Others	Others	72.90%	3.95%
S4147	Azithromycin	Anti-infection	Anti-infection	72.90%	3.95%
S3892	Isopsoralen	Immunology & Inflammation related	Immunology & Inflammation	72.90%	3.95%
S1915	Sulfamethoxazole	Anti-infection	Anti-infection	72.90%	3.95%
S4507	17-Hydroxyprogestero	Estrogen/progestogen Receptor	Estrogen/progestogen	72.96%	3.89%
S4187	Salicylanilide	Reverse Transcriptase,Integrase,Anti-infection	Reverse Transcriptase,Integrase,Anti-	72.96%	3.89%
S1517	Natamycin	Anti-infection	Anti-infection	72.96%	3.89%
S4583	Butamben	Others	Others	73.09%	3.76%
S1703	Divalproex Sodium	HDAC	HDAC	73.09%	3.76%
S3017	Aspirin	COX	COX	73.09%	3.76%
S2036	Aspartame	Others	Others	73.09%	3.76%
S2533	Ritodrine HCl	Adrenergic Receptor	Adrenergic Receptor	73.16%	3.69%
S1675	Lubiprostone	Chloride	Chloride	73.16%	3.69%
S2446	Levosimendan	Calcium Channel	Calcium	73.29%	3.56%
S3677	Cinnamic acid	Others	Others	73.35%	3.50%
S1891	Carbidopa	Decarboxylase	Decarboxylase	73.42%	3.43%
S3731	Tipiracil	Phosphorylase	Phosphorylase	73.48%	3.37%
S2451	Amantadine HCl	Dopamine Receptor	Dopamine	73.48%	3.37%
S1881	Protionamide	Anti-infection	Anti-infection	73.48%	3.37%
S4604	Levofloxacin	Topoisomerase	Topoisomerase	73.55%	3.30%
S4590	Dithranol	Others	Others	73.55%	3.30%
S1706	Lamivudine	Reverse Transcriptase	Reverse Transcriptase	73.55%	3.30%
S1691	Praziquantel	Anti-infection	Anti-infection	73.55%	3.30%
S4672	Dimenhydrinate	Others	Others	73.61%	3.24%

S2314	Kaempferol	Fatty Acid Synthase,Estrogen/progestogen Receptor	Fatty Acid Synthase,Estrogen/progestogen Receptor	73.61%	3.24%
S4159	Bezafibrate	PPAR	PPAR	73.68%	3.17%
S3641	Osalmid	Others	Others	73.68%	3.17%
S3027	Fenoprofen calcium hydrate	Immunology & Inflammation related	Immunology & Inflammation	73.74%	3.11%
S4077	Mequinol	Others	Others	73.74%	3.11%
S3105	Nadifloxacin	Anti-infection	Anti-infection	73.74%	3.11%
S2389	Naringin Dihydrochalcone	P450 (e.g. CYP17)	P450 (e.g. CYP17)	73.74%	3.11%
S2331	Neohesperidin Dihydrochalcone	Others	Others	73.74%	3.11%
S1858	Dienestrol	Others	Others	73.87%	2.98%
S2103	Naltrexone HCl	Opioid Receptor	Opioid	73.87%	2.98%
S1408	Linezolid	Anti-infection	Anti-infection	73.87%	2.98%
S2299	Formononetin	Others	Others	73.87%	2.98%
S2492	Novobiocin Sodium	Topoisomerase	Topoisomerase	73.87%	2.98%
S1442	Voriconazole	P450 (e.g. CYP17)	P450 (e.g.	73.93%	2.92%
S1573	Fasudil (HA-1077)	Autophagy,ROCK	Autophagy,RO	73.93%	2.92%
S4065	Guanabenz Acetate	Adrenergic Receptor	Adrenergic Receptor	74.00%	2.85%
S2080	Clevidipine	Calcium Channel	Calcium	74.00%	2.85%
S3776	Sophoricoside	Immunology & Inflammation related	Immunology & Inflammation	74.06%	2.79%
S1665	Estrone	Estrogen/progestogen Receptor	Estrogen/progestogen	74.06%	2.79%
S2322	(+)-Matrine	Opioid Receptor	Opioid	74.06%	2.79%
S3133	Sulfamethazine	Anti-infection	Anti-infection	74.13%	2.72%
S2543	Ceftiofur HCl	Anti-infection	Anti-infection	74.13%	2.72%
S1823	Fenoprofen Calcium	Immunology & Inflammation related	Immunology & Inflammation	74.13%	2.72%
S4178	Climbazole	Anti-infection	Anti-infection	74.19%	2.66%
S2316	Kinetin	Others	Others	74.19%	2.66%
S3648	Amlexanox	Immunology & Inflammation related	Immunology & Inflammation	74.26%	2.59%
S2046	Pioglitazone HCl	P450 (e.g. CYP17)	P450 (e.g.	74.26%	2.59%
S2072	Seratrodast(AA-2414, ABT-001)	Others	Others	74.26%	2.59%
S2525	Phenytoin	Sodium Channel	Sodium	74.32%	2.53%
S4838	Acotiamide hydrochloride	AChR	AChR	74.32%	2.53%
S1438	Topiramate	Carbonic Anhydrase	Carbonic	74.32%	2.53%
S2032	Rebamipide	Others	Others	74.32%	2.53%
S1940	Levofloxacin	Topoisomerase	Topoisomerase	74.39%	2.46%
S2329	Naringin	P450 (e.g. CYP17)	P450 (e.g.	74.52%	2.33%
S4706	Eugenol	Immunology & Inflammation related	Immunology & Inflammation	74.58%	2.27%
S4711	Esculetin	Lipoxygenase	Lipoxygenase	74.58%	2.27%
S4210	Benzocaine	Sodium Channel	Sodium	74.65%	2.20%
S4096	Droperidol	Dopamine Receptor	Dopamine	74.65%	2.20%
S1629	Orlistat	Lipase,Fatty Acid Synthase	Lipase,Fatty Acid Synthase	74.65%	2.20%
S1282	Artemisinin	Anti-infection	Anti-infection	74.65%	2.20%
S2105	Pantoprazole	Proton Pump	Proton Pump	74.71%	2.14%
S2396	Salidroside	Others	Others	74.78%	2.07%
S1827	Betaxolol HCl	Adrenergic Receptor	Adrenergic Receptor	74.78%	2.07%
S2338	Oxymatrine	Others	Others	74.84%	2.01%

S2439	Guanosine	Others	Others	74.91%	1.94%
S2347	Quercetin	Others	Others	74.91%	1.94%
S1730	Indapamide	Others	Others	74.91%	1.94%
S3137	Sodium salicylate	NF-κB	NF-κB	75.04%	1.81%
S1605	Cefdinir	Anti-infection	Anti-infection	75.10%	1.75%
S3181	Flumequine	Topoisomerase	Topoisomerase	75.10%	1.75%
S4160	Penicillin G Sodium	Anti-infection	Anti-infection	75.10%	1.75%
S4057	Methyclothiazide	Others	Others	75.17%	1.68%
S3645	Kitasamycin	Anti-infection	Anti-infection	75.17%	1.68%
S1628	Triamcinolone Acetonide	Glucocorticoid Receptor	Glucocorticoid Receptor	75.17%	1.68%
S3185	Adrenalone HCl	Adrenergic Receptor	Adrenergic Receptor	75.23%	1.62%
S4176	Trometamol	COX	COX	75.30%	1.55%
S2519	Naphazoline HCl	Adrenergic Receptor	Adrenergic Receptor	75.30%	1.55%
S1389	Omeprazole	Proton Pump,Autophagy	Proton Pump,Autopha	75.36%	1.49%
S4317	Clorgyline HCl	Others	Others	75.43%	1.42%
S3138	Methylthiouracil	Others	Others	75.43%	1.42%
S3701	Benactyzine hydrochloride	AChR	AChR	75.43%	1.42%
S1840	Lomustine	DNA/RNA Synthesis	DNA/RNA	75.43%	1.42%
S4258	Luliconazole	Anti-infection	Anti-infection	75.49%	1.36%
S3673	Sulfaphenazole	P450 (e.g. CYP17)	P450 (e.g.	75.49%	1.36%
S1790	Rifaximin	DNA/RNA Synthesis	DNA/RNA	75.49%	1.36%
S3132	Sulfamerazine	Anti-infection	Anti-infection	75.49%	1.36%
S4208	Chromocarb	Others	Others	75.55%	1.30%
S2369	Troxerutin	Others	Others	75.55%	1.30%
S2401	Sodium Danshensu	P450 (e.g. CYP17)	P450 (e.g.	75.55%	1.30%
S2337	Osthole	Others	Others	75.62%	1.23%
S2378	Butylscopolamine Bromide	Others	Others	75.62%	1.23%
S4039	Methazolamide	Carbonic Anhydrase	Carbonic	75.68%	1.17%
S3179	Carbenicillin	Anti-infection	Anti-infection	75.68%	1.17%
S4105	Closantel Sodium	Anti-infection	Anti-infection	75.68%	1.17%
S1957	Sulfamethizole	Anti-infection	Anti-infection	75.68%	1.17%
S3212	Moclobemide (Ro 111163)	MAO	MAO	75.75%	1.10%
S2020	Formoterol Hemifumarate	Adrenergic Receptor	Adrenergic Receptor	75.75%	1.10%
S2537	Secnidazole	Anti-infection	Anti-infection	75.75%	1.10%
S4256	Buspirone HCl	5-HT Receptor（5-HT1A receptor激动剂）	5-HT Receptor（5-HT1A receptor激动	75.81%	1.04%
S1876	Valaciclovir HCl	Anti-infection	Anti-infection	75.81%	1.04%
S4535	Methylene Blue	Others	Others	75.88%	0.97%
S3842	Isoquercitrin	Wnt/beta-catenin	Wnt/beta-	75.88%	0.97%
S3063	Diclofenac	COX	COX	75.88%	0.97%
S1937	Isoniazid	Fatty Acid Synthase	Fatty Acid	75.94%	0.91%
S1856	Metoprolol Tartrate	Adrenergic Receptor	Adrenergic Receptor	75.94%	0.91%
S4709	Latanoprost	Immunology & Inflammation related,RAR/RXR,GPR	Immunology & Inflammation related,RAR/RXR,GPR	76.01%	0.84%
S3924	Ginsenoside Rb1	Others	Others	76.01%	0.84%
S4263	Efaproxiral Sodium	Others	Others	76.01%	0.84%
S3730	Metaxalone	Others	Others	76.01%	0.84%
S3711	Carbasalate	Others	Others	76.07%	0.78%

S4334	Mesoridazine	Others	Others	76.14%	0.71%
S3140	Milnacipran HCl	5-HT Receptor（5-羟色胺-去甲肾上腺素重吸收抑制剂（SNRI）	5-HT Receptor（5-羟色胺-去甲肾上腺素重吸收抑制剂	76.14%	0.71%
S4402	Oxeladin Citrate	Others	Others	76.20%	0.65%
S2317	L-(+)-Rhamnose Monohydrate	Others	Others	76.27%	0.58%
S2868	Alogliptin（SYK-322）benzoate	DPP-4	DPP-4	76.33%	0.52%
S1654	Phenylbutazone	Immunology & Inflammation related	Immunology & Inflammation	76.33%	0.52%
S1789	Tetrabenazine (Xenazine)	Dopamine Receptor	Dopamine Receptor	76.33%	0.52%
S1988	Propylthiouracil	Others	Others	76.33%	0.52%
S1512	Tadalafil	PDE	PDE	76.33%	0.52%
S4216	Valnemulin HCl	Anti-infection	Anti-infection	76.33%	0.52%
S2875	Prucalopride	5-HT Receptor（5-HT 4 受体激动剂）	5-HT Receptor（5-HT 4 受体	76.46%	0.39%
S3887	L-Rhamnose monohydrate	Others	Others	76.46%	0.39%
S1415	Clopidogrel	P2 Receptor	P2 Receptor	76.46%	0.39%
S3077	Tazobactam	Anti-infection	Anti-infection	76.46%	0.39%
S4744	Anethole trithione	Others	Others	76.53%	0.32%
S4577	Terpin hydrate	Others	Others	76.59%	0.26%
S2594	Pramiracetam	Others	Others	76.59%	0.26%
S1255	Nepafenac	COX	COX	76.66%	0.19%
S3846	Eupatilin	Others	Others	76.66%	0.19%
S3070	Piracetam	GluR	GluR	76.72%	0.13%
S2308	Hesperetin	TGF-beta/Smad,Histamine Receptor	TGF-beta/Smad,Histamine	76.72%	0.13%
S2021	Chlormezanone	Others	Others	76.72%	0.13%
S3130	Biotin (Vitamin B7)	Vitamin	Vitamin	76.79%	0.06%
S3622	Diammonium Glycyrrhizinate	Immunology & Inflammation related	Immunology & Inflammation	76.85%	0.00%
S3995	Guaiazulene	Others	Others	76.85%	0.00%
S1409	Alfuzosin HCl	Adrenergic Receptor	Adrenergic Receptor	76.85%	0.00%
S3735	Umeclidinium	AChR	AChR	76.92%	-0.07%
S2547	Tiotropium Bromide hydrate	AChR	AChR	76.92%	-0.07%
S1693	Carbamazepine	Sodium Channel,Autophagy	Sodium Channel,Autop	76.92%	-0.07%
S1622	Prednisone	Glucocorticoid Receptor,Immunology & Inflammation related	Glucocorticoid Receptor,Immunology & Inflammation	76.92%	-0.07%
S2453	Benserazide HCl	Decarboxylase	Decarboxylase	77.05%	-0.20%
S1739	Thiabendazole	P450 (e.g. CYP17)	P450 (e.g.	77.05%	-0.20%
S4143	Pentoxyverine	AChR	AChR	77.11%	-0.26%
S2263	Arbutin	Tyrosinase	Tyrosinase	77.11%	-0.26%
S3935	Nonivamide	TRP Channel	TRP Channel	77.18%	-0.33%
S3945	L-Cycloserine	Transferase	Transferase	77.18%	-0.33%
S1500	Betamethasone	Glucocorticoid Receptor	Glucocorticoid Receptor	77.18%	-0.33%
S3797	Helicide	Others	Others	77.24%	-0.39%
S2830	Clindamycin	Others	Others	77.24%	-0.39%
S1623	Acetylcysteine	TNF-alpha,ROS	TNF-	77.24%	-0.39%
S2270	Bergenin	Others	Others	77.24%	-0.39%

S4188	Sasapyrine	Immunology & Inflammation related	Immunology & Inflammation	77.30%	-0.45%
S3672	Cefonicid sodium	Anti-infection	Anti-infection	77.30%	-0.45%
S3690	Pargyline	MAO	MAO	77.43%	-0.58%
S4284	Chloroprocaine HCl	Sodium Channel	Sodium	77.50%	-0.65%
S3021	Rimonabant	Cannabinoid Receptor	Cannabinoid Receptor	77.50%	-0.65%
S1725	Terbinafine	Anti-infection	Anti-infection	77.50%	-0.65%
S4100	Esmolol HCl	Adrenergic Receptor	Adrenergic Receptor	77.63%	-0.78%
S4217	Liothyronine	Others	Others	77.63%	-0.78%
S1359	Losartan Potassium (DuP	RAAS	RAAS	77.63%	-0.78%
S4578	Tyloxapol	Others	Others	77.63%	-0.78%
S1608	Pyridostigmine	AChR	AChR	77.63%	-0.78%
S1251	Dienogest	Estrogen/progestogen Receptor	Estrogen/progestogen	77.63%	-0.78%
S3151	Gliquidone	Potassium Channel	Potassium	77.63%	-0.78%
S4565	Diiodohydroxyquino	Anti-infection	Anti-infection	77.69%	-0.84%
S3835	Loganin	BACE,AChR	BACE,AChR	77.69%	-0.84%
S3965	Vanillyl Butyl Ether	Others	Others	77.76%	-0.91%
S2375	Aloin	Tyrosinase	Tyrosinase	77.76%	-0.91%
S3766	Tanshinone IIA sulfonate (sodium)	CaMK	CaMK	77.82%	-0.97%
S2057	Cyclophosphamide Monohydrate	DNA alkylator	DNA alkylator	77.82%	-0.97%
S3659	Fludrocortisone	Others	Others	77.82%	-0.97%
S1704	Emtricitabine	Reverse Transcriptase	Reverse Transcriptase	77.82%	-0.97%
S2003	Maraviroc	CCR	CCR	77.82%	-0.97%
S1828	Proparacaine HCl	Sodium Channel	Sodium	77.82%	-0.97%
S2840	Apalutamide?(ARN	Androgen Receptor	Androgen	77.89%	-1.04%
S2509	Sotalol HCl	Adrenergic Receptor	Adrenergic Receptor	77.95%	-1.10%
S4064	Escitalopram Oxalate	5-HT Receptor（5-羟色胺(5-HT)重吸收抑制剂(SSRI)）	5-HT Receptor（5-羟色胺(5-HT)重吸收抑制	77.95%	-1.10%
S1647	Adenosine	Adenosine Receptor	Adenosine	77.95%	-1.10%
S2458	Clonidine HCl	Adrenergic Receptor,Autophagy	Adrenergic Receptor,Autop	78.02%	-1.17%
S2045	Doxifluridine	Phosphorylase	Phosphorylase	78.02%	-1.17%
S1300	Tegafur (FT-207, NSC 148958)	DNA/RNA Synthesis	DNA/RNA Synthesis	78.08%	-1.23%
S4040	Norethindrone	Others	Others	78.15%	-1.30%
S2576	Xylometazoline HCl	Adrenergic Receptor	Adrenergic Receptor	78.15%	-1.30%
S1252	Entecavir Hydrate	Reverse Transcriptase	Reverse Transcriptase	78.28%	-1.43%
S4037	Doxapram HCl	Potassium Channel	Potassium	78.34%	-1.49%
S3202	Catharanthine	AChR	AChR	78.34%	-1.49%
S2524	Phenytoin Sodium	Sodium Channel	Sodium	78.34%	-1.49%
S1729	Gemfibrozil	PPAR	PPAR	78.34%	-1.49%
S4239	Bergapten	DNA/RNA Synthesis	DNA/RNA	78.41%	-1.56%
S1213	Nelarabine	DNA/RNA Synthesis	DNA/RNA	78.41%	-1.56%
S1791	Bacitracin Zinc	Anti-infection	Anti-infection	78.41%	-1.56%
S2493	Olanzapine	5-HT Receptor,Dopamine Receptor（5-HT2A、HT2C 、HT3 receptor拮抗剂）	5-HT Receptor,Dopamine Receptor（5-HT2A、HT2C 、HT3	78.47%	-1.62%

S1256	Rufinamide	Sodium Channel	Sodium	78.47%	-1.62%
S3809	Imperatorin	Calcium Channel	Calcium	78.47%	-1.62%
S4082	Spiramycin	Anti-infection	Anti-infection	78.54%	-1.69%
S3640	Methoxyphenamine Hydrochloride	Adrenergic Receptor	Adrenergic Receptor	78.54%	-1.69%
S1928	Alibendol	Others	Others	78.54%	-1.69%
S2366	Taxifolin (Dihydroquercetin)	VEGFR	VEGFR	78.60%	-1.75%
S2529	Dopamine HCl	Dopamine Receptor	Dopamine	78.67%	-1.82%
S2581	Quinapril HCl	RAAS	RAAS	78.67%	-1.82%
S4622	3,4-	Others	Others	78.73%	-1.88%
S4845	Rabeprazole	Proton Pump	Proton Pump	78.73%	-1.88%
S4737	Psoralen	DNA/RNA Synthesis	DNA/RNA	78.73%	-1.88%
S2510	Spectinomycin	Anti-infection	Anti-infection	78.73%	-1.88%
S2564	Cloxacillin Sodium	Anti-infection	Anti-infection	78.80%	-1.95%
S3926	Forsythin	Others	Others	78.80%	-1.95%
S3018	Niflumic acid	COX,GABA Receptor	COX,GABA Receptor	78.80%	-1.95%
S1990	Capsaicin(Vanilloid	Others	Others	78.80%	-1.95%
S1744	Nicotinic Acid	Vitamin	Vitamin	78.93%	-2.08%
S4081	Sulfacetamide Sodium	Anti-infection,Autophagy	Anti-infection,Autop	78.99%	-2.14%
S4139	Cyclizine 2HCl	Histamine Receptor	Histamine	78.99%	-2.14%
S3967	Flavone	Others	Others	78.99%	-2.14%
S1258	Prasugrel	P2 Receptor	P2 Receptor	78.99%	-2.14%
S1373	Daptomycin	Anti-infection,DNA/RNA Synthesis	Anti-infection,DNA/RNA Synthesis	78.99%	-2.14%
S2376	Ammonium Glycyrrhizinate	Dehydrogenase	Dehydrogenase	78.99%	-2.14%
S3173	Antipyrine	COX	COX	79.05%	-2.20%
S3668	Thymopentin	Immunology & Inflammation related	Immunology & Inflammation	79.05%	-2.20%
S2096	Almotriptan Malate	5-HT Receptor（5-HT2A）	5-HT Receptor（5-HT2A）	79.12%	-2.27%
S2387	Lappaconite HBr	Immunology & Inflammation related	Immunology & Inflammation	79.12%	-2.27%
S4111	Dicloxacillin	Anti-infection	Anti-infection	79.18%	-2.33%
S3811	Ginsenoside Re	Others	Others	79.18%	-2.33%
S2071	Prulifloxacin (NM441, AF 3013)	Others	Others	79.18%	-2.33%
S1553	ABT-492(Delafloxacin,	Others	Others	79.25%	-2.40%
S1183	Danoprevir (ITMN-	HCV Protease	HCV Protease	79.25%	-2.40%
S3154	Butenafine HCl	Anti-infection	Anti-infection	79.31%	-2.46%
S4104	Diminazene	Others	Others	79.38%	-2.53%
S3779	cis-Anethole	Others	Others	79.38%	-2.53%
S3705	Chlorobutanol	Anti-infection	Anti-infection	79.38%	-2.53%
S3170	Ampicillin sodium	Anti-infection	Anti-infection	79.44%	-2.59%
S4061	Levobupivacaine	Sodium Channel	Sodium	79.51%	-2.66%
S4118	Histamine 2HCl	Histamine Receptor	Histamine	79.51%	-2.66%
S3889	Arteether	Others	Others	79.51%	-2.66%
S4837	Ibudilast	PDE	PDE	79.57%	-2.72%
S4214	Sucralose	Others	Others	79.64%	-2.79%
S1723	Indomethacin	COX	COX	79.64%	-2.79%
S2599	L-Thyroxine	Others	Others	79.64%	-2.79%
S3703	Phenethyl alcohol	Others	Others	79.70%	-2.85%
S3625	Tyramine	TAAR	TAAR	79.70%	-2.85%
S4543	Trimetazidine dihydrochloride	Others	Others	79.77%	-2.92%

S3655	Cefepime Dihydrochloride Monohydrate	Anti-infection	Anti-infection	79.77%	-2.92%
S3669	Carmustine	DNA/RNA Synthesis	DNA/RNA	79.83%	-2.98%
S2504	Ribavirin	Anti-infection	Anti-infection	79.90%	-3.05%
S1666	Flucytosine	Anti-infection	Anti-infection	79.90%	-3.05%
S4018	Sennoside B	PDGFR	PDGFR	79.96%	-3.11%
S1692	Busulfan	DNA alkylator	DNA alkylator	79.96%	-3.11%
S3062	Diclofenac Potassium	Immunology & Inflammation related	Immunology & Inflammation	79.96%	-3.11%
S2350	Rutin	Immunology & Inflammation related	Immunology & Inflammation	79.96%	-3.11%
S3763	Cinnamaldehyde	TRPV	TRPV	80.03%	-3.18%
S2280	Chlorogenic Acid	Others	Others	80.03%	-3.18%
S1397	Rocuronium	AChR	AChR	80.09%	-3.24%
S3986	L(+)-Arabinose	Others	Others	80.16%	-3.31%
S3774	Dehydroandrographolide Succinate	Immunology & Inflammation related	Immunology & Inflammation	80.16%	-3.31%
S2665	Ciprofibrate	PPAR	PPAR	80.16%	-3.31%
S2610	Lonidamine	Others	Others	80.16%	-3.31%
S1736	Methocarbamol	Carbonic Anhydrase	Carbonic	80.22%	-3.37%
S3983	Caryophyllene	Others	Others	80.35%	-3.50%
S3987	L-Tryptophan	Others	Others	80.35%	-3.50%
S2583	Thiamphenicol	Anti-infection	Anti-infection	80.42%	-3.57%
S2577	Phenacetin	COX	COX	80.48%	-3.63%
S3116	Sulfathiazole	Anti-infection	Anti-infection	80.48%	-3.63%
S4083	Vitamin A Acetate	Vitamin	Vitamin	80.55%	-3.70%
S4161	Benzoic Acid	Others	Others	80.55%	-3.70%
S2466	Estriol	Estrogen/progestogen Receptor	Estrogen/progestogen	80.55%	-3.70%
S4665	Rebeprazole	Proton Pump	Proton Pump	80.61%	-3.76%
S1356	Levetiracetam	Calcium Channel	Calcium	80.61%	-3.76%
S1667	Trichlormethiazide	Others	Others	80.74%	-3.89%
S2512	Tenoxicam	Immunology & Inflammation related	Immunology & Inflammation	80.74%	-3.89%
S3907	Bulleyaconi cine A	Sodium Channel	Sodium	80.74%	-3.89%
S2570	Prednisolone Acetate	Glucocorticoid Receptor	Glucocorticoid Receptor	80.74%	-3.89%
S2264	Artemether	Anti-infection	Anti-infection	80.74%	-3.89%
S4060	Erythromycin Ethylsuccinate	Anti-infection	Anti-infection	80.80%	-3.95%
S3843	Madecassoside	Others	Others	80.80%	-3.95%
S4243	Deoxycorticosterone acetate	Adrenergic Receptor	Adrenergic Receptor	80.87%	-4.02%
S4624	5,5-Dimethyloxazolidin	Others	Others	80.93%	-4.08%
S4231	Pilocarpine HCl	AChR	AChR	80.93%	-4.08%
S1934	Nystatin	Anti-infection	Anti-infection	80.93%	-4.08%
S2269	Baicalin	GABA Receptor	GABA	80.93%	-4.08%
S4351	Ractopamine HCl	Others	Others	81.00%	-4.15%
S1391	Oxcarbazepine	Sodium Channel	Sodium	81.00%	-4.15%
S3104	Moguisteine	Others	Others	81.06%	-4.21%
S2501	Pyrantel Pamoate	Anti-infection	Anti-infection	81.06%	-4.21%
S3802	Trigonelline Hydrochloride	Others	Others	81.13%	-4.28%
S1641	Chlorothiazide	Others	Others	81.13%	-4.28%
S4544	Urethane	Others	Others	81.26%	-4.41%
S4248	Bromfenac Sodium	COX	COX	81.32%	-4.47%
S3129	Trimethoprim	Anti-infection	Anti-infection	81.32%	-4.47%

S3694	Glucosamine hydrochloride	Others	Others	81.32%	-4.47%
S3666	Ilaprazole	Proton Pump	Proton Pump	81.39%	-4.54%
S4190	Cinchophen	Others	Others	81.39%	-4.54%
S3785	Notoginsenoside	Others	Others	81.45%	-4.60%
S1913	Tropicamide	AChR	AChR	81.45%	-4.60%
S2268	Baicalein	P450 (e.g. CYP17)	P450 (e.g.	81.45%	-4.60%
S3180	Eletriptan HBr	5-HT Receptor（5-HT1B 和 5-HT1D 受体激动剂）	5-HT Receptor（5-HT1B 和 5-HT1D 受体激动	81.52%	-4.67%
S4048	Carbimazole	Others	Others	81.52%	-4.67%
S4167	Cyromazine	Anti-infection	Anti-infection	81.52%	-4.67%
S2884	Acesulfame	Others	Others	81.52%	-4.67%
S2574	Tetracycline HCl	Anti-infection	Anti-infection	81.52%	-4.67%
S4812	Ceftizoxime	Anti-infection	Anti-infection	81.58%	-4.73%
S3726	Selexipag	Immunology & Inflammation related	Immunology & Inflammation	81.58%	-4.73%
S4045	Pheniramine	Histamine Receptor	Histamine	81.58%	-4.73%
S1400	Tenofovir Disoproxil	Reverse Transcriptase	Reverse Transcriptase	81.58%	-4.73%
S4166	Chlorpropamide	Others	Others	81.65%	-4.80%
S1843	Chenodeoxycholic	FXR	FXR	81.65%	-4.80%
S4566	DL-Panthenol	Others	Others	81.65%	-4.80%
S2902	S-Ruxolitinib (INCB018424)	JAK	JAK	81.65%	-4.80%
S3681	Vitamin E Acetate	Vitamin	Vitamin	81.65%	-4.80%
S4831	Piperonyl butoxide	Others	Others	81.71%	-4.86%
S2555	Clarithromycin	P450 (e.g. CYP17)	P450 (e.g.	81.71%	-4.86%
S3769	Palmatine	AChR	AChR	81.71%	-4.86%
S4536	Nitrofurantoin	Anti-infection	Anti-infection	81.78%	-4.93%
S4184	Penciclovir	Anti-infection	Anti-infection	81.78%	-4.93%
S2521	Epinephrine bitartrate	Adrenergic Receptor	Adrenergic Receptor	81.84%	-4.99%
S2079	Moexipril HCl	RAAS	RAAS	81.91%	-5.06%
S1969	Nefiracetam	GABA Receptor	GABA	81.91%	-5.06%
S4204	Isosorbide	Others	Others	81.97%	-5.12%
S2300	Ferulic Acid	Others	Others	81.97%	-5.12%
S4635	Cyproheptadine hydrochloride	5-HT Receptor（5-HT2A 受体拮抗剂）	5-HT Receptor（5-HT2A 受体	82.04%	-5.19%
S3708	Sulfachloropyridazi	Anti-infection	Anti-infection	82.04%	-5.19%
S3791	Succinic acid	Others	Others	82.04%	-5.19%
S2374	5-hydroxytryptophan	Others	Others	82.04%	-5.19%
S3155	Mepivacaine HCl	Sodium Channel	Sodium	82.04%	-5.19%
S4349	Procyclidine HCl	Others	Others	82.10%	-5.25%
S2005	Raltegravir (MK-	Integrase	Integrase	82.10%	-5.25%
S4191	Betamipron	Anti-infection	Anti-infection	82.17%	-5.32%
S4095	Difluprednate	Others	Others	82.23%	-5.38%
S2516	Xylazine HCl	Adrenergic Receptor	Adrenergic Receptor	82.23%	-5.38%
S2809	MPEP	GluR	GluR	82.23%	-5.38%
S4640	Lesinurad	Others	Others	82.30%	-5.45%
S4331	Meclocycline Sulfosalicylate	Others	Others	82.30%	-5.45%
S4235	Phenazopyridine	Sodium Channel	Sodium	82.30%	-5.45%
S3061	Epinephrine HCl	Adrenergic Receptor	Adrenergic Receptor	82.30%	-5.45%
S2667	Dolutegravir (GSK1349572)	Integrase	Integrase	82.30%	-5.45%
S4332	Medrysone	Others	Others	82.36%	-5.51%

S2442	Inosine	Others	Others	82.36%	-5.51%
S4772	alpha-Asarone	Others	Others	82.43%	-5.58%
S2104	Levosulpiride	Dopamine Receptor	Dopamine	82.43%	-5.58%
S4698	Vitamin K1	Osteocalcin	Osteocalcin	82.49%	-5.64%
S4393	Cephapirin Sodium	Others	Others	82.55%	-5.70%
S3858	Lawsone	Others	Others	82.55%	-5.70%
S2397	Palmatine chloride	Others	Others	82.55%	-5.70%
S3756	Methyl salicylate	TRPV	TRPV	82.62%	-5.77%
S3199	Reboxetine	Others	Others	82.68%	-5.83%
S1609	Methimazole	Others	Others	82.68%	-5.83%
S4661	Tiagabine	GABA Receptor	GABA	82.75%	-5.90%
S3208	Fexofenadine HCl	Histamine Receptor	Histamine	82.75%	-5.90%
S2565	Amoxicillin Sodium	Anti-infection	Anti-infection	82.75%	-5.90%
S3783	Echinacoside	Others	Others	82.88%	-6.03%
S4017	Allylthiourea	Others	Others	82.88%	-6.03%
S3100	2-Thiouracil	Others	Others	82.88%	-6.03%
S4720	Cefotaxime	Anti-infection	Anti-infection	82.94%	-6.09%
S4044	Toltrazuril	Anti-infection	Anti-infection	82.94%	-6.09%
S4179	Mezlocillin Sodium	Anti-infection	Anti-infection	82.94%	-6.09%
S2496	Ozagrel	P450 (e.g. CYP17)	P450 (e.g.	82.94%	-6.09%
S3742	Cholic acid	Others	Others	82.94%	-6.09%
S4009	Mirabegron	Adrenergic Receptor	Adrenergic Receptor	83.01%	-6.16%
S3015	Amoxicillin	Anti-infection	Anti-infection	83.01%	-6.16%
S4136	Carprofen	COX	COX	83.01%	-6.16%
S3106	Pidotimod	Immunology & Inflammation related	Immunology & Inflammation	83.01%	-6.16%
S4197	Bemegride	GABA Receptor	GABA	83.07%	-6.22%
S3772	5-	Others	Others	83.14%	-6.29%
S3172	Anagrelide HCl	PDE	PDE	83.14%	-6.29%
S1508	Alprostadil	Immunology & Inflammation related	Immunology & Inflammation	83.14%	-6.29%
S4025	Homatropine	AChR	AChR	83.20%	-6.35%
S3745	Balsalazide disodium	Immunology & Inflammation related	Immunology & Inflammation	83.20%	-6.35%
S4367	Suxibuzone	Others	Others	83.33%	-6.48%
S4820	Diastase	Others	Others	83.40%	-6.55%
S3147	Entacapone	Histone Methyltransferase	Histone Methyltransfera	83.40%	-6.55%
S3968	Histamine	Histamine Receptor	Histamine	83.46%	-6.61%
S1719	Zalcitabine	Reverse Transcriptase	Reverse Transcriptase	83.46%	-6.61%
S2789	Tofacitinib (CP-690550,Tasocitinib)	JAK	JAK	83.46%	-6.61%
S3656	Piribedil	Dopamine Receptor,Adrenergic Receptor	Dopamine Receptor,Adre nergic	83.46%	-6.61%
S4336	Metaraminol	Others	Others	83.53%	-6.68%
S4240	Doxylamine	Histamine Receptor	Histamine	83.53%	-6.68%
S1237	Temozolomide	DNA/RNA Synthesis,Autophag y	DNA/RNA Synthesis,Auto phagy	83.53%	-6.68%
S4112	Desvenlafaxine Succinate	Adrenergic Receptor,5-HT Receptor	Adrenergic Receptor,5-HT Receptor	83.72%	-6.87%
S2609	Inulin	Others	Others	83.72%	-6.87%
S4125	Sodium	HDAC	HDAC	83.72%	-6.87%
S4733	Retigabine	Potassium Channel	Potassium	83.79%	-6.94%
S3997	Oxaceprol	Immunology & Inflammation related	Immunology & Inflammation	83.79%	-6.94%

S4587	Pentylenetetrazol	GABA Receptor	GABA	83.85%	-7.00%
S3113	Pyridoxine HCl	Vitamin	Vitamin	83.85%	-7.00%
S4270	Oxiracetam	GABA Receptor	GABA	83.92%	-7.07%
S3758	Sinomenine hydrochloride	Immunology & Inflammation related,Autophagy	Immunology & Inflammation related,Autoph	83.92%	-7.07%
S3970	Vindoline	Microtubule Associated	Microtubule Associated	83.92%	-7.07%
S4177	Uracil	Others	Others	83.98%	-7.13%
S4224	Erythritol	Others	Others	83.98%	-7.13%
S3687	Urea	Others	Others	83.98%	-7.13%
S1162	PA-824	Anti-infection	Anti-infection	83.98%	-7.13%
S4326	Ethoxzolamide	Others	Others	84.05%	-7.20%
S4647	Cefmenoxime hydrochloride	Anti-infection	Anti-infection	84.05%	-7.20%
S3921	Lathyrol	Others	Others	84.05%	-7.20%
S2792	Torcetrapib	CETP	CETP	84.05%	-7.20%
S4522	Dehydroacetic acid	Anti-infection	Anti-infection	84.11%	-7.26%
S4841	Laurocapram	Others	Others	84.11%	-7.26%
S2586	Dimethyl Fumarate	Others	Others	84.11%	-7.26%
S2260	Amygdalin	Others	Others	84.11%	-7.26%
S3114	Vitamin C	Vitamin	Vitamin	84.18%	-7.33%
S4076	Propranolol HCl	Adrenergic Receptor	Adrenergic Receptor	84.24%	-7.39%
S1168	Valproic acid sodium salt (Sodium valproate)	GABA Receptor,HDAC,Autophagy	GABA Receptor,HDAC,Autophagy	84.24%	-7.39%
S4748	Ondansetron Hydrochloride Dihydrate	5-HT Receptor（高选择性和竞争性的 5-HT3 受体拮抗剂）	5-HT Receptor（高选择性和竞争性的 5-	84.30%	-7.45%
S4510	4-Aminobenzoic	Others	Others	84.30%	-7.45%
S1357	Lidocaine	Histamine Receptor	Histamine	84.30%	-7.45%
S4415	Misoprostol	Others	Others	84.37%	-7.52%
S4062	Ronidazole	Anti-infection	Anti-infection	84.37%	-7.52%
S4163	Doxycycline	MMP	MMP	84.37%	-7.52%
S2491	Nitrendipine	Calcium Channel,Autophagy	Calcium Channel,Autop	84.43%	-7.58%
S3930	Liquiritin	Others	Others	84.50%	-7.65%
S4141	Dinitolmide	Anti-infection	Anti-infection	84.56%	-7.71%
S3707	Ethopabate	Anti-infection	Anti-infection	84.56%	-7.71%
S4229	Oxybuprocaine HCl	Sodium Channel	Sodium	84.56%	-7.71%
S3849	D-Galactose	Others	Others	84.56%	-7.71%
S4542	Trihexyphenidyl hydrochloride	AChR	AChR	84.63%	-7.78%
S3616	Asiaticoside	ROS	ROS	84.63%	-7.78%
S3805	Stevioside	Others	Others	84.63%	-7.78%
S4185	Tiratricol	Others	Others	84.63%	-7.78%
S3956	Tetramethylpyrazine	Immunology & Inflammation related	Immunology & Inflammation	84.69%	-7.84%
S4058	Ropivacaine HCl	Sodium Channel	Sodium	84.82%	-7.97%
S4593	Chlormadinone acetate	Androgen Receptor,5-alpha Reductase	Androgen Receptor,5-alpha	84.95%	-8.10%
S4391	Camylofin	Others	Others	84.95%	-8.10%
S4206	Cysteamine HCl	Others	Others	84.95%	-8.10%
S3051	Bosentan Hydrate	Endothelin Receptor	Endothelin	84.95%	-8.10%
S2794	Sofosbuvir (PSI-7977, GS-7977)	DNA/RNA Synthesis	DNA/RNA Synthesis	84.95%	-8.10%
S3810	Scutellarin	Others	Others	85.02%	-8.17%
S1825	Erdosteine	Others	Others	85.02%	-8.17%

S4010	Acebutolol HCl	Adrenergic Receptor	Adrenergic Receptor	85.08%	-8.23%
S4050	Valganciclovir HCl	Anti-infection	Anti-infection	85.21%	-8.36%
S2602	Acemetacin	COX	COX	85.21%	-8.36%
S3773	Tyrosol	Others	Others	85.21%	-8.36%
S3996	Thioctic acid	Others	Others	85.28%	-8.43%
S4164	Doxofylline	PDE	PDE	85.34%	-8.49%
S4750	Sulfacetamide sodium salt hydrate	Autophagy,Anti-infection	Autophagy,Anti-infection	85.41%	-8.56%
S1398	Stavudine (d4T)	Reverse Transcriptase	Reverse Transcriptase	85.41%	-8.56%
S4330	Isoetharine	Others	Others	85.47%	-8.62%
S3775	Ligustrazine hydrochloride	Immunology & Inflammation related	Immunology & Inflammation	85.47%	-8.62%
S4701	2-Deoxy-D-glucose	Others	Others	85.54%	-8.69%
S2497	Pancuronium	AChR	AChR	85.54%	-8.69%
S4002	Sitagliptin phosphate	DPP-4	DPP-4	85.73%	-8.88%
S3644	Sulfamonomethoxi	Anti-infection	Anti-infection	85.80%	-8.95%
S3982	Batyl alcohol	Immunology & Inflammation related	Immunology & Inflammation	85.80%	-8.95%
S4138	Dropropizine	Others	Others	85.80%	-8.95%
S4834	Propantheline	AChR	AChR	85.86%	-9.01%
S1740	Guaifenesin	Others	Others	85.86%	-9.01%
S3193	Ticarcillin sodium	Anti-infection	Anti-infection	85.86%	-9.01%
S4099	Dexlansoprazole	Proton Pump	Proton Pump	85.93%	-9.08%
S1638	Ibuprofen	COX	COX	85.93%	-9.08%
S4149	Amfenac Sodium Monohydrate	COX	COX	85.99%	-9.14%
S3623	Ceftibuten	Anti-infection	Anti-infection	86.12%	-9.27%
S1965	Primidone	Sodium Channel	Sodium	86.12%	-9.27%
S4754	Betulin	Others	Others	86.25%	-9.40%
S4020	Sodium Picosulfate	Others	Others	86.38%	-9.53%
S4043	Tetrahydrozoline HCl	Adrenergic Receptor	Adrenergic Receptor	86.38%	-9.53%
S4042	Nafcillin Sodium	Anti-infection	Anti-infection	86.44%	-9.59%
S4246	Tranylcypromine (2-PCPA) HCl	MAO	MAO	86.44%	-9.59%
S3162	Tylosin tartrate	Anti-infection	Anti-infection	86.44%	-9.59%
S4707	Oleic Acid	Others	Others	86.51%	-9.66%
S4692	Succimer	Others	Others	86.51%	-9.66%
S3684	Methacholine	AChR	AChR	86.51%	-9.66%
S3693	2,6-	Others	Others	86.51%	-9.66%
S2455	Bethanechol	AChR	AChR	86.51%	-9.66%
S3927	Swertiamarin	Others	Others	86.57%	-9.72%
S4576	Sulfabenzamide	Anti-infection	Anti-infection	86.64%	-9.79%
S3663	Afloqualone	GABA Receptor	GABA	86.64%	-9.79%
S4521	DEET	Anti-infection	Anti-infection	86.70%	-9.85%
S4651	Etoricoxib	COX	COX	86.77%	-9.92%
S4023	Procaine HCl	NMDAR,Sodium Channel,AChR	NMDAR,Sodium	86.77%	-9.92%
S4581	Triacetin	Anti-infection	Anti-infection	86.83%	-9.98%
S3957	Gamma-Oryzanol	Others	Others	86.90%	-10.05%
S4375	Mepenzolate	Others	Others	86.90%	-10.05%
S3923	Ginsenoside Rg1	Others	Others	86.96%	-10.11%
S4520	2-Aminoheptane	Others	Others	87.03%	-10.18%
S4803	Thymidine	Others	Others	87.03%	-10.18%
S4664	Molsidomine	Others	Others	87.03%	-10.18%
S4666	Sivelestat sodium tetrahydrate	Serine Protease	Serine Protease	87.09%	-10.24%

S1511	Lactulose	Others	Others	87.09%	-10.24%
S2553	5-Aminolevulinic acid HCl	Others	Others	87.16%	-10.31%
S2159	Tebipenem Pivoxil	Anti-infection	Anti-infection	87.16%	-10.31%
S4404	Pasiniazid	Others	Others	87.29%	-10.44%
S4085	Levobetaxolol HCl	Adrenergic Receptor	Adrenergic Receptor	87.29%	-10.44%
S2585	Brompheniramine hydrogen maleate	Histamine Receptor	Histamine Receptor	87.35%	-10.50%
S2460	Pramipexole	Dopamine Receptor	Dopamine	87.35%	-10.50%
S1958	Sulbactam	Anti-infection	Anti-infection	87.35%	-10.50%
S4101	Voglibose	Others	Others	87.42%	-10.57%
S4833	Cefoxitin sodium	Anti-infection	Anti-infection	87.42%	-10.57%
S1567	Pomalidomide	TNF-alpha	TNF-alpha	87.55%	-10.70%
S4265	Nicaraven	Others	Others	87.61%	-10.76%
S2554	Daphnetin	PKA,EGFR,PKC	PKA,EGFR,PK	87.68%	-10.83%
S3195	Azlocillin sodium	Anti-infection	Anti-infection	87.80%	-10.95%
S4662	Atazanavir	HIV Protease	HIV Protease	87.87%	-11.02%
S3697	Mafenide	Carbonic Anhydrase	Carbonic	87.87%	-11.02%
S4022	Probenecid	TRPV	TRPV	87.93%	-11.08%
S4836	Nilutamide	Androgen Receptor	Androgen	88.00%	-11.15%
S3807	Dehydroandrograp	Chloride Channel	Chloride	88.13%	-11.28%
S4676	Gluconolactone	Others	Others	88.32%	-11.47%
S4736	Trapidil	PDGFR	PDGFR	88.39%	-11.54%
S3755	Betaine	Others	Others	88.39%	-11.54%
S4846	Meropenem	Anti-infection	Anti-infection	88.52%	-11.67%
S3866	Galanthamine	AChR	AChR	88.52%	-11.67%
S1750	Octocrylene	Others	Others	88.58%	-11.73%
S4122	Tilmicosin	Anti-infection	Anti-infection	88.65%	-11.80%
S3723	Ramosetron Hydrochloride	5-HT Receptor（5-HT3 受体拮抗剂，用于研究恶心和呕吐）	5-HT Receptor（5-HT3 受体拮抗剂，用于研究恶心和呕	88.65%	-11.80%
S4068	Tinidazole	Anti-infection	Anti-infection	88.65%	-11.80%
S4835	Aceclofenac	Immunology & Inflammation related	Immunology & Inflammation	88.71%	-11.86%
S1630	Allopurinol	ROS	ROS	88.71%	-11.86%
S4506	Acetazolamide	Carbonic Anhydrase	Carbonic	88.84%	-11.99%
S3788	Carvacrol	Others	Others	88.84%	-11.99%
S4266	Brimonidine Tartrate	Adrenergic Receptor	Adrenergic Receptor	88.91%	-12.06%
S2277	Caffeic Acid	Others	Others	88.91%	-12.06%
S4648	Dantrolene sodium hemiheptahydrate	Calcium Channel	Calcium Channel	88.97%	-12.12%
S4689	Deoxycholic acid	Others	Others	89.17%	-12.32%
S4152	Ethamsylate	Immunology & Inflammation related	Immunology & Inflammation	89.30%	-12.45%
S2549	Trospium chloride	AChR	AChR	89.30%	-12.45%
S4714	(-)-Menthol	Opioid Receptor	Opioid	89.36%	-12.51%
S4312	Carbadox	Others	Others	89.49%	-12.64%
S4658	Hydroquinidine	Others	Others	89.49%	-12.64%
S3925	(-)-Epicatechin	Others	Others	89.49%	-12.64%
S3969	Veratric acid	Immunology & Inflammation related	Immunology & Inflammation	89.49%	-12.64%
S4636	Teneligliptin hydrobromide	DPP-4	DPP-4	89.55%	-12.70%
S4357	Tacrine HCl	Others	Others	89.62%	-12.77%
S4562	Dehydrocholic acid	Others	Others	89.68%	-12.83%
S2502	Quinine HCl	Potassium Channel	Potassium	89.68%	-12.83%
S2505	Rosiglitazone	PPAR	PPAR	89.81%	-12.96%

S4527	Fenofibric acid	PPAR	PPAR	89.81%	-12.96%
S3657	Promestriene	Estrogen/progestogen Receptor	Estrogen/progestogen	89.81%	-12.96%
S4620	Cefuroxime sodium	Anti-infection	Anti-infection	89.88%	-13.03%
S4558	Citiolone	Others	Others	89.88%	-13.03%
S3153	levalbuterol tartrate	Adrenergic Receptor	Adrenergic Receptor	89.88%	-13.03%
S4135	Clorprenaline HCl	Adrenergic Receptor	Adrenergic Receptor	90.01%	-13.16%
S4830	Maltose	Others	Others	90.14%	-13.29%
S4556	Carzenide	Others	Others	90.14%	-13.29%
S3176	Betahistine 2HCl	Histamine Receptor	Histamine	90.14%	-13.29%
S4695	D panthenol	Vitamin	Vitamin	90.27%	-13.42%
S4024	Homatropine Methylbromide	AChR	AChR	90.27%	-13.42%
S4293	Promethazine HCl	Histamine Receptor	Histamine	90.33%	-13.48%
S4088	Flumethasone	Glucocorticoid Receptor	Glucocorticoid Receptor	90.40%	-13.55%
S4219	Azaperone	Dopamine Receptor	Dopamine	90.40%	-13.55%
S4339	Meticrane	Others	Others	90.40%	-13.55%
S3071	Vanillin	Others	Others	90.53%	-13.68%
S4517	Cefotaxime sodium	Anti-infection	Anti-infection	90.66%	-13.81%
S4092	Pramoxine HCl	Others	Others	90.66%	-13.81%
S2601	Gliclazide	Potassium Channel	Potassium	90.66%	-13.81%
S4123	Timolol Maleate	Adrenergic Receptor	Adrenergic Receptor	90.72%	-13.87%
S4175	Sulfaguanidine	Anti-infection	Anti-infection	90.79%	-13.94%
S4725	Benzenesulfonami	Carbonic Anhydrase	Carbonic	90.85%	-14.00%
S4366	Pinacidil	Others	Others	90.85%	-14.00%
S1902	Vitamin B12	Vitamin	Vitamin	90.85%	-14.00%
S2489	Nateglinide	Potassium Channel	Potassium	90.92%	-14.07%
S2311	Hyodeoxycholic acid (HDCA)	Others	Others	91.05%	-14.20%
S4600	Benzyl alcohol	Others	Others	91.11%	-14.26%
S4041	Olsalazine Sodium	Anti-infection	Anti-infection	91.11%	-14.26%
S4691	Oxybenzone	Others	Others	91.18%	-14.33%
S4368	Carbenoxolone	Others	Others	91.24%	-14.39%
S4003	Lithocholic acid	FXR	FXR	91.30%	-14.45%
S4213	Dirithromycin	Anti-infection	Anti-infection	91.37%	-14.52%
S3944	Valproic acid	HDAC	HDAC	91.43%	-14.58%
S4650	Atipamezole	Adrenergic Receptor	Adrenergic Receptor	91.63%	-14.78%
S4304	Anisindione	Others	Others	91.63%	-14.78%
S4222	Piperacillin Sodium	Anti-infection	Anti-infection	91.63%	-14.78%
S3627	Tryptamine	TAAR	TAAR	91.63%	-14.78%
S2566	Isoprenaline HCl	Adrenergic Receptor	Adrenergic Receptor	91.76%	-14.91%
S3850	Glucosamine	Others	Others	91.76%	-14.91%
S4070	Guanidine HCl	Others	Others	91.95%	-15.10%
S3719	Topiroxostat	Others	Others	91.95%	-15.10%
S3980	Pyridoxine	Vitamin	Vitamin	92.02%	-15.17%
S2457	Clindamycin HCl	Anti-infection	Anti-infection	92.02%	-15.17%
S2511	Sulfadoxine	Anti-infection	Anti-infection	92.02%	-15.17%
S4652	Sulisobenzone	Others	Others	92.08%	-15.23%
S2494	Olopatadine HCl	Histamine Receptor	Histamine	92.08%	-15.23%
S4660	Glycopyrrolate	AChR	AChR	92.15%	-15.30%
S4850	Flucloxacillin	Anti-infection	Anti-infection	92.15%	-15.30%
S4847	Faropenem	Anti-infection	Anti-infection	92.21%	-15.36%
S4570	Halothane	Others	Others	92.21%	-15.36%
S3037	Bepotastine	Histamine Receptor	Histamine	92.21%	-15.36%

S4004	Ethambutol 2HCl	Anti-infection	Anti-infection	92.28%	-15.43%
S3794	Palmitic acid	Others	Others	92.34%	-15.49%
S4376	Aceclidine HCl	Others	Others	92.34%	-15.49%
S4131	Levodropropizine	Histamine Receptor	Histamine	92.34%	-15.49%
S3985	Methyl 4-hydroxybenzoate	Others	Others	92.41%	-15.56%
S3638	Cefamandole	Anti-infection	Anti-infection	92.41%	-15.56%
S4693	Guanfacine Hydrochloride	Adrenergic Receptor	Adrenergic Receptor	92.54%	-15.69%
S3856	Allantoin	Others	Others	92.54%	-15.69%
S4655	Sulpiride	Dopamine Receptor	Dopamine	92.60%	-15.75%
S4073	Sodium 4-Aminosalicylate	NF-κB	NF-κB	92.73%	-15.88%
S3851	Camphor	Others	Others	92.80%	-15.95%
S4128	Troxipide	Others	Others	92.80%	-15.95%
S2589	Miglitol	Others	Others	92.86%	-16.01%
S3971	Fusidine	Anti-infection	Anti-infection	92.86%	-16.01%
S2557	Terbinafine HCl	Anti-infection	Anti-infection	92.86%	-16.01%
S4649	Atipamezole hydrochloride	Adrenergic Receptor	Adrenergic Receptor	92.93%	-16.08%
S3729	Iron sucrose	Others	Others	92.93%	-16.08%
S4509	4-Aminoantipyrine	Others	Others	92.99%	-16.14%
S4516	(+)-Camphor	TRPV	TRPV	93.05%	-16.20%
S4512	Aceglutamide	Others	Others	93.05%	-16.20%
S4641	Tedizolid	Anti-infection	Anti-infection	93.12%	-16.27%
S3979	Zinc Undecylenate	Anti-infection	Anti-infection	93.12%	-16.27%
S4759	p-Coumaric Acid	Anti-infection	Anti-infection	93.18%	-16.33%
S4843	Potassium acetate	Anti-infection	Anti-infection	93.18%	-16.33%
S4170	Coumarin	Immunology & Inflammation related	Immunology & Inflammation	93.18%	-16.33%
S3950	Maltitol	Others	Others	93.18%	-16.33%
S4119	Pefloxacin Mesylate Dihydrate	Topoisomerase	Topoisomerase	93.18%	-16.33%
S4546	Xylitol	Others	Others	93.25%	-16.40%
S4344	Oxprenolol HCl	Others	Others	93.25%	-16.40%
S3881	Scopoletin	Immunology & Inflammation related	Immunology & Inflammation	93.38%	-16.53%
S3909	Catalpol	Others	Others	93.44%	-16.59%
S4257	Alizapride HCl	Dopamine Receptor	Dopamine	93.44%	-16.59%
S4211	Montelukast	LTR	LTR	93.57%	-16.72%
S2590	Pioglitazone	PPAR	PPAR	93.57%	-16.72%
S3992	D-(+)-Trehalose dihydrate	Others	Others	93.77%	-16.92%
S4244	Serotonin HCl	5-HT Receptor（内源性 5-HT 受体激动剂）	5-HT Receptor（内源性 5-HT 受体激动剂）	93.77%	-16.92%
S4667	Lidocaine	EGFR	EGFR	93.83%	-16.98%
S4568	Ethylvanillin	Others	Others	94.22%	-17.37%
S2573	Tetracaine HCl	Calcium Channel	Calcium	94.22%	-17.37%
S4047	Bisacodyl	Others	Others	94.29%	-17.44%
S3178	Brinzolamide	Carbonic Anhydrase	Carbonic	94.29%	-17.44%
S4345	Pentoxifylline	Others	Others	94.42%	-17.57%
S4679	Terazosin HCl	Adrenergic Receptor	Adrenergic Receptor	94.42%	-17.57%
S4539	Salicylic acid	COX	COX	94.48%	-17.63%
S4308	Benzthiazide	Others	Others	94.55%	-17.70%
S3754	4-Hydroxybenzoic	Others	Others	94.55%	-17.70%
S4848	Dalbavancin	Anti-infection	Anti-infection	94.68%	-17.83%
S2765	MK-2048	Integrase	Integrase	94.87%	-18.02%
S4538	Pantoprazole	Proton Pump	Proton Pump	94.93%	-18.08%

S2551	Sulbactam sodium	Anti-infection	Anti-infection	95.13%	-18.28%
S4657	Eslicarbazepine	Others	Others	95.19%	-18.34%
S4563	Diatrizoic acid	Others	Others	95.19%	-18.34%
S4532	Iopamidol	Others	Others	95.19%	-18.34%
S4669	Benzocaine hydrochloride	Sodium Channel	Sodium Channel	95.32%	-18.47%
S4524	2-Ethoxybenzamide	Immunology & Inflamm	Immunology & Inflamm	95.52%	-18.67%
S4382	Pyrilamine Maleate	Others	Others	95.52%	-18.67%
S2283	Cinchonine(LA402	Others	Others	95.52%	-18.67%
S1504	Dyphylline	PDE	PDE	95.78%	-18.93%
S4696	CarbinoxaMine	Histamine Receptor	Histamine	95.84%	-18.99%
S3975	Protocatechuic acid	Others	Others	95.84%	-18.99%
S4201	Florfenicol	Anti-infection	Anti-infection	95.84%	-18.99%
S3612	Rosmarinic acid	IκB/IKK	IκB/IKK	95.91%	-19.06%
S4387	Bendroflumethiazid	Others	Others	95.97%	-19.12%
S3977	(-)-Borneol	Others	Others	95.97%	-19.12%
S4813	Cefuroxime axetil	Anti-infection	Anti-infection	95.97%	-19.12%
S4090	Fenspiride HCl	PDE	PDE	96.17%	-19.32%
S4637	Prasugrel	P2 Receptor	P2 Receptor	96.30%	-19.45%
S4717	Isatin	MAO	MAO	96.75%	-19.90%
S4656	Parecoxib	COX	COX	96.81%	-19.96%
S3799	Gentisic acid	Immunology & Inflammation related	Immunology & Inflammation	96.81%	-19.96%
S4316	Clopamide	Others	Others	96.88%	-20.03%
S3870	D-Pinitol	Others	Others	96.88%	-20.03%
S4014	Hyoscyamine	AChR	AChR	96.94%	-20.09%
S4697	Saxagliptin hydrate	DPP-4	DPP-4	97.01%	-20.16%
S4819	Saccharin	Others	Others	97.07%	-20.22%
S3046	Azilsartan	RAAS	RAAS	97.20%	-20.35%
S4663	Fusidate Sodium	Anti-infection	Anti-infection	97.33%	-20.48%
S4626	Ethosuximide	Calcium Channel	Calcium	97.33%	-20.48%
S4365	Phthalylsulfacetami	Others	Others	97.33%	-20.48%
S4124	Tolazoline HCl	Adrenergic Receptor	Adrenergic Receptor	97.33%	-20.48%
S4594	Cephalothin	Anti-infection	Anti-infection	97.46%	-20.61%
S4247	Prucalopride Succinate	5-HT Receptor（5-HT 4 受体激动剂）	5-HT Receptor（5-HT 4 受体	97.46%	-20.61%
S3750	Sodium benzoate	Others	Others	97.59%	-20.74%
S4299	Dicoumarol	Others	Others	97.66%	-20.81%
S2613	Clorsulon	Anti-infection	Anti-infection	97.79%	-20.94%
S2116	Conivaptan HCl	Vasopressin Receptor	Vasopressin Receptor	97.92%	-21.07%
S4792	N-Acetylneuraminic	Others	Others	97.98%	-21.13%
S4361	Cinoxacin	Others	Others	98.05%	-21.20%
S2484	Milrinone	PDE	PDE	98.05%	-21.20%
S4595	Cefazolin Sodium	Anti-infection	Anti-infection	98.11%	-21.26%
S4338	Methoxamine HCl	Others	Others	98.43%	-21.58%
S3639	Tacrine hydrochloride	AChR	AChR	98.43%	-21.58%
S4534	6-Acetamidohexanoic	Others	Others	98.56%	-21.71%
S4769	L-5-	Others	Others	98.63%	-21.78%
S4530	i-Inositol	Others	Others	98.69%	-21.84%
S4749	Citalopram HBr	5-HT Receptor（5-羟色胺再摄取抑制剂）	5-HT Receptor（5-羟色胺再摄取抑制剂）	98.69%	-21.84%
S3008	Zaltoprofen	COX	COX	98.76%	-21.91%
S4514	Acetylleucine	Others	Others	98.89%	-22.04%
S3637	Cefpirome sulfate	Anti-infection	Anti-infection	99.21%	-22.36%

S4385	Fosfomycin Tromethamine	Others	Others	99.28%	-22.43%
S4753	Ganoderic acid A	Immunology & Inflammation related	Immunology & Inflammation	99.41%	-22.56%
S4678	Povidone iodine	Anti-infection	Anti-infection	99.54%	-22.69%
S4388	Bentiromide	Others	Others	99.67%	-22.82%
S3885	Pyrogallol	ROS	ROS	99.67%	-22.82%
S2615	Noradrenaline bitartrate	Adrenergic Receptor	Adrenergic Receptor	99.80%	-22.95%
S4515	Ademetionine disulfate tosylate	Others	Others	99.80%	-22.95%
S4322	Disopyramide Phosphate	Others	Others	99.93%	-23.08%
S4844	Cefcapene Pivoxil Hydrochloride	Anti-infection	Anti-infection	99.93%	-23.08%
S2559	Cortisone acetate	Glucocorticoid Receptor	Glucocorticoid Receptor	99.93%	-23.08%
S4625	Alcaftadine	Histamine Receptor	Histamine	99.99%	-23.14%
S4618	Fenoldopam	Dopamine Receptor	Dopamine	100.18%	-23.33%
S4687	Rivastigmine	AChR	AChR	100.18%	-23.33%
S4297	Mupirocin	DNA/RNA Synthesis	DNA/RNA	100.25%	-23.40%
S4716	Evans Blue	GluR	GluR	100.31%	-23.46%
S4752	Corticosterone	Glucocorticoid Receptor	Glucocorticoid Receptor	100.44%	-23.59%
S4525	Ethylparaben	Anti-infection	Anti-infection	100.44%	-23.59%
S4815	L-Cysteine HCl	Others	Others	100.51%	-23.66%
S4295	Meclofenamate	COX	COX	100.57%	-23.72%
S4682	Loxoprofen	Immunology & Inflammation related	Immunology & Inflammation	100.70%	-23.85%
S4817	Atenolol	Adrenergic Receptor	Adrenergic Receptor	100.77%	-23.92%
S4722	(+)-Catechin	ROS	ROS	100.90%	-24.05%
S3901	Astragaloside IV	Others	Others	100.96%	-24.11%
S4553	Bronopol	Anti-infection	Anti-infection	100.96%	-24.11%
S2851	Baricitinib (LY3009104,	JAK	JAK	100.96%	-24.11%
S4279	Demeclocycline	Anti-infection	Anti-infection	101.03%	-24.18%
S4723	(-)Epicatechin	ROS	ROS	101.16%	-24.31%
S4277	Bambuterol HCl	Adrenergic Receptor	Adrenergic Receptor	101.29%	-24.44%
S4503	Calcium D-	Vitamin	Vitamin	101.29%	-24.44%
S4278	Carteolol HCl	Adrenergic Receptor	Adrenergic Receptor	101.35%	-24.50%
S4550	Azelaic acid	Anti-infection	Anti-infection	101.55%	-24.70%
S4779	Menadiol Diacetate	Vitamin	Vitamin	101.61%	-24.76%
S4301	(R)-(+)-Atenolol	0	0	101.80%	-24.95%
S4548	Aminoguanidine hydrochloride	NOS	NOS	102.00%	-25.15%
S3974	(+)-Catechin	Others	Others	102.26%	-25.41%
S4171	Choline Chloride	AChR	AChR	102.32%	-25.47%
S4816	Diatrizoate sodium	Others	Others	102.78%	-25.93%
S2486	Moroxydine HCl	Anti-infection	Anti-infection	103.17%	-26.32%
S4564	Diethylcarbamazin e citrate	Anti-infection	Anti-infection	103.30%	-26.45%
S4267	Diacerein	IL Receptor	IL Receptor	103.49%	-26.64%
S4768	Melibiose	Others	Others	103.68%	-26.83%
S4849	Levocetirizine Dihydrochloride	Histamine Receptor	Histamine Receptor	103.81%	-26.96%
S4628	(+/-)-	COX	COX	104.40%	-27.55%

S4294	Procainamide HCl	DNA Methyltransferase,Sodium Channel	DNA Methyltransferase.Sodium	104.66%	-27.81%
S2556	Rosiglitazone	PPAR	PPAR	105.05%	-28.20%
S4735	Salvianolic acid B	Sirtuin	Sirtuin	105.50%	-28.65%
S4596	Cefixime	Anti-infection	Anti-infection	105.63%	-28.78%
S4726	Lauric Acid	Anti-infection	Anti-infection	105.89%	-29.04%
S4832	Tolmetin	Immunology & Inflammation related	Immunology & Inflammation	105.89%	-29.04%
S4526	Fenbufen	COX	COX	106.15%	-29.30%
S4289	Metoclopramide	Dopamine Receptor	Dopamine	106.28%	-29.43%
S4305	Anisotropine Methylbromide	Others	Others	106.67%	-29.82%
S4599	Benzyl benzoate	Others	Others	106.67%	-29.82%
S1983	Adenine HCl	DNA/RNA Synthesis	DNA/RNA	107.51%	-30.66%
S4623	Methylbenactyzine Bromide	AChR	AChR	109.26%	-32.41%
S2664	Clinofibrate	HMG-CoA Reductase	HMG-CoA Reductase	109.52%	-32.67%
S4335	Metaproterenol	Others	Others	109.71%	-32.86%
S4686	Vitamin E	COX,VEGFR	COX,VEGFR	110.10%	-33.25%
S4629	Chlorotrianisene	Estrogen/progestogen Receptor	Estrogen/progestogen	110.30%	-33.45%
S4148	Ampicillin	Anti-infection	Anti-infection	110.43%	-33.58%
S3207	Iopromide	Others	Others	111.59%	-34.74%
S3066	Naloxone HCl	Opioid Receptor	Opioid	111.79%	-34.94%
S3057	Azilsartan	RAAS	RAAS	113.47%	-36.62%
S4634	Sodium	Anti-infection	Anti-infection	115.22%	-38.37%
S4630	Diazoxide	Potassium Channel	Potassium	118.85%	-42.00%
S4633	Isosorbide	Others	Others	120.73%	-43.88%
S4291	Labetalol HCl	Adrenergic Receptor	Adrenergic Receptor	127.99%	-51.14%

Supplementary Table 5. The characterization and IC50 of sorafenib in different HCC cell lines

No.	HCC cell lines	Ethnicity	Sorafenib-IC50(μM)	CTNNB1 mutation	Defined
1	PLC/PRF/5	African	2.937	no	relatively sorafenib-sensitive
2	Huh7	Asian	3.447	no	
3	Hep3B	African-American	4.975	no	
4	HepG2	European	5.175		moderately sensitive
5	HLE	Asian	6.174	no	
6	MHCC-97L	Asian	7.926		relatively sorafenib-resistant
7	MHCC-97H	Asian	9.089	Mutation(In-Frame)	
8	MHCC-LM3	Asian	10.61		
9	SNU387	Asian	9.93	no	
10	HCC-P	Asian	9.08		

Supplementary Table 6. The clinical information and drug testing results of MDTs from 37 HCC patients

Number for RNA-seq	Number for experiments	Patient ID	pathological number	Sorafenib	PRI-724	Sora+PRI	SP>S	SP>P	Type	Gender	Cirrhosis	Tumor size	Microvascular invasion	BCLC stage	AFP	Tbil	ALB	ALP	ALT	AST	HBV	HCV	HAV
1	P132	503180	529643	✓	✓	✓	✗	✗	HCC	female	0	5	0	A	794	11.7	43.2	113	18	20	1	0	1
2	P134	551091	531682	✓	✓	✓	✓	✗	HCC	male	1	5	1	C	186.3	27.8	37.5	59	135	91	1	0	1
3	P123	526913	517630	✗	✗	✓	✓	✓	HCC	male	0	7	1	C	1150	11.7	40.3	81	20	30	0	0	1
4	P125	528003	518118	✓	✓	✓	✓	✗	ICC	male	0	6	0	A	1.92	9.5	38.1	195	14	18	0	0	1
5	P128	532494	520266	✓	✓	✓	✗	✗	HCC	female	1	6	0	A	46.51	20.9	35.1	186	181	195	0	1	1
6	P146	566495	539015	✗	✓	✓	✗	✗	HCC	male	1	3.5	1	C	4.7	36.5	49.1	81	22	24	1	0	1
7	P107	537904	523289	✗	✓	✓	✓	✗	ICC	male	0	5	0	A	1.6	15.3	43.2	68	15	25	0	0	1
8	P138	554859	532124	✓	✓	✓	✗	✓	ICC	female	0	3	0	A	6.62	11.7	42.2	241	53	42	0	0	1
9	P144	565287	537768	✗	✓	✓	✓	✓	HCC	female	1	1.2	0	A	165.3	14.4	41	88	41	41	1	0	1
10	P106	520755	514796	✗	✗	✗	✗	✗	HCC	female	1	5	0	A	>1210	22.8	43.8	108	20	23	1	0	1
11	P108	522550	515651	✗	✗	✗	✗	✗	HCC	male	1	10	1	A	>1210	22.3	44.2	117	23	40	1	0	1
12	P38	506122	507123	✗	✗	✗	✗	✗	ICC	female	0	10	0	A	11.16	15.4	49	1000	70	70	0	0	1
13	P121	479242	494095	✗	✗	✗	✗	✗	HCC	male	1	2.3	1	C	6.99	14.5	40.1	134	17	27	1	0	1
14	P122	526565	517302	✗	✗	✗	✗	✗	HCC	female	0	4	1	C	977.6	12.2	40.2	121	12	23	1	0	1
15	P124	527849	518063	✗	✗	✗	✗	✗	HCC	female	0	3	1	C	>1210	9.8	45.4	72	15	17	0	0	1
16	P126	327301	518475	✗	✗	✗	✗	✗	HCC	male	1	8	1	C	5.57	23	37.9	293	312	338	1	0	1
17	P127	532192	520222	✗	✗	✗	✗	✗	HCC	male	1	2	0	A	47.8	11.7	43.3	83	14	18	1	0	1
18	P129	532018	520681	✗	✗	✗	✗	✗	HCC	male	1	1.5	0	A	18.26	17.1	40.4	85	44	34	1	0	1
19	P54	510743	509629	✗	✗	✗	✗	✗	HCC	male	0	5.5	0	A	6.49	17.2	42.1	132	38	41	1	0	1
20	P130	545936	527478	✗	✗	✗	✗	✗	HCC	male	1	5.5	0	A	98.25	13	41.6	79	105	68	1	0	1
21	P131	549655	529157	✗	✗	✗	✗	✗	HCC	male	1	4	0	A	1.85	18.8	44.5	51	20	20	1	0	1
22	P133	551815	531037	✗	✗	✗	✗	✗	HCC	male	1	8	1	C	>1210	24.8	44.1	101	34	32	1	0	1
23	P135	505614	531765	✗	✗	✗	✗	✗	HCC	male	1	11	1	C	70.87	20.8	44.5	200	17	23	1	0	1
24	P136	553664	531988	✗	✗	✗	✗	✗	HCC	male	0	2.5	0	A	4.61	11	44.5	79	22	30	0	0	1
25	P137	571642	532893	✗	✗	✗	✗	✗	HCC	male	1	3	1	C	2.18	13.5	41.4	90	41	22	1	0	1
26	P139	557771	533878	✗	✗	✗	✗	✗	HCC	male	1	2.5	1	C	1.91	14.9	45	68	10	34	0	1	1
27	P142	565075	537766	✗	✗	✗	✗	✗	HCC	male	0	2	0	A	1.38	14.4	46.6	97	15	18	1	0	1
28	P143	565319	537815	✗	✗	✗	✗	✗	HCC	male	1	7	1	C	51.29	6	36.8	113	26	29	1	0	0
29	P145	566896	538534	✗	✗	✗	✗	✗	HCC	male	0	8.5	1	C	61.63	25.3	37.6	109	47	31	1	0	1
30	P147	567637	539048	✗	✗	✗	✗	✗	HCC	female	0	7	0		2.01	11.5	34.9	98	15	16	0	1	1
31	P148	523818	516257	✗	✗	✗	✗	✗	CHC	male	1	3	0	A	92.85	25.5	38.8	110	49	67	1	0	1
32	P149	568328	539536	✗	✗	✗	✗	✗	HCC	male	0	8	1	A	5.57	12.6	39.8	88	16	15	0	0	1
33	P150	569516	539769	✗	✗	✗	✗	✗	HCC	male	0	3.5	1	C	5.34	18	46	70	23	22			
34	P151	569932	539959	✗	✗	✗	✗	✗	ICC	male	0	2.5	0	A	2.28	17.8	47.1	78	20	17	0	0	1
35	P152	572506	542221	✗	✗	✗	✗	✗	HCC	male	1	3.3	1	A	1.44	22	42.1	81	38	26	0	0	1
36	P153	575117	542780	✗	✗	✗	✗	✗	HCC	male	0	8	1	C	2.32	2.2	41.7	125	30	43	1	0	1
37	P154	573666	542951	✗	✗	✗	✗	✗	HCC	male	1	4.5	0	A	2.92	15	45.4	71	25	27	1	0	1
	Response rates			5/37(13.5%)	8/37(21.6%)	9/37(24.3%)	5/37(13.5%)	3/37(8.1%)															
