# Supplementary material for: Inhibition of Wnt/β-catenin increases anti-tumor activity by synergizing with sorafenib in hepatocellular carcinoma
Source: Cell Death Dis. 2025 Jul 1;16(1):466. doi: 10.1038/s41419-025-07789-5 (PMC12216529; doi:10.1038/s41419-025-07789-5)
Supplement: Supplementary file 2 — Full and uncropped western blots - source data [file 41419_2025_7789_MOESM2_ESM.pdf]

Figure 2H

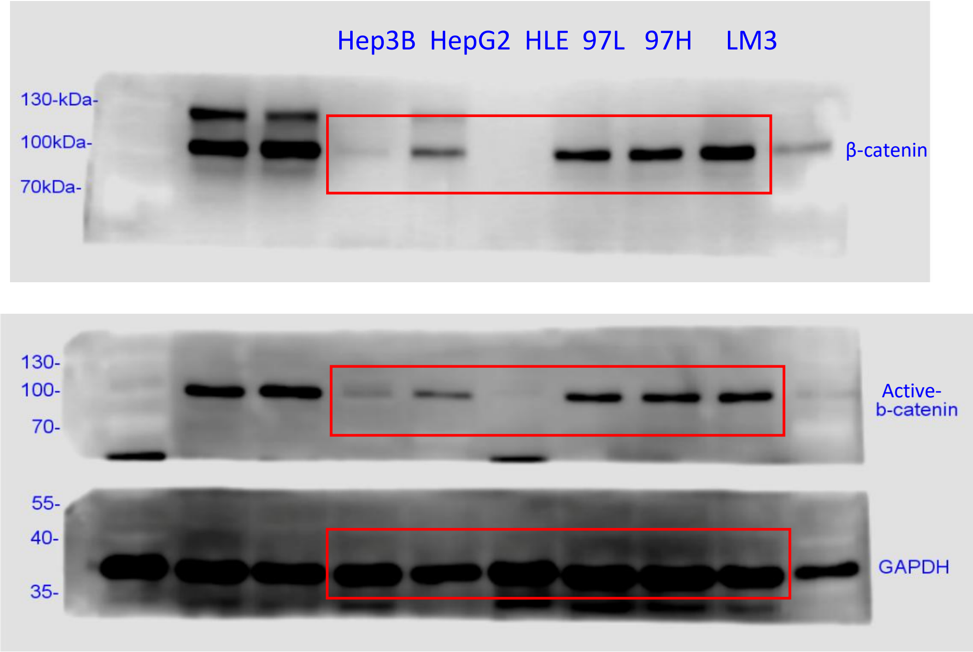

Hep3B HepG2 HLE 97L 97H LM3
130-kDa-
100kDa-
70kDa-
β-catenin
130-
100-
70-
Active-
b-catenin
55-
40-
35-
GAPDH

Figure 3A

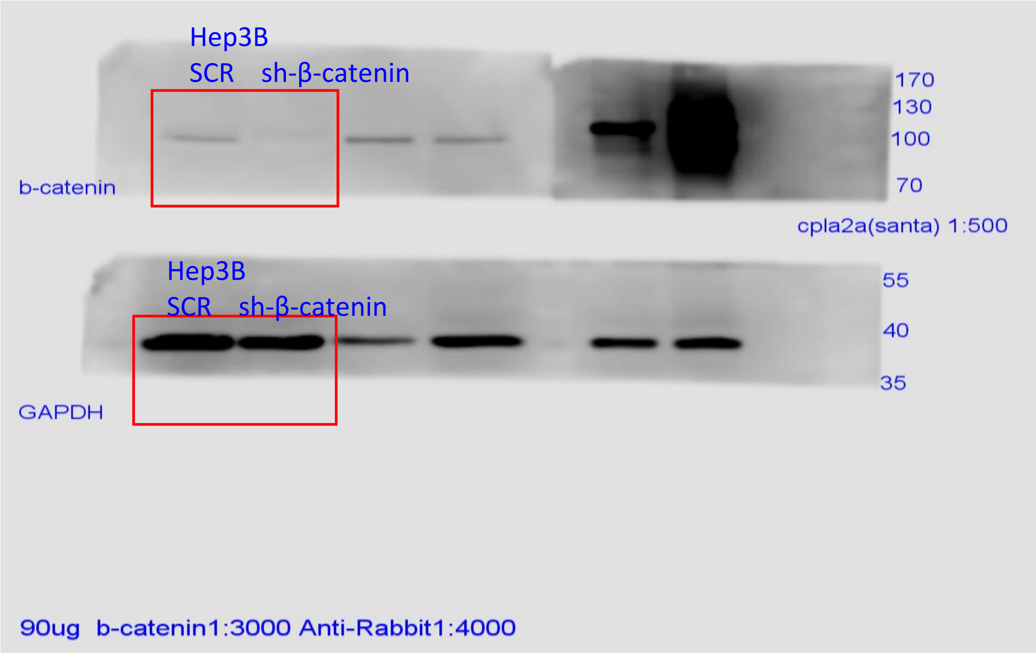

Figure 3B

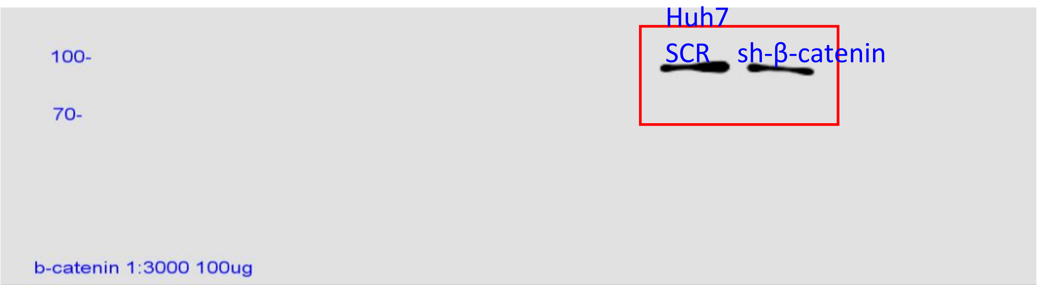

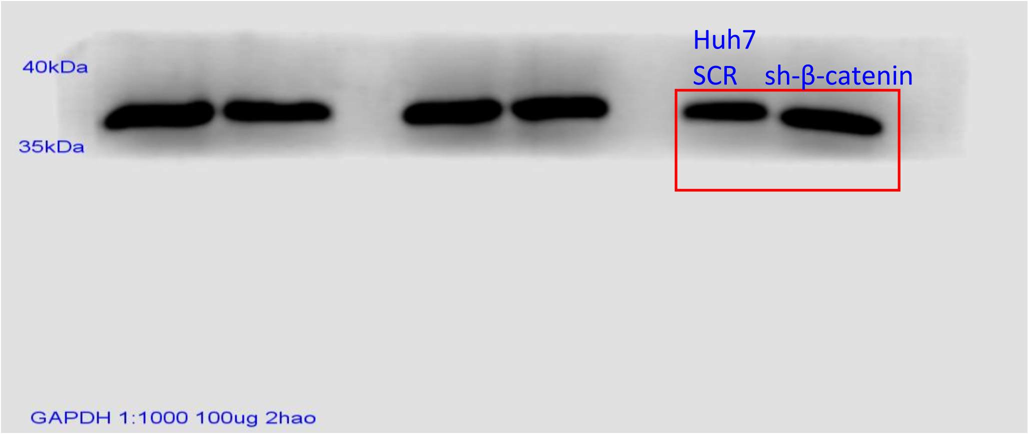
Huh7
SCR sh-β-catenin
40kDa
35kDa
GAPDH 1:1000 100ug 2hao

Figure 3C

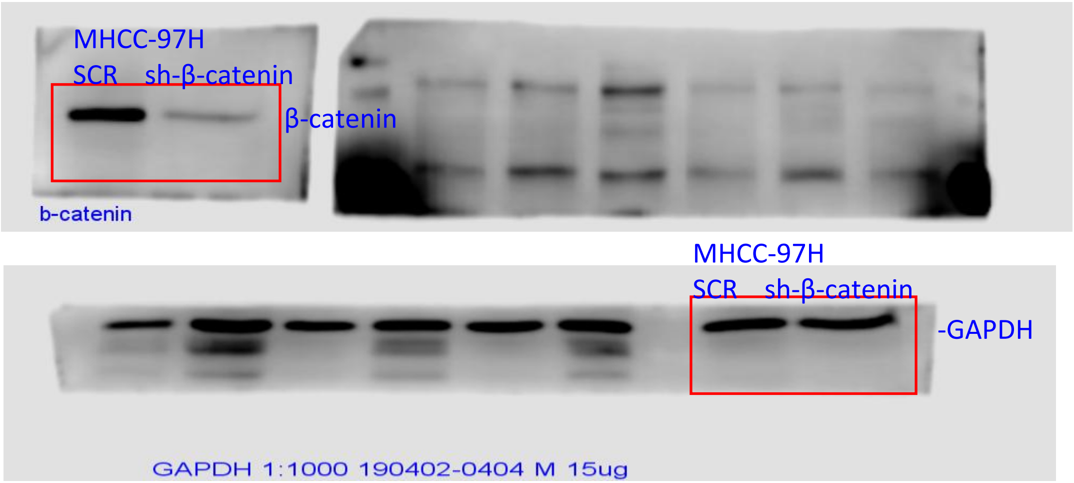
MHCC-97H
SCR sh-β-catenin
β-catenin
b-catenin
MHCC-97H
SCR sh-β-catenin
-GAPDH
GAPDH 1:1000 190402-0404 M 15ug

Figure 5C

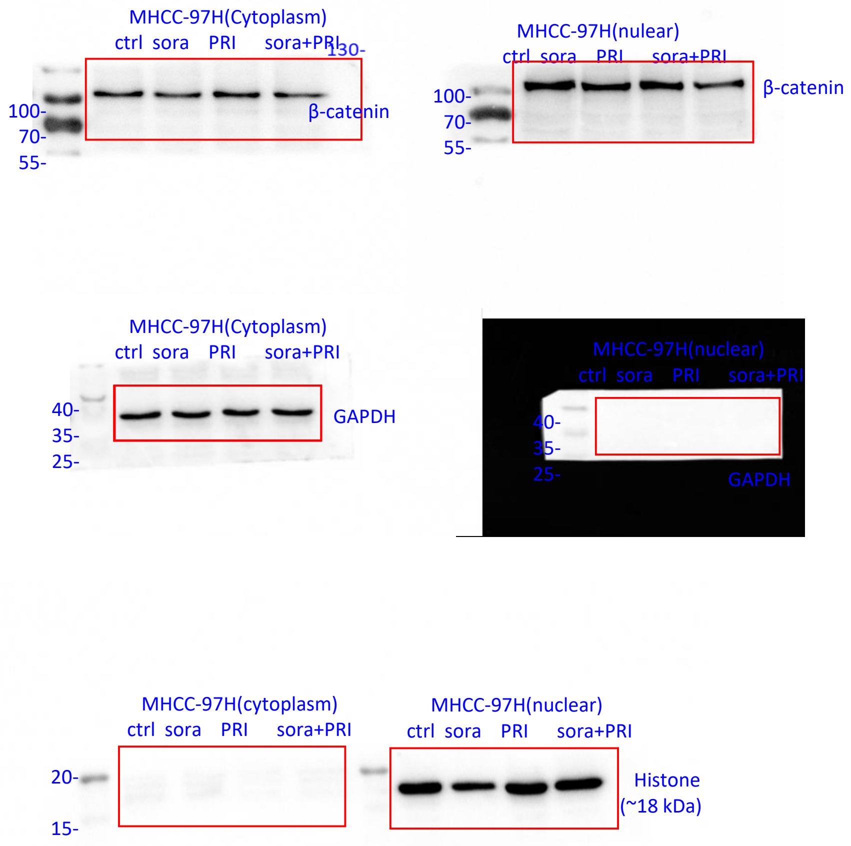
MHCC-97H(Cytoplasm)
ctrl sora PRI sora+PRI
130-
100-
70-
55-
β-catenin
MHCC-97H(nulear)
ctrl sora PRI sora+PRI
100-
70-
55-
β-catenin
MHCC-97H(Cytoplasm)
ctrl sora PRI sora+PRI
40-
35-
25-
GAPDH
MHCC-97H(nuclear)
ctrl sora PRI sora+PRI
40-
35-
25-
GAPDH
MHCC-97H(cytoplasm)
ctrl sora PRI sora+PRI
20-
15-
MHCC-97H(nuclear)
ctrl sora PRI sora+PRI
Histone
(~18 kDa)

Figure 5F

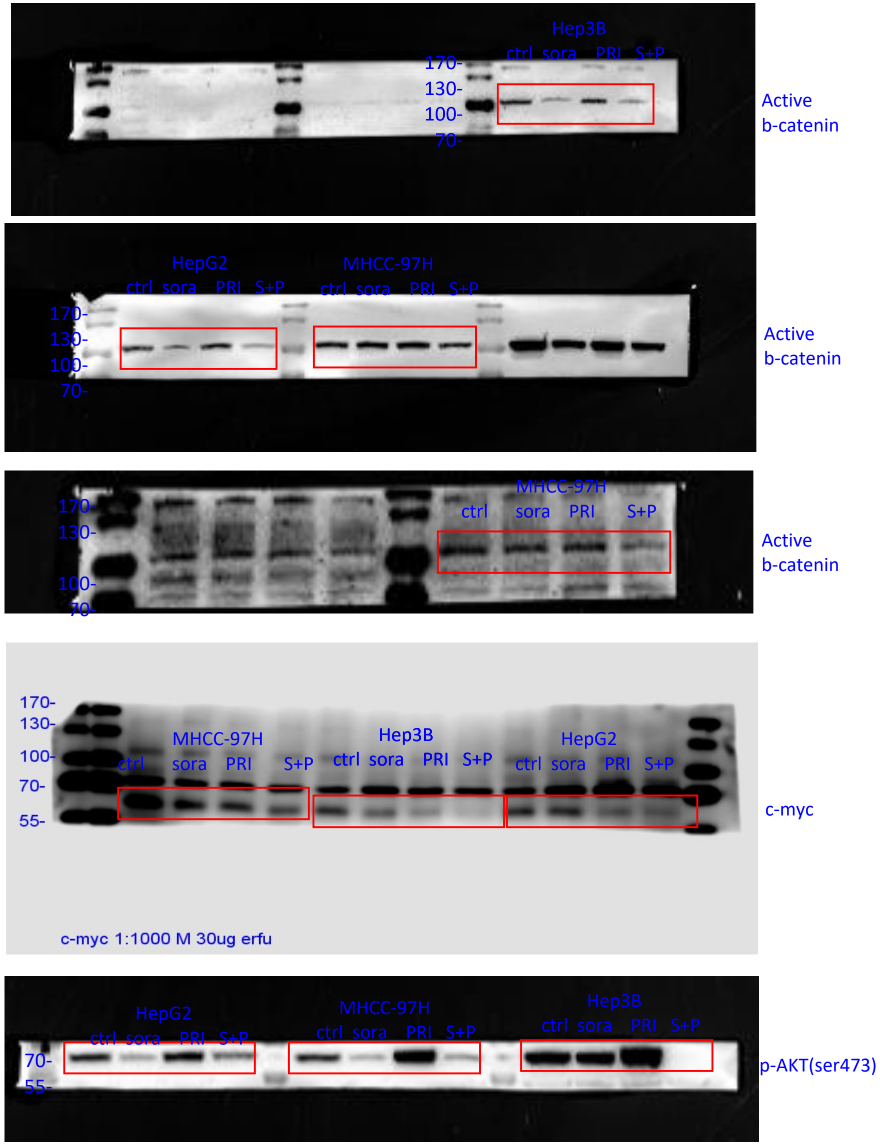
Hep3B
ctrl sora PRI S+P
170-
130-
100-
70-
Active b-catenin
HepG2
ctrl sora PRI S+P
MHCC-97H
ctrl sora PRI S+P
170-
130-
100-
70-
Active b-catenin
MHCC-97H
ctrl sora PRI S+P
170-
130-
100-
70-
Active b-catenin
170-
130-
100-
70-
55-
MHCC-97H
ctrl sora PRI S+P
Hep3B
ctrl sora PRI S+P
HepG2
ctrl sora PRI S+P
c-myc
c-myc 1:1000 M 30ug erfu
HepG2
ctrl sora PRI S+P
MHCC-97H
ctrl sora PRI S+P
Hep3B
ctrl sora PRI S+P
70-
55-
p-AKT(ser473)

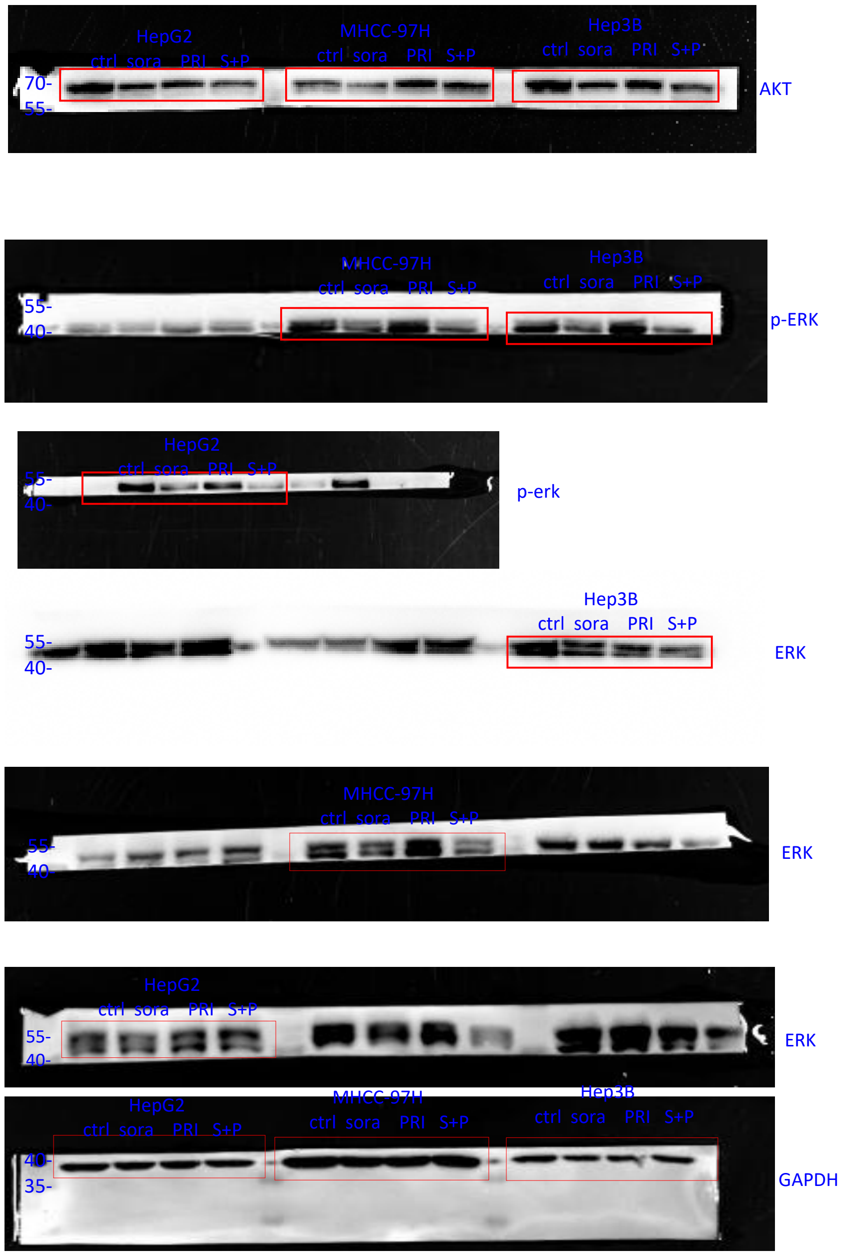
HepG2
ctrl sora PRI S+P
MHCC-97H
ctrl sora PRI S+P
Hep3B
ctrl sora PRI S+P
70-
55-
AKT
MHCC-97H
ctrl sora PRI S+P
Hep3B
ctrl sora PRI S+P
55-
40-
p-ERK
HepG2
ctrl sora PRI S+P
55-
40-
p-erk
Hep3B
ctrl sora PRI S+P
55-
40-
ERK
MHCC-97H
ctrl sora PRI S+P
55-
40-
ERK
HepG2
ctrl sora PRI S+P
55-
40-
ERK
HepG2
ctrl sora PRI S+P
MHCC-97H
ctrl sora PRI S+P
Hep3B
ctrl sora PRI S+P
40-
35-
GAPDH

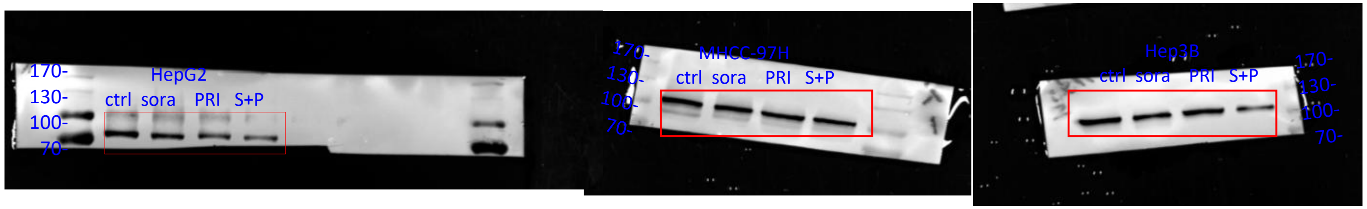

b-catenin

Figure 5G

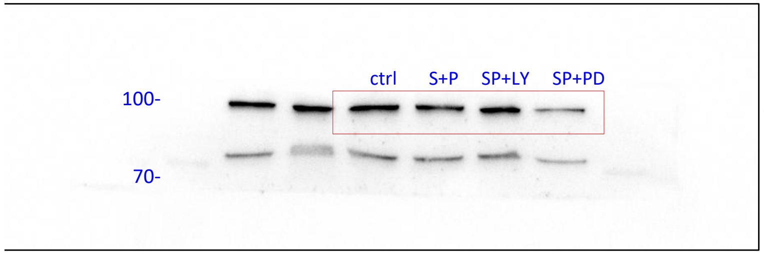

Active
b-catenin

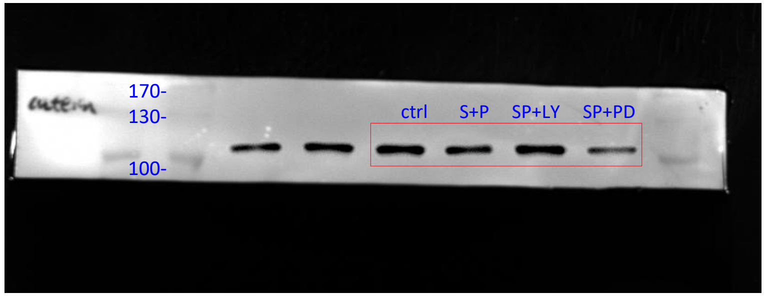

b-catenin

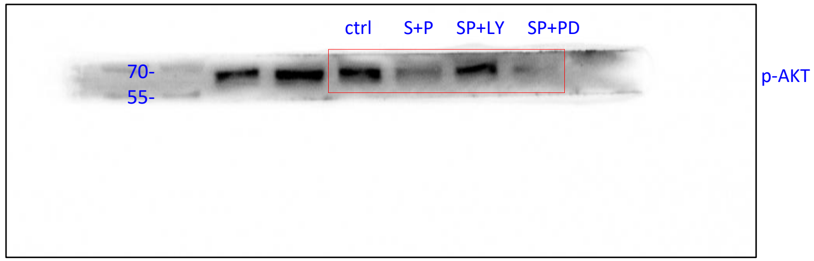

p-AKT

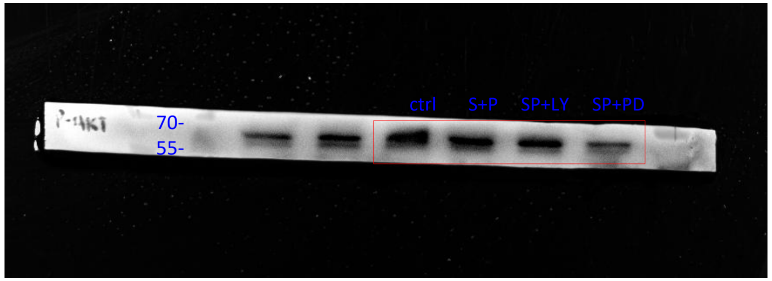

AKT

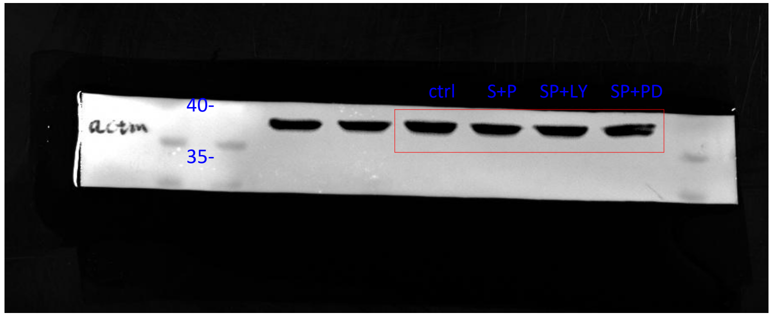

GAPDH

Figure 5H

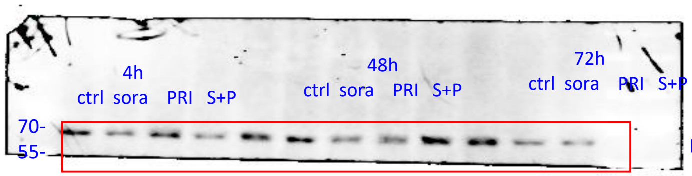

p-AKT

Figure 5I

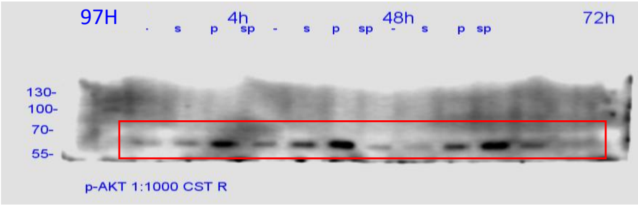

p-AKT

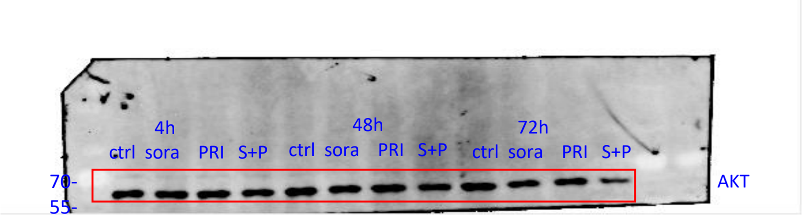

AKT

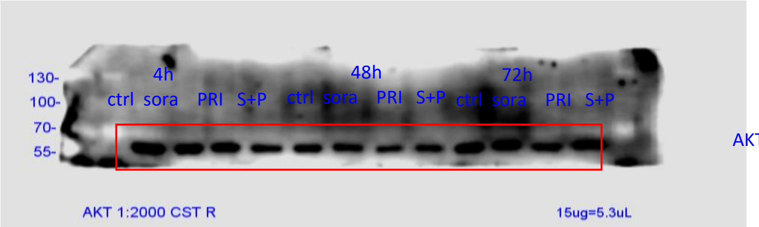

AKT

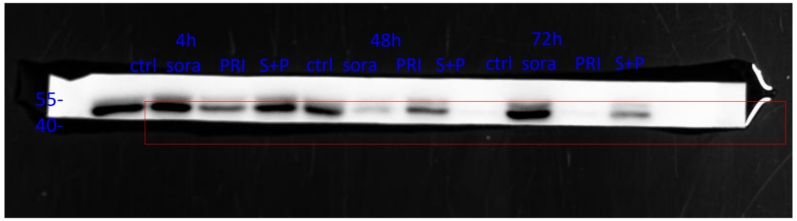

p-ERK

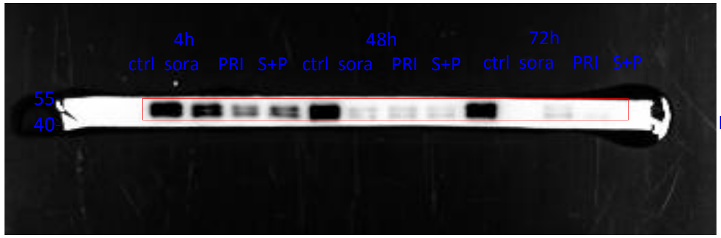

p-ERK

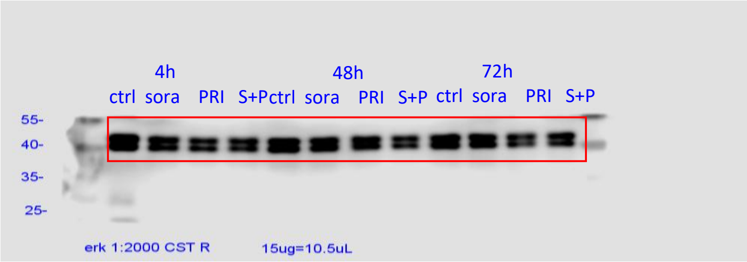

ERK

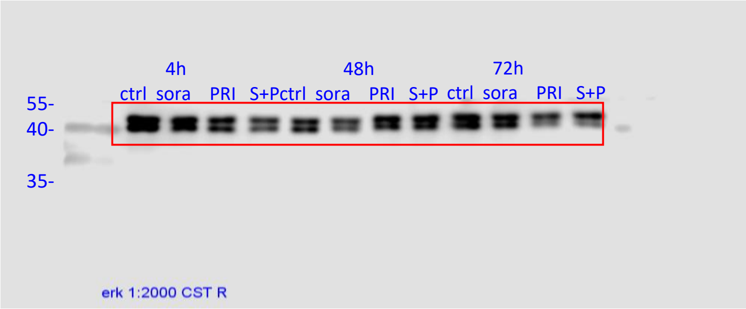

ERK

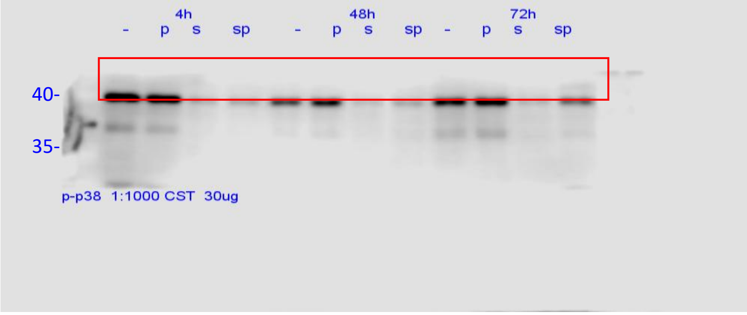

p-p38

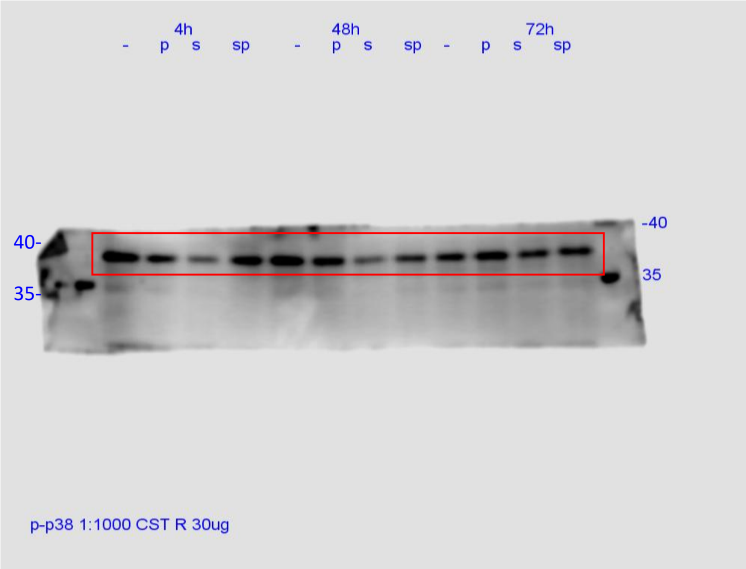

p-p38

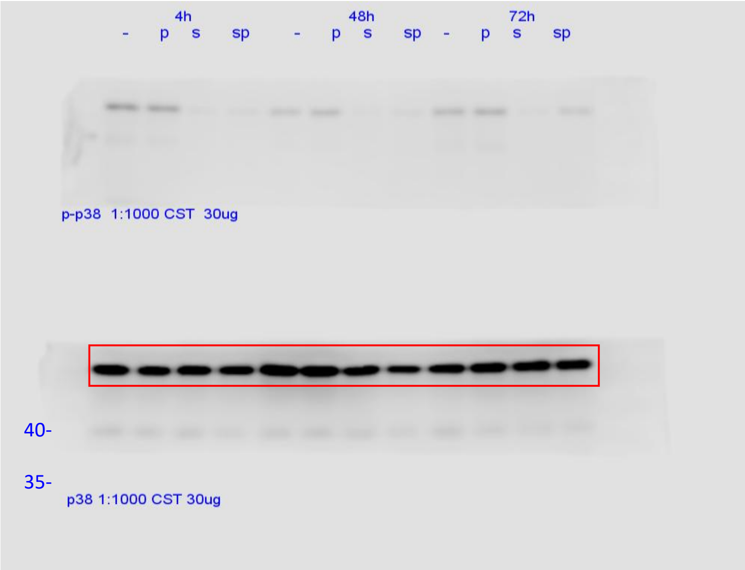

p38

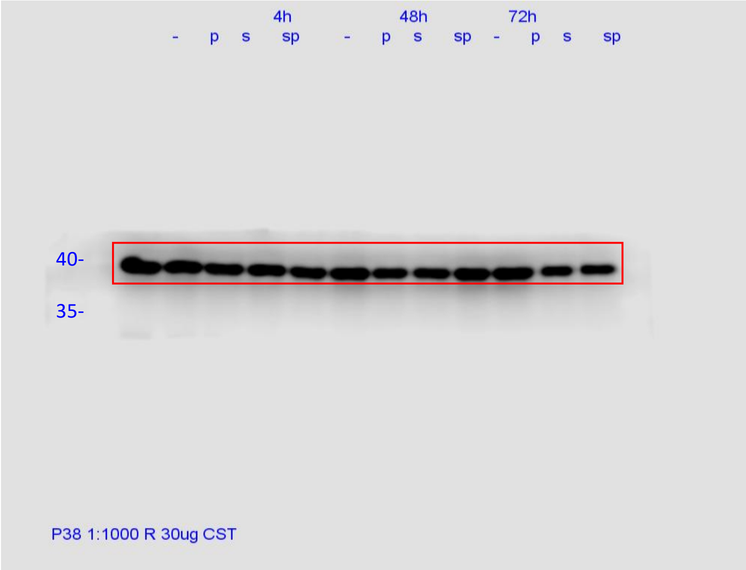

p38

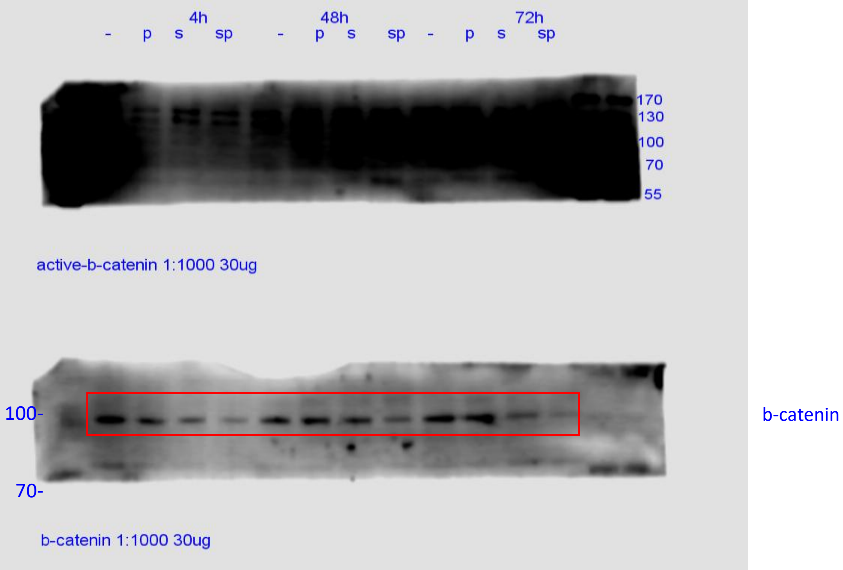
4h 48h 72h
- p s sp - p s sp - p s sp
170
130
100
70
55
active-b-catenin 1:1000 30ug
100-
70-
b-catenin
b-catenin 1:1000 30ug

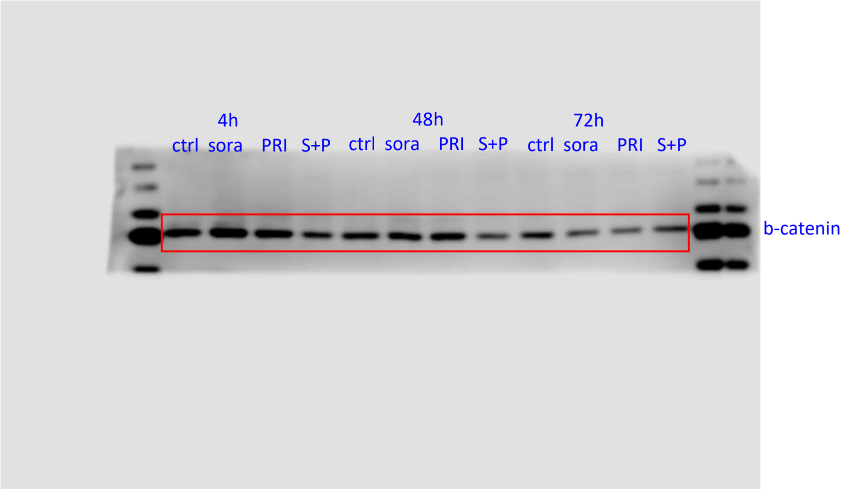
4h 48h 72h
ctrl sora PRI S+P ctrl sora PRI S+P ctrl sora PRI S+P
b-catenin

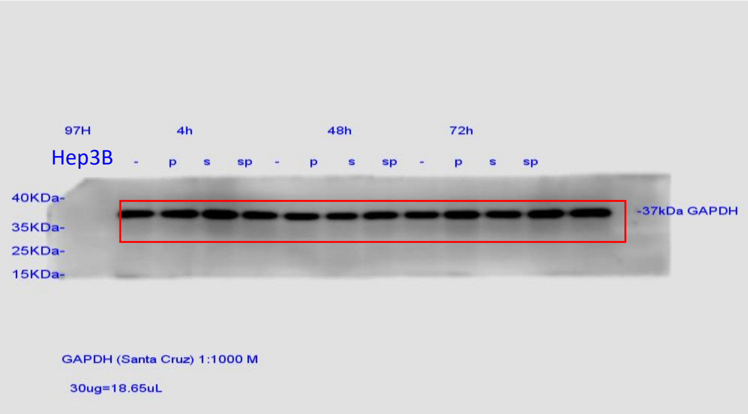
97H 4h 48h 72h
Hep3B - p s sp - p s sp - p s sp
40KDa-
35KDa-
25KDa-
15KDa-
-37kDa GAPDH
GAPDH (Santa Cruz) 1:1000 M
30ug=18.65uL

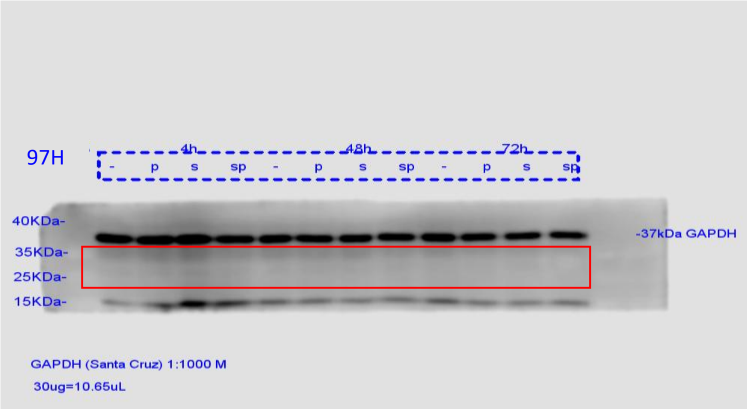
97H
4h 48h 72h
- p s sp - p s sp - p s sp
40KDa-
35KDa-
25KDa-
15KDa-
-37kDa GAPDH
GAPDH (Santa Cruz) 1:1000 M
30ug=10.65uL

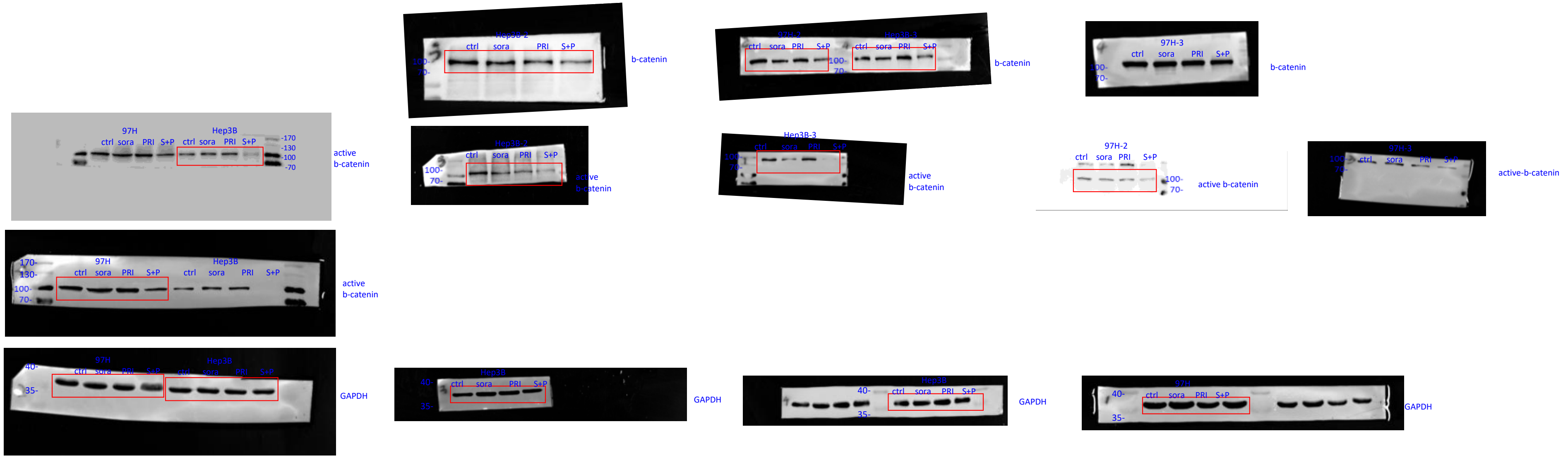

Hep3B-2
ctrl sora PRI S+P
b-catenin
97H-2
Hep3B-3
ctrl sora PRI S+P ctrl sora PRI S+P
b-catenin
97H-3
ctrl sora PRI S+P
b-catenin
97H
Hep3B
ctrl sora PRI S+P ctrl sora PRI S+P
-170
-130
-100
-70
active
b-catenin
Hep3B-2
ctrl sora PRI S+P
100-
70-
active
b-catenin
Hep3B-3
ctrl sora PRI S+P
active
b-catenin
97H-2
ctrl sora PRI S+P
100-
70-
active b-catenin
97H-3
ctrl sora PRI S+P
active-b-catenin
170-
130-
100-
70-
97H
Hep3B
ctrl sora PRI S+P ctrl sora PRI S+P
active
b-catenin
40-
35-
97H
Hep3B
ctrl sora PRI S+P ctrl sora PRI S+P
GAPDH
Hep3B
ctrl sora PRI S+P
40
35
GAPDH
Hep3B
40-
35-
ctrl sora PRI S+P
GAPDH
97H
40-
35-
ctrl sora PRI S+P
GAPDH

	time	status	sex	bcatenin	cirrhosis	Vascular_\	LNM	Distant_m	BCLC	AFP	Tbil	ALT	AST	HBV	History_of	Size	Adjuvant_	numbers	b-catenin	b-catenin	b-catenin	b-catenin	b-catenin_cytosolic score
309294	2	1	male	Positive	Yes	No	No	No	B	=<20	>19	=<50	=<40	Positive	No	>5	No	=<1	low	low	8	6	8
433227	2.5	1	male	Positive	No	No	No	No	B	>20	>19	=<50	>40	Negative	Yes	NA	Yes	=<1	low	low	12	6	12
311845	3.4	1	male	Positive	No	No	No	No	B	>20	>19	=<50	>40	Positive	No	=<5	No	=<1	low	low	4	3	4
334817	4	1	male	Positive	Yes	Yes	No	No	C	=<20	>19	>50	>40	Positive	No	>5	Yes	>1	high	high	8	2	8
278599	4.4	1	female	Negative	Yes	No	Yes	Yes	C	=<20	=<19	=<50	>40	Negative	Yes	NA	No	NA	low	low	4	2	4
308167	5.9	1	male	Positive	No	No	No	No	B	>20	=<19	=<50	=<40	Positive	Yes	=<5	Yes	=<1	high	high	1	1	0
253894	6.5	1	male	Positive	Yes	No	Yes	Yes	C	>20	=<19	>50	>40	Positive	Yes	NA	Yes	>1	high	high	15	1	15
318505	6.6	1	male	Positive	Yes	No	No	No	B	>20	>19	>50	>40	Positive	No	>5	No	>1	low	low	4	1	4
376277	9.1	1	female	Negative	Yes	Yes	No	No	C	>20	=<19	=<50	=<40	Negative	Yes	=<5	Yes	>1	high	high	3	1	3
328173	9.9	1	male	Positive	No	Yes	No	No	C	=<20	=<19	>50	>40	Positive	No	=<5	No	>1	high	high	8	1	8
498633	10.4	1	male	Negative	Yes	No	No	No	B	>20	=<19	=<50	=<40	Positive	No	=<5	No	=<1	high	high	10	1	10
296063	10.7	1	male	Negative	Yes	No	No	No	B	>20	>19	>50	>40	Positive	Yes	=<5	Yes	>1	high	high	8	1	8
344967	11.8	1	male	Negative	Yes	No	No	Yes	C	=<20	=<19	=<50	>40	Positive	Yes	=<5	No	=<1	high	high	2	0	2
351322	15.3	1	male	Negative	No	Yes	No	Yes	C	>20	=<19	=<50	>40	Positive	No	>5	No	>1	high	high	8	0	8
239360	17.2	1	male	Negative	No	No	No	No	B	>20	>19	=<50	>40	Negative	Yes	NA	No	>1	low	low	6	0	6
391621	17.2	1	male	Positive	Yes	No	Yes	Yes	C	>20	>19	>50	=<40	Positive	Yes	NA	No	NA	high	high	8	0	8
389293	18.9	1	male	Negative	Yes	No	Yes	Yes	C	>20	>19	=<50	=<40	Positive	Yes	>5	No	=<1	high	high	15	0	15
271851	19.1	1	male	Negative	Yes	Yes	No	No	C	>20	>19	>50	=<40	Positive	No	NA	Yes	=<1	high	high	12	0	12
277258	19.7	1	male	Positive	No	Yes	Yes	Yes	C	=<20	=<19	=<50	=<40	Negative	No	>5	Yes	>1	high	high	4	0	4
318782	25.3	1	male	Positive	Yes	No	No	Yes	C	=<20	>19	>50	>40	Positive	No	NA	No	>1	high	high	8	0	8
306093	25.5	1	female	Negative	No	No	No	No	B	=<20	>19	>50	>40	Negative	No	>5	No	=<1	high	high	10	0	10
359224	26.1	1	male	Negative	No	No	No	No	B	>20	=<19	>50	>40	Negative	Yes	NA	No	NA	high	high	10	0	10
275391	27.3	1	male	Positive	No	No	No	No	B	=<20	>19	>50	>40	Positive	No	NA	No	NA	low	low	10	0	10
328508	27.5	1	male	Negative	No	No	No	No	B	=<20	>19	>50	>40	Negative	No	>5	No	=<1	low	low	2	0	3
242977	29.3	1	male	Negative	No	No	No	No	B	>20	=<19	>50	>40	Positive	No	NA	No	NA	high	high	8	0	8
433155	29.4	1	male	Negative	Yes	No	No	No	B	=<20	>19	>50	>40	Positive	Yes	>5	No	=<1	high	high	10	0	10
323210	31.1	1	female	Negative	Yes	No	No	No	B	>20	=<19	>50	=<40	Positive	Yes	>5	No	=<1	high	high	15	0	15
303004	31.8	1	male	Negative	Yes	No	Yes	Yes	C	=<20	>19	>50	>40	Positive	No	>5	No	=<1	high	high	12	0	12
273446	31.9	1	female	Negative	No	No	No	No	B	=<20	>19	=<50	=<40	Negative	Yes	=<5	No	>1	high	high	10	0	10
344706	34.2	1	male	Negative	Yes	No	Yes	Yes	C	>20	=<19	=<50	=<40	Positive	Yes	NA	No	>1	low	low	4	0	4
259995	34.7	1	male	Negative	No	No	No	Yes	C	>20	>19	=<50	=<40	Negative	Yes	NA	No	NA	high	high	8	0	8
299610	37.5	1	male	Negative	Yes	No	No	No	C	=<20	>19	>50	=<40	Positive	Yes	=<5	No	>1	low	low	1	0	1
398406	41	1	male	Negative	Yes	No	No	No	B	>20	=<19	=<50	=<40	Negative	Yes	NA	No	NA	high	high	6	0	6
391464	43.3	1	male	Negative	Yes	No	No	No	B	>20	>19	>50	>40	Positive	Yes	NA	No	NA	high	high	8	0	8
385203	45.8	1	female	Negative	Yes	No	No	No	C	>20	>19	>50	>40	Positive	Yes	NA	No	NA	low	low	4	0	4
273649	56.1	1	male	Negative	Yes	No	No	No	B	>20	=<19	=<50	=<40	Positive	Yes	NA	Yes	>1	high	high	15	0	15
294011	59.3	1	male	Negative	Yes	Yes	No	No	C	>20	>19	>50	>40	Positive	No	>5	No	=<1	low	low	3	0	3
335320	63.8	1	female	Negative	No	Yes	Yes	Yes	C	=<20	>19	=<50	=<40	Positive	No	>5	Yes	>1	high	high	15	0	15
314828	66.4	1	female	Negative	Yes	No	No	No	B	>20	>19	=<50	=<40	Positive	Yes	NA	No	>1	high	high	8	0	8
325052	67.7	1	male	Negative	No	No	No	No	B	=<20	>19	>50	>40	Positive	Yes	NA	No	NA	high	high	8	0	8
319511	70.2	1	male	Negative	Yes	Yes	No	No	C	=<20	>19	=<50	=<40	Positive	Yes	NA	No	>1	low	low	4	0	4

sFig 5A

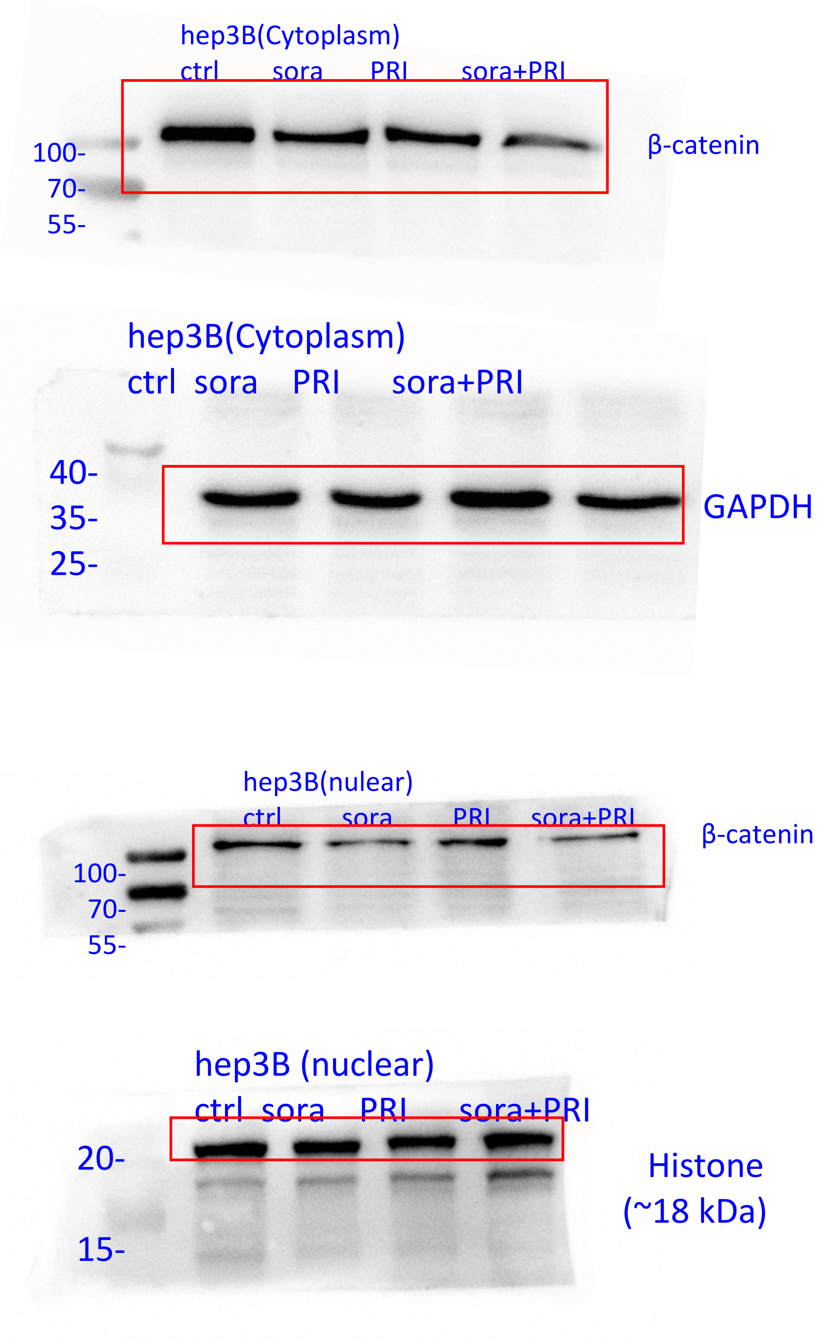
hep3B(Cytoplasm)
ctrl sora PRI sora+PRI
100-
70-
55-
β-catenin
hep3B(Cytoplasm)
ctrl sora PRI sora+PRI
40-
35-
25-
GAPDH
hep3B(nulear)
ctrl sora PRI sora+PRI
100-
70-
55-
β-catenin
hep3B (nuclear)
ctrl sora PRI sora+PRI
20-
15-
Histone
(~18 kDa)
